# High-Throughput Microsatellite Markers Development for Genetic Characterization of Emerging *Sporothrix* Species

**DOI:** 10.3390/jof9030354

**Published:** 2023-03-14

**Authors:** Luiza Chaves de Miranda Leonhardt Losada, Ruan Campos Monteiro, Jamile Ambrósio de Carvalho, Ferry Hagen, Matthew C. Fisher, Bram Spruijtenburg, Jacques F. Meis, Theun de Groot, Sarah Santos Gonçalves, Ricardo Negroni, Rui Kano, Alexandro Bonifaz, Zoilo Pires de Camargo, Anderson Messias Rodrigues

**Affiliations:** 1 Laboratory of Emerging Fungal Pathogens, Department of Microbiology, Immunology, and Parasitology, Discipline of Cellular Biology, Federal University of São Paulo (UNIFESP), São Paulo 04023062, Brazil; 2 Department of Medicine, Discipline of Infectious Diseases, Federal University of São Paulo (UNIFESP), São Paulo 04023062, Brazil; 3 Department of Medical Mycology, Westerdijk Fungal Biodiversity Institute, Uppsalalaan 8, 3584 CT Utrecht, The Netherlands; 4 Institute for Biodiversity and Ecosystem Dynamics, University of Amsterdam, Sciencepark 904, 1098 XH Amsterdam, The Netherlands; 5 Department of Medical Microbiology, University Medical Center Utrecht, Heidelberglaan 100, 3584 CX Utrecht, The Netherlands; 6 Medical Research Council Center for Global Infectious Disease Analysis, Department of Infectious Disease Epidemiology, School of Public Health, Imperial College London, London W2 1PG, UK; 7 Department of Medical Microbiology and Infectious Diseases, Canisius-Wilhelmina Hospital, 6532 SZ Nijmegen, The Netherlands; 8 Center of Expertise in Mycology Radboud University Medical Center/Canisius-Wilhelmina Hospital, 6532 SZ Nijmegen, The Netherlands; 9 Department I of Internal Medicine, Faculty of Medicine, University of Cologne, and Excellence Center for Medical Mycology, University Hospital Cologne, 50931 Cologne, Germany; 10 Infectious Diseases Postgraduate Program, Center for Research in Medical Mycology, Federal University of Espírito Santo (UFES), Vitória 29043900, Brazil; 11 Mycology Unit of the Infectious Diseases Hospital Francisco Javier Muñiz, Reference Center of Mycology of Buenos Aires City, Uspallata, Buenos Aires 2272, Argentina; 12 Teikyo University Institute of Medical Mycology (TIMM), 359 Otsuka, Tokyo 192-0395, Japan; 13 Dermatology Service, Mycology Department, Hospital General de México, “Dr. Eduardo Liceaga”, Balmis 148, Colonia Doctores, Mexico City 03020, Mexico

**Keywords:** zoonosis, microsatellite, simple sequence repeats, SSR, epidemiology, *Ophiostomatales*, AMOVA, sporotrichosis, linkage disequilibrium, population genetics

## Abstract

Sporotrichosis is the main subcutaneous mycosis worldwide transmitted by animal or plant vectors and often escalates to outbreaks or epidemics. The current cat-transmitted sporotrichosis driven by *Sporothrix brasiliensis* has become a significant public health issue in South America. Transmission dynamics remain enigmatic due to the lack of development of polymorphic markers for molecular epidemiological analysis. This study used a high-throughput mining strategy to characterize simple sequence repeat (SSR) markers from *Sporothrix* genomes. A total of 118,140–143,912 SSR loci were identified (82,841–98,369 unique markers), with a 3651.55–3804.65 SSR/Mb density and a majority of dinucleotides motifs (GC/CG). We developed a panel of 15 highly polymorphic SSR markers suitable for genotyping *S. brasiliensis*, *S. schenckii,* and *S. globosa*. PCR amplification revealed 240 alleles in 180 *Sporothrix* isolates with excellent polymorphic information content (*PIC* = 0.9101), expected heterozygosity (*H* = 0.9159), and discriminating power (*D* = 0.7127), supporting the effectiveness of SSR markers in uncovering cryptic genetic diversity. A systematic population genetic study estimated three clusters, corresponding to *S. brasiliensis* (population 1, n = 97), *S. schenckii* (population 2, n = 49), and *S. globosa* (population 3, n = 34), with a weak signature of mixed ancestry between populations 1 and 2 or 3 and 2. Partitioning of genetic variation via AMOVA revealed highly structured populations (ΦPT = 0.539; Nm = 0.213; *p* < 0.0001), with approximately equivalent genetic variability within (46%) and between (54%) populations. Analysis of SSR diversity supports Rio de Janeiro (RJ) as the center of origin for contemporary *S. brasiliensis* infections. The recent emergence of cat-transmitted sporotrichosis in northeastern Brazil indicates an RJ-Northeast migration resulting in founder effects during the introduction of diseased animals into sporotrichosis-free areas. Our results demonstrated high cross-species transferability, reproducibility, and informativeness of SSR genetic markers, helping dissect deep and fine-scale genetic structures and guiding decision making to mitigate the harmful effects of the expansion of cat-transmitted sporotrichosis.

## 1. Introduction

The genus *Sporothrix* (*Ophiostomatales*) includes 53 species [1]. Although most *Sporothrix* species present little pathogenicity towards the warm-blooded vertebrate host and are embedded in an environmental clade associated with soil, plant debris, and insects, a clinical cluster has recently emerged. This cluster contains species such as *S. brasiliensis*, *S. schenckii,* and *S. globosa* with increased virulence attributes, causing chronic cutaneous and subcutaneous mycosis in humans and animals [2]. 

Classically, sporotrichosis is an implantation mycosis where *Sporothrix* propagules present in the soil or decaying wood are traumatically inoculated into the host’s subcutaneous tissue [3]. The classical transmission route (environment–human) occurs as an occupational disease among gardeners, rural workers, and loggers [4]. Outbreaks due to *S. globosa* and *S. schenckii* occur worldwide, mainly via the classical sapronotic route (acquisition from the environment). In Asia, especially in China, India, and Japan, the leading agent is *S. globosa* [5]. In Africa and the Americas, especially in the USA, Mexico, and part of South America (except Brazil), *S. schenckii* is the primary etiological agent [6].

Alternatively, the disease can occur in dogs, cats, horses, cows, camels, dolphins, goats, mules, birds, pigs, rats, and armadillos [7,8]. Of relevance, epizootics among domestic cats are common, leading to zoonotic sporotrichosis in endemic regions. In recent decades, Brazil has been experiencing the most significant cat-transmitted sporotrichosis outbreaks in urban areas (e.g., cat–cat and cat–human transmission) [9]. *Sporothrix brasiliensis* is the prevalent etiologic agent during epizooties reported mainly in Brazil’s South and Southeast regions. With the absence of adequate public policies to control the spread of the outbreaks, cases have begun to emerge in Northeast Brazil [10,11,12] and, more recently, in Argentina, Chile, Paraguay [13,14,15,16,17,18], and in the UK [19,20]. 

Judging from the epidemiological scenarios above, the primary agents of sporotrichosis are constantly on the move, causing outbreaks worldwide [21]. Moreover, studies related to phylogeny [22], chromosomal polymorphism [23], virulence [24], epidemiology [25], and drug susceptibility [26,27] have revealed significant differences among *Sporothrix* species. Therefore, epidemiological surveillance studies require practical molecular tools to quickly and reliably distinguish among related isolates and explore transmission dynamics during outbreaks and epidemics. Classical typing methods, such as multilocus sequencing analysis (MLSA) [28], DNA barcoding [29], restriction fragment length polymorphism (RFLP) [30], and random amplified polymorphic DNA (RAPD) [31], have historically been essential contributors to our understanding of the natural history and epidemiology of infections caused by *Sporothrix* species. Although these methods are inexpensive, they usually lack resolving power. On the other hand, a higher resolution can be achieved by sequencing whole genomes [32,33], but the high cost associated is a limiting component to exploring a significant number of specimens during outbreaks.

Molecular markers such as the amplified fragment length polymorphism (AFLP) [34,35] and simple sequence repeats (SSRs, also known as microsatellites) [36] are valuable tools for genetic surveillance due to low-cost, high-resolution, reproducibility, and easy implementation. Moreover, SSRs have several advantages over other molecular markers due to their codominant inheritance and highly polymorphic nature, allowing the recognition of numerous alleles at a single locus. Nevertheless, microsatellite markers have not yet been developed for all medically relevant *Sporothrix* species and are restricted to *S. globosa* [36].

Therefore, we used a high-throughput in silico mining strategy to characterize SSR markers from available *Sporothrix* genomes [37,38,39,40,41]. Through extensive genome analysis, we developed 15 new SSR markers covering all emerging species in the clinical clade to test in vitro epidemiological and evolutionary hypotheses. Different SSR markers were successfully combined in multiplex panels to minimize costs and achieve higher resolution in population genetic applications.

## 2. Materials and Methods

### 2.1. Sporothrix Isolates and DNA Extraction

This study included 180 *Sporothrix* isolates (*S. brasiliensis*, n = 97; *S. schenckii*, n = 49; *S. globosa*, n = 34) obtained from clinical lesions of patients with varying degrees of human disease severity (n = 132), animals (n = 44) or environmental sources (n = 4). The isolates were identified down to species level by comparison with the descriptions presented by Rodrigues et al. [7,25,42,43] and were stored at room temperature in slant cultures on Sabouraud dextrose agar (Difco Laboratories, Detroit, MI, USA) [44].

Fungal DNA was obtained and purified directly from 14-day-old monoconidial colonies on Sabouraud slants by following the Fast DNA kit protocol (MP Biomedicals, Irvine, CA, USA), as previously described [7]. We used a *Sporothrix* species-specific PCR assay targeting the calmodulin-encoding gene, as previously described [45], or a multiplex qPCR test targeting the β-tubulin [46] to characterize all isolates down to the species level. All experiments included reference strains representing the major evolutionary groups in *Sporothrix* (Supplementary Table S1).

### 2.2. In Silico Genomic Characterization of Simple Sequence Repeats

To predict SSR markers, the genome sequences of eight *Sporothrix* isolates (Supplementary Table S2) were retrieved from NCBI (https://www.ncbi.nlm.nih.gov/genome/) (accessed on 15 March 2018) and analyzed in silico. Genomic characterization was performed using Krait [47] and GMATA [48] and in silico mined for SSRs with thresholds of a minimum length of 2 bp, maximum length of 10 bp, and minimum repeat times of motif of three, four, or five times was considered. SSR-containing sequences were retrieved from *Sporothrix* genomes, as well as short 5′ and 3′ flanking regions (~500 bp each). Retrieved sequences were used as templates for BLAST searches using a local BLAST database containing *Sporothrix* genomes implemented in the CLC Genomics Workbench v.9.0.1 (Supplementary Table S2). 

### 2.3. Primer Design

Sequences retrieved from the BLAST search were aligned with MAFFT v7 [49], and retrieved alignments were corrected manually with the MEGA v7 software [50] to avoid mispairing bases. SSR flanking regions were used to identify conserved regions, which could be used for primer design considering all emerging species in the clinical clade. We chose short, informative regions with 18–25 nucleotide variations based on nucleotide polymorphisms. The software Primer3 [51] (http://primer3.wi.mit.edu/) (accessed on 20 March 2018) was used to evaluate melting temperatures, %GC contents, dimer sequences, and mismatches in candidate sequences. Next, candidate primers were assessed with the Mfold software [52] for potential secondary structures, which could reduce amplification efficiency. Finally, we selected primer sets that would generate different product sizes to facilitate multiplex assays. For fluorescent SSR, we used forward primers labeled with 6-carboxyfluorescein (FAM; 520 nm), 2′-chloro-phenyl-1,4-dichloro-6-carboxyfluorescein (VIC; 555 nm), or benzofluorotrichlorocarboxy-fluorescein (NED; 576 nm), and unlabeled reverse primers.

### 2.4. PCR Optimization and Capillary Electrophoresis

Total DNA extracted from *Sporothrix* species was used as a PCR template. Each multiplex PCR was performed with three primer sets (10 pmol/μL; Integrated DNA Technologies, San Diego, CA, USA) in a final volume of 25 μL (see Supplementary Figure S1), including 12.5 μL of 2× PCR Master Mix buffer, which contained 3 mM MgCl_2_, 400 mM of each dNTP, and 50 U/mL of Taq polymerase (Promega Corporation, Madison, WI, USA), ultra-pure water, and 1 μL of target DNA (50 ng/μL). PCRs were performed in an Eppendorf Mastercycler Pro system (Eppendorf, Hamburg, Germany). PCR conditions were as follows: a first step of 5 min at 95 °C, followed by 35 cycles of 1 min at 94 °C, 1 min at 60 °C, and 2 min at 72 °C, followed by one step of 15 min at 72 °C (Supplementary Figure S1). Amplified SSR fragments were diluted (50×) and resolved by capillary electrophoresis with a SeqStudio Analyzer alongside a LIZ600 internal size standard (Applied Biosystems, Foster City, CA, USA) at the Laboratory of Emerging Fungal Pathogens (Federal University of São Paulo, São Paulo, Brazil) under previously described conditions [34]. At least two independent electropherograms were obtained for each SSR marker.

### 2.5. Bioinformatic Analysis

The sizes of the amplified fragments were determined using GeneMapper v6 software (ThermoFisher Scientific, Waltham, MA, USA), and a matrix of codominant data was then constructed. In addition, the raw data were imported into BioNumerics v7.6 software (Applied Maths, Sint-Martens-Latem, Belgium). To reduce scoring errors, each electropherogram was carefully examined to exclude low confidence peaks (stutter peaks and other artifacts, pull-up peaks, plus-adenine peaks), setting the minimum threshold at 100 relative fluorescence units (RFU) and considering only peaks with sizes in the expected range. Automated fragment matching was implemented on all fingerprint profiles within the comparison, disregarding uncertain bands, and with the optimization and position tolerances for picking fragments established for 0.10%.

Distance-based techniques were used to assess relationships among *Sporothrix* isolates using BioNumerics v7.6. The band-based Dice similarity coefficient was used to compute pairwise genetic distances [53] combined with a “Fuzzy logic” option. Dendrograms were assumed using the unweighted pair group mean arithmetic method (UPGMA) [54]. To assess the consistency of a given cluster, we used the cophenetic correlation coefficient and its standard deviation [55]. The Pearson product-moment correlation coefficient (Pearson correlation) [56] and the global similarity levels were used to calculate the presence of topological congruence among SSR multiplexes.

Dimensionality reduction methods such as principal component analysis (PCA) and multidimensional scaling (MDS) were applied to generate three-dimensional plots with *Sporothrix* isolates scattered according to their relationship. A discriminant analysis (PCDA) was used to calculate the best discriminating components for *Sporothrix* groups. Default settings were applied for PCA, MDS, and PCDA, subtracting the average for characters. Additionally, the Self-Organizing Map (SOM), a standard artificial neural network algorithm in the unsupervised learning category, was employed to categorize SSR data in a two-dimensional map corresponding to their relatedness [57]. The size of the Kohonen map was set to 100 (i.e., the neural network nodes in each direction), using the alleles information (characters) and the similarity matrix. 

The evolutionary relations among all *Sporothrix* genotypes and the most likely transmission network were investigated using SSR-derived Minimum Spanning Trees (MSTs) as previously described [34,58]. All figures were exported and treated using Corel Draw X8 (Alludo, Ottawa, ON, Canada).

### 2.6. Genetic Diversity Analysis and Linkage Disequilibrium

To assess the efficiency of each SSR marker evaluated here, the following genetic parameters for codominant markers were determined: polymorphic information content (*PIC*) [59], expected heterozygosity (*H*) [60], effective multiplex ratio (*E*) [61], arithmetic mean heterozygosity (*H_avp_*) [61], marker index (*MI*) [61,62], and discriminating power (*D*) [63]. The Simpson’s diversity index was used to measure the diversity of *Sporothrix* genotypes in each group [64]. Additionally, linkage disequilibrium (*LD*) was assessed by the standardized disequilibrium coefficient (*D*’), as well as squared allele-frequency correlations (r^2^) between pairs of polymorphic loci using the software package TASSEL v5 [65]. A two-sided Fisher’s Exact test determined *p*-values for each r^2^ estimate, and loci were considered to be in significant *LD* when *p* < 0.001 [66].

### 2.7. Structure Analysis

Bayesian analysis of population genetic structure was assessed in Structure v2.3.4 [67] applying the admixture model, allowing alpha to be inferred and assuming correlated allele frequencies procedure, using a burn-in period of 10,000 Markov Chain Monte Carlo (MCMC) replications followed by 100,000 sampling replications, with 20 independent runs performed for *K* values one to fifteen. We calculated the posterior distribution of alpha to ensure all chains for *K* values 2–15 converged. All data were evaluated using the method of Evanno and colleagues as implemented in StructureHARVESTER v0.6.94 [68,69] to establish the optimal number of clusters (*K*). Consensus population distributions were obtained with CLUMPP v1.1.2 [70], and final plots were exported and treated using Corel Draw X8. 

### 2.8. Analysis of Molecular Variance (AMOVA) 

A matrix of codominant data (codon-genotypic SSR) was employed to analyze molecular variance using GenAlex v6.5 [71,72]. The genetic differentiation among populations was determined using ΦPT (PhiPT), and the gene flow (Nm) between populations was calculated. These measures allow intra-individual variation to be suppressed and are ideal for comparing codominant data with 9999 permutations [73]. 

### 2.9. Characterization of the Mating-Type Idiomorphs and Mitochondrial DNA Typing

A duplex PCR using primers targeting the *MAT1-1* or *MAT1-2* region was used to determine the mating-types idiomorphs, as described before [74]. *Sporothrix* DNA (50 ng) was used as a template for PCRs including two sets of primers: SPMAT1-1F and SPMAT1-1R, which selectively amplify a 673 bp portion from the α box region of the *MAT1-1* locus, or SPMAT1-2F and SPMAT1-2R, which selectively amplify a 291 bp fragment from the HMG domain gene, present in the *MAT1-2* idiomorph. Mitochondrial DNA (mtDNA) typing in *Sporothrix* was achieved using the primers pair 975–8038F and 975–9194R that amplify an intergenic region between the *ATP9* and *COX2* genes [75]. Amplicons were resolved using 1.2% agarose gel in 1× TBE buffer at 100V for 1 h at room temperature in the presence of GelRed (Biotium, Hayward, CA, USA) [76]. Amplicon size was estimated based on a comparison with 100 bp GeneRuler DNA Ladders (ThermoFisher Scientific).

## 3. Results

### 3.1. In Silico Mining of SSR Markers

**Figure 1:**
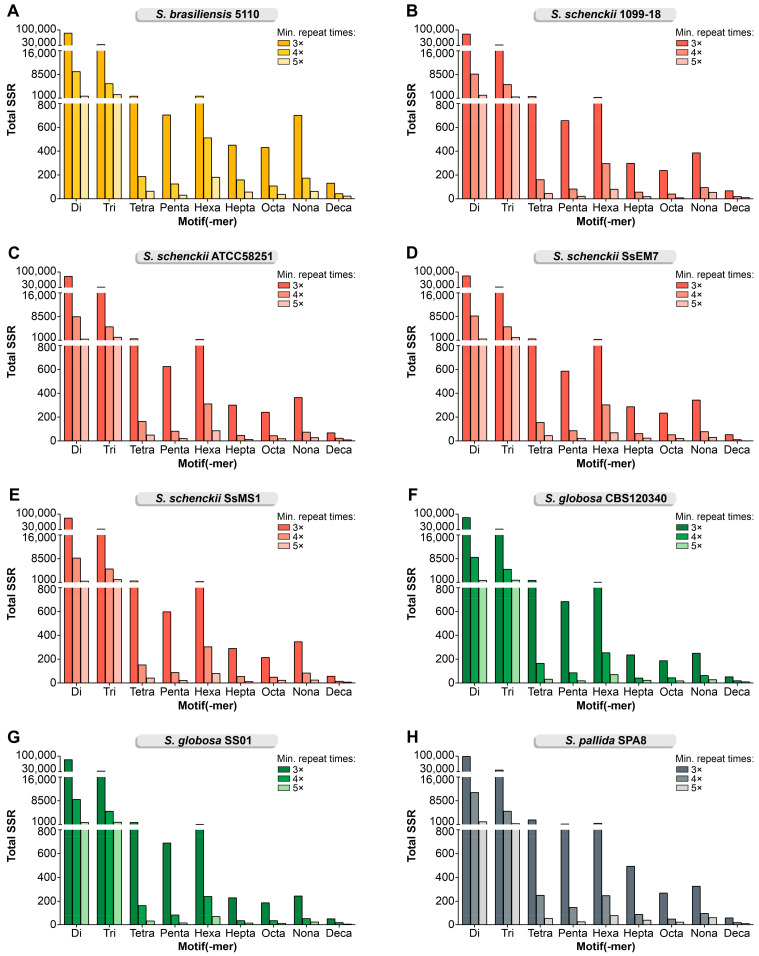
SSR markers with a minimum of 3, 4, or 5 repeats and motifs of 2–10 bp in the *Sporothrix* genomes were analyzed in GMATA using the default settings: (**A**) *S. brasiliensis* 5110; (**B**) *S. schenckii* 1099-18; (**C**) *S. schenckii* ATCC 58251; (**D**) *S. schenckii* SsEM7; (**E**) *S. schenckii* SsMS1; (**F**) *S. globosa* CBS 120340; (**G**) *S. globosa* SS01; (**H**) *S. pallida* SPA8.

The first step of our study relied on the in silico characterization of eight clinically relevant *Sporothrix* genomes (Table 1). We estimated the total number of repeats considering a range of motifs composed of 2–10 bp and with a minimum of three, four, or five repetitions per motif, finding values ranging between 118,140 and 143,912 SSR loci (mean = 124,158 ± 8.347; coefficient of variation = 6.72%) for the most tolerant scenario (minimum 3×). As expected, the simplest elements, such as dinucleotides, are the most abundant SSR in *Sporothrix* regardless of the evaluated genome (Figure 1). Increasing the motif’s complexity (composition and size) significantly reduces the frequency of these elements in the genomes considering a minimum of 4× (14,599–17,289; mean = 15,298 ± 986.7; coefficient of variation = 6.45%) and 5× (3506–4341; mean = 3707 ± 263.6; coefficient of variation = 7.10%) (Table 1).

The genomes of *S. brasiliensis* and *S. pallida* show the highest density of SSR/Mb (3803.12–3804.65). Among the species of clinical interest, *S. brasiliensis* had the highest number of SSR loci (126,286 loci), followed by *S. globosa* (122,302–122,335 loci) and *S. schenckii* (118,140–120,585 loci). Between 68 and 70% of SSR markers are documented as unique sequences, showing a large diversity of these elements. Primers were successfully designed for 98.16–99.58% of the characterized loci (Table 1). Nevertheless, our criteria for composing an SSR panel considered that (i) the markers should be polymorphic intra and interspecies; (ii) primers were designed to promote cross-species transferability; (iii) amplicons should not exceed 600 bp; (iv) forward primers were designed to be labeled in the 5’-region; therefore, sequences containing G in the 5’ region were avoided; and (v) the annealing temperature of the primers should be 60 ± 2 °C, and primers that presented secondary structures or interactions were eliminated to prioritize multiplex reactions.

**Figure 2:**
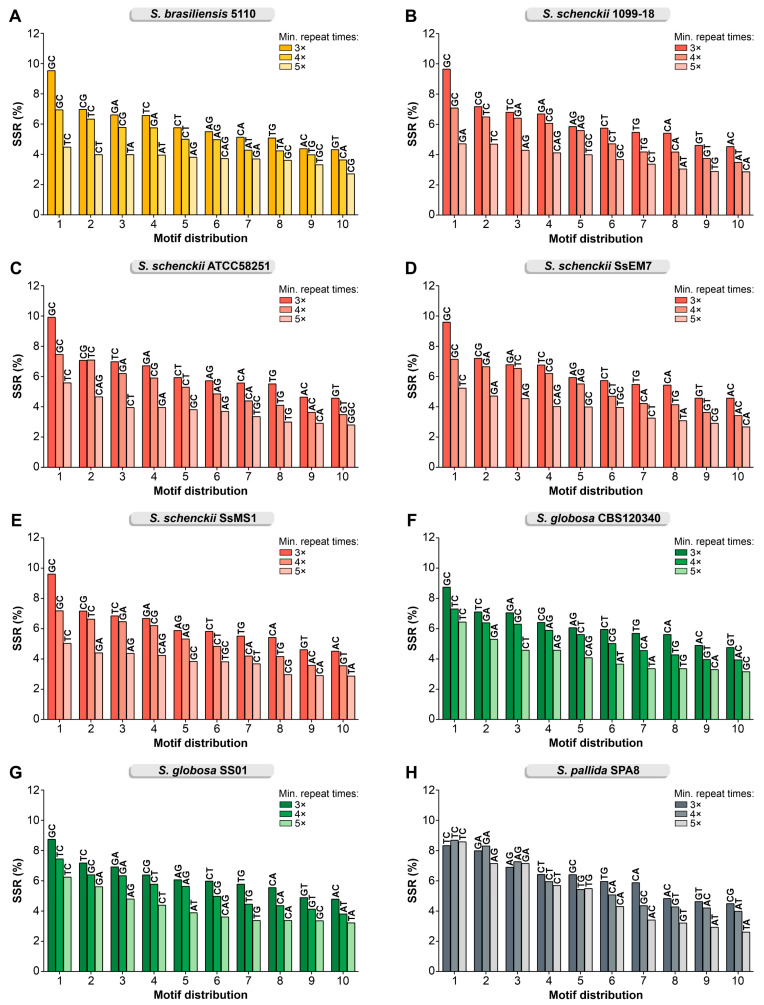
The top 10 motif distribution with a minimum of 3, 4, or 5 repeats and motifs of 2–10 bp identifies that GC/CG is the major SSR in *Sporothrix* genomes: (**A**) *S. brasiliensis* 5110; (**B**) *S. schenckii* 1099-18; (**C**) *S. schenckii* ATCC58251; (**D**) *S. schenckii* SsEM7; (**E**) *S. schenckii* SsMS1; (**F**) *S. globosa* CBS 120340; (**G**) *S. globosa* SS01; (**H**) *S. pallida* SPA8.

**Figure 3:**
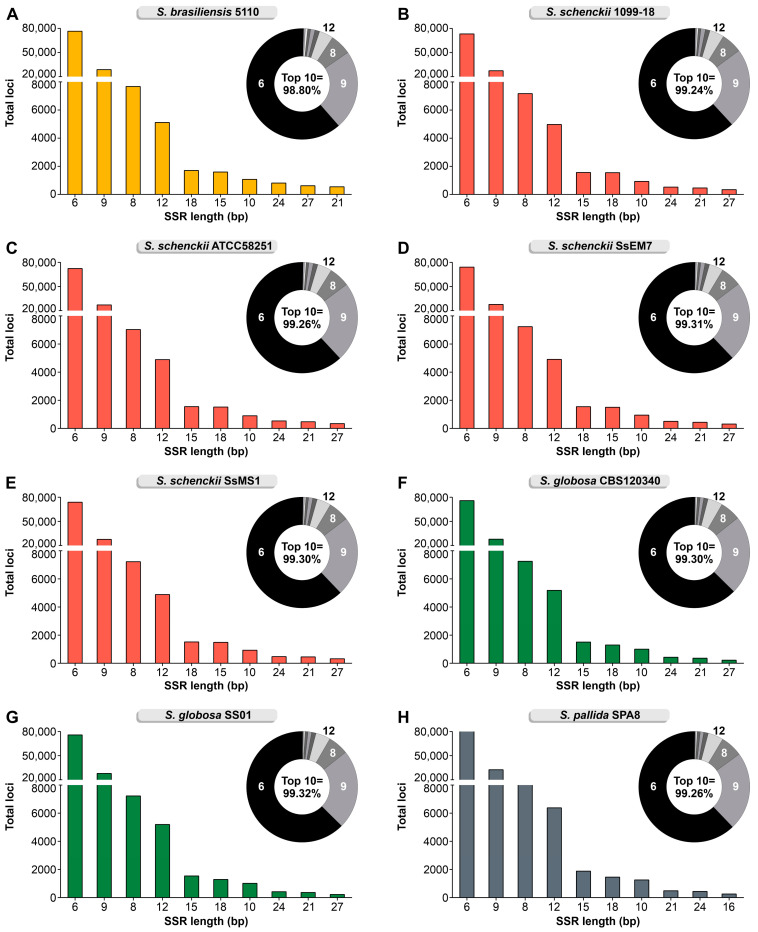
SSR length distribution with a minimum of 3 repeats and motifs of 2–10 bp shows that 6 bp, 9 bp, 8 bp, and 12 bp are the major SSR in *Sporothrix*: (**A**) *S. brasiliensis* 5110; (**B**) *S. schenckii* 1099-18; (**C**) *S. schenckii* ATCC58251; (**D**) *S. schenckii* SsEM7; (**E**) *S. schenckii* SsMS1; (**F**) *S. globosa* CBS 120340; (**G**) *S. globosa* SS01; (**H**) *S. pallida* SPA8.

For all species, the GC, CG, GA, TC, CT, AG, CA, TG, AC, and GT are the most abundant motifs representing 59.93–62.72% of the repeats in their genomes (Figure 2; minimum 3×). Repeats with a size of 6 bp (72,627–88,745 loci), 9 bp (27,324–32,710 loci), 8 bp (7042–9174 loci), 12 bp (4899–6393 loci), 18 bp (1299–1710 loci), 15 bp (1311–1889 loci), 10 bp (909–1266 loci), 24 bp (428–821 loci), 27 bp (232–626 loci), and 21 bp (232–552 loci) correspond to 98.8–99.32% of the loci characterized in *Sporothrix* (Figure 3).

**Figure 4:**
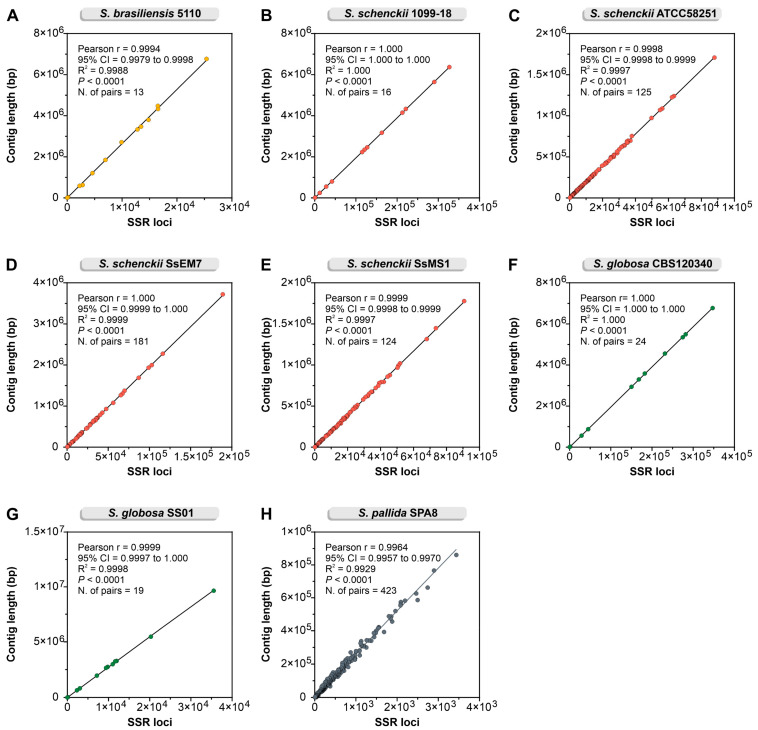
Pearson correlation analysis between SSR loci count (minimum of 3 repeats and motifs of 2–10 bp) and each contig length shows a positive correlation (R = 0.99–1.00; *p* < 0.0001) for all *Sporothrix* genomes analyzed: (**A**) *S. brasiliensis* 5110; (**B**) *S. schenckii* 1099-18; (**C**) *S. schenckii* ATCC58251; (**D**) *S. schenckii* SsEM7; (**E**) *S. schenckii* SsMS1; (**F**) *S. globosa* CBS 120340; (**G**) *S. globosa* SS01; (**H**) *S. pallida* SPA8.

The distribution of SSR loci is remarkably homogeneous throughout genomes. A positive correlation between the size of the contigs and the number of microsatellites was found for all evaluated genomes (Pearson correlation = 0.99–1.00; *p* < 0.0001) (Figure 4).

Therefore, to compose the panel of SSR markers for typing *Sporothrix,* we chose polymorphic motifs of 3, 4, 5, 6, and 10 bp covering the main SSR characterized and distributed among different contigs based on the reference genomes of the isolates 5110 of *S. brasiliensis* and 1099-18 of *S. schenckii*. Altogether, 15 primer pairs were designed in this study and are divided into five multiplexes (Table 2), named M1–M5, according to the scheme shown in Supplementary Figure S1.

### 3.2. In Vitro Application of SSR Markers

A panel of 180 *Sporothrix* isolates was used to evaluate the success rate of marker amplification. High cross-species transferability is reported with excellent performance for all markers for *S. brasiliensis* (94.85–100%), *S. schenckii* (91.84–100%), and *S. globosa* (91.18–100%), with overall amplification success rates ranging from 80.56 to 100%, overcoming an important bottleneck in *Sporothrix* (Supplementary Figure S2). The exception was marker SSR391 (M5), which did not amplify the DNA of *S. globosa* isolates (Supplementary Table S3). In silico analyses revealed five polymorphisms in the SSR391 forward primer (with two mismatches at the 3’ end), preventing their extension in *S. globosa* genomes.

Marker length was determined by capillary electrophoresis. The amplicons’ size and alleles’ identification were performed using GeneMapper, revealing that all markers were polymorphic using the group of 180 isolates. The most polymorphic markers were SSR661 (36 alleles; M4), followed by SSR235 (27 alleles; M1), SSR637 (21 alleles; M1), SSR343 (21 alleles; M3), SSR408 (16 alleles; M1), SSR538 (16 alleles; M2), and SSR11 (16 alleles; M4) while the least polymorphic were SSR150 (7 alleles; M5), SSR50 (9 alleles; M5), and SSR307 (9 alleles; M3). All SSRs presented marked interspecific variation. For example, for *S. brasiliensis*, the greatest diversity was found with the marker SSR661, revealing 31 alleles among 97 isolates. For *S. schenckii,* the greatest diversity was found using the marker SSR637, with 17 alleles among 49 isolates. For *S. globosa*, the highest number of alleles was found with the marker SSR646, with 10 alleles recognized in a group of 34 isolates (Supplementary Tables S3 and S4). The 15 SSR primer pairs amplified 240 alleles from 180 isolates, revealing 151 unique genotypes (Hd = 0.9955) and a mean of 16 alleles per locus. A total of 73 (Hd = 0.985; Simpson Index = 0.0146), 47 (Hd = 0.998; Simpson Index = 0.001701), and 31 (Hd = 0.995; Simpson Index = 0.005348) genotypes were identified in *S. brasiliensis*, *S. schenckii,* and *S. globosa*, respectively.

After identifying the alleles, we calculated the polymorphic statistics for the codominant markers individually (Supplementary Table S4) or considering the M1–M5 multiplexes (Table 3). The *PIC* values showed the excellent ability of each marker to reveal intra and interspecific polymorphisms. Excellent *PIC* values were found for *S. brasiliensis* (*PIC* = 0.8987), *S. schenckii* (*PIC* = 0.9009), and *S. globosa* (*PIC* = 0.8904). The most significant individual *PIC* value was observed for the SSR235 marker (*PIC* = 0.9004), and the lowest was registered for the SSR50 marker (*PIC* = 0.4221), demonstrating good diversity among the studied *Sporothrix* species. Essentially, *S. brasiliensis*, *S. schenckii,* and *S. globosa* displayed comparable levels of polymorphic information content (Table 3). Discriminating power (*D*) was estimated as the likelihood that two random *Sporothrix* exhibit a different pattern of alleles, and all SSR markers showed high overall discriminating power (*D* = 0.7127), notably for emerging pathogens previously considered clonal such as *S. brasiliensis* (*D* = 0.3562) and *S. globosa* (*D* = 0.3075) (Table 3).

The marker index (*MI*) was determined as the product of the effective multiplex ratio (*E*) and the expected mean heterozygosity (*H_avp_*) for polymorphic SSR and was employed to assess the overall utility of each marker system. Equivalent overall *MI* values (*MI* = 0.4878–0.9072) were found for all markers individually (Supplementary Table S3) as well as combined (*MI* = 0.9159) (Table 3).

The expected heterozygosity (*H*), which is the likelihood that an isolate is heterozygous for the locus in the population, was calculated. The expected heterozygosity corresponds to the unbiased genetic diversity of Nei (*HS*). The average expected heterozygosity for the *Sporothrix* species is high (*H* = 0.9159) (Table 3). Considering the earlier report of clonality among members of the clinical clade, the expected high combined heterozygosity for *S. brasiliensis* (*H* = 0.9057) and *S. globosa* (*H* = 0.8993) was remarkable when compared to *S. schenckii* isolates (*H* = 0.9077), corroborating that our SSR panel of markers allows us to explore cryptic diversity in *Sporothrix* of medical relevance.

**Figure 5:**
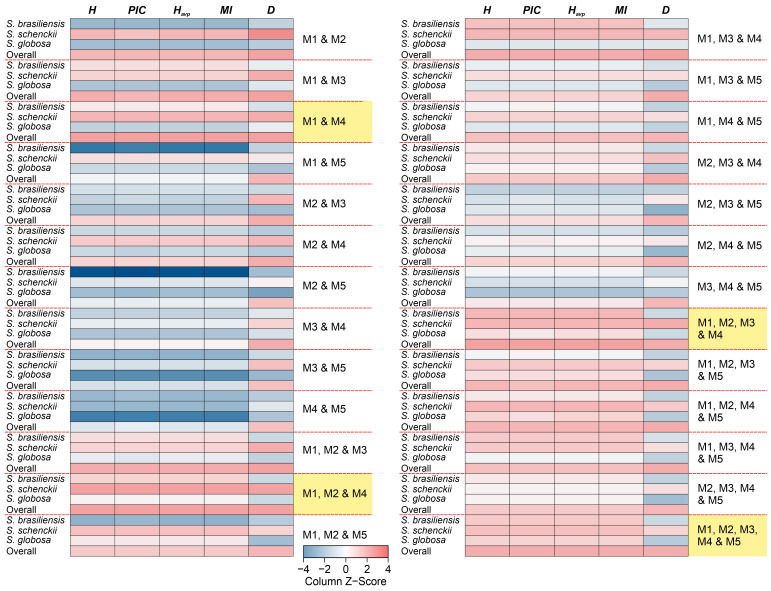
Multiplex combinations were used to assess intra and interspecific diversity in medically relevant *Sporothrix,* revealing that a minimum of three multiplexes (nine SSR markers) are needed to cover considerable diversity in *Sporothrix*. In order of effectiveness, our recommendations are (1) M1, M2, M3, M4, and M5; (2) M1, M2, M3, and M4; (3) M1, M2, and M4; and (4) M1 and M4.

Combining SSR markers significantly increases intra and interspecific diversity (Table 3). To evaluate which markers are more informative and consequently more efficient in genotyping *Sporothrix*, we performed a simple permutation of multiplex panels and evaluated the performance of each scenario, which is presented as a heatmap in Figure 5. The combinations involving the sets M1, M2, and M4 show excellent performance; for example, the highest values of *PIC* and *H* were found for combined M1 and M4 (*PIC* = 0.9156 and *H* = 0.9208), M1, M2, and M4 (*PIC* = 0.9171 and *H* = 0.9222), M1, M2, M3, and M4 (*PIC* = 0.9155 and *H* = 0.9206), and M1, M2, M3, M4, and M5 (*PIC* = 0.9101 and *H* = 0.9159).

**Figure 6:**
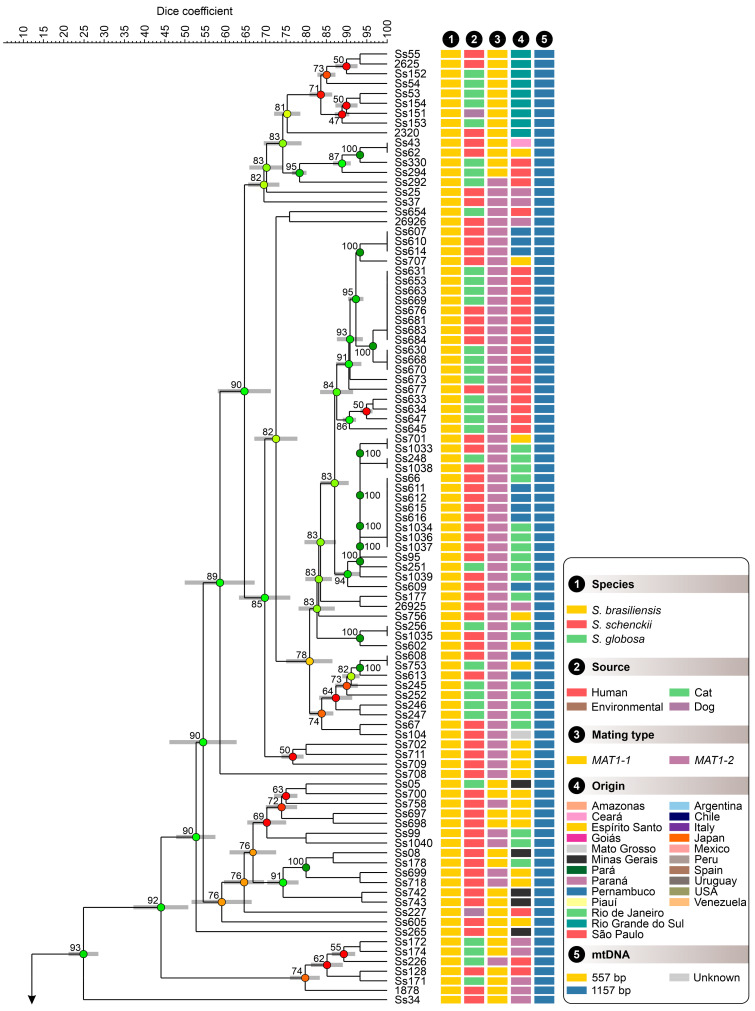

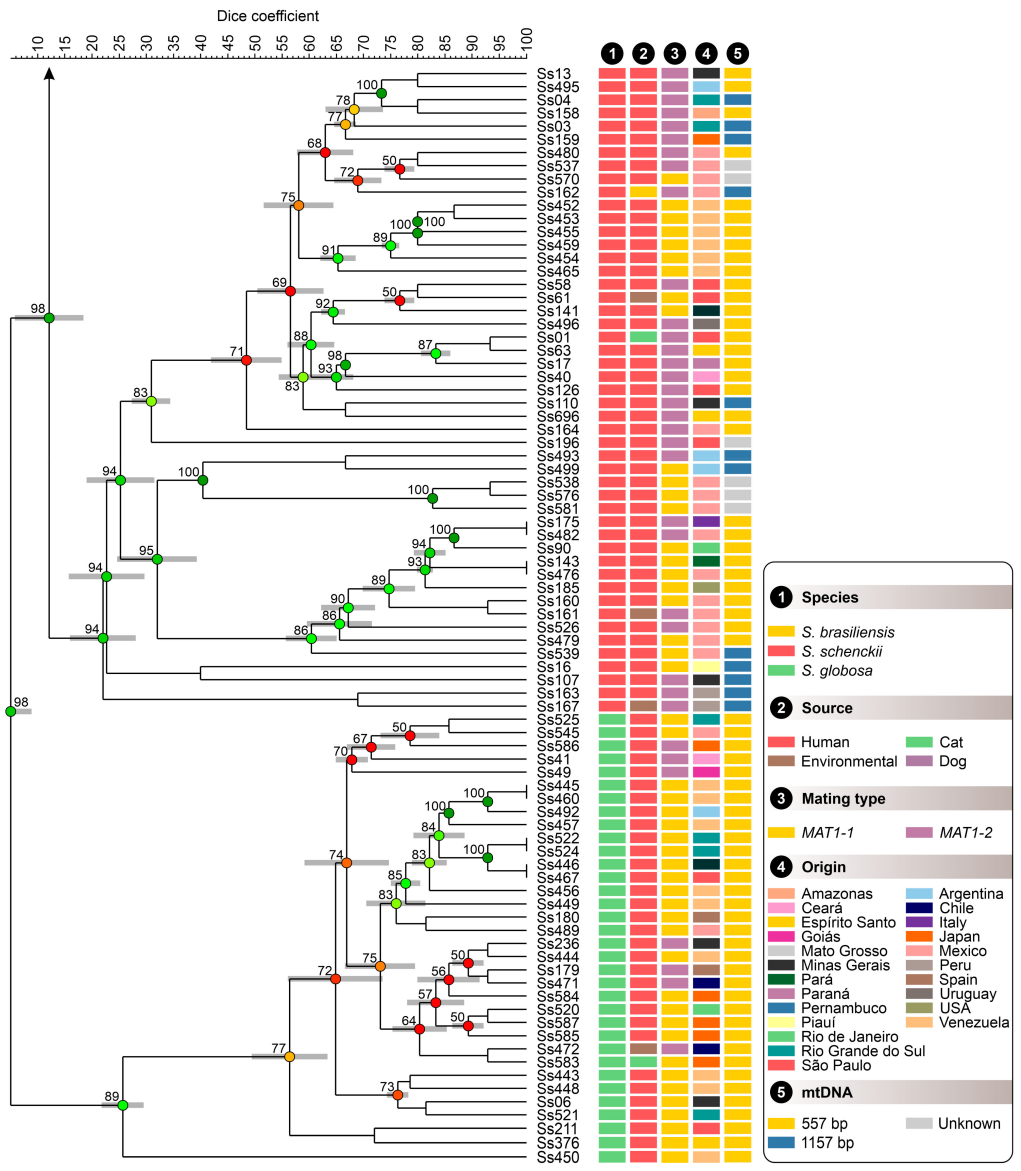
The UPGMA dendrogram, based on SSR markers, generated with a total of 15 SSR markers for 180 *Sporothrix* isolates originated worldwide. The dendrogram shows cophenetic correlation values (circles are represented by color ranges between green-yellow-orange-red according to decreasing cophenetic correlation) for a given clade and its standard deviation (grey bar). For pairwise genetic distances calculation, the Dice similarity coefficient was used. The cophenetic correlation of the dendrogram is 98%. Further information about isolate sources can be found in Supplementary Table S1.

The UPGMA-based clustering method was used to generate the dendrogram shown in Figure 6. The cluster evaluation reveals three well-supported clades with a significant global cophenetic correlation coefficient (98%) for *S. brasiliensis* (93%; Dice similarity = 24.91% ± 3.30%), *S. schenckii* (94%; Dice similarity = 22.02% ± 5.70%), and *S. globosa* (89%; Dice similarity = 25.69% ± 3.48%). Employing an arbitrary cutoff of 60 ± 2%, a few subgroups are recognized in the dendrograms (Supplementary Figure S3). In *S. brasiliensis,* we highlight the clade Ia (n = 16, Dice similarity = 69.51% ± 3.51%) composed mainly of isolates from Rio Grande do Sul, harboring the *MAT1-1* locus, and the clades Ib (n = 57, Dice similarity = 69.74% ± 5.99%), Ic (n = 15, Dice similarity = 59.09% ± 7.07%), and Id (n = 6, Dice similarity = 79.72% ± 3.35%) composed mostly of isolates from the Southeast region, exhibiting the *MAT1-2* locus. Isolates from humans and animals in Rio de Janeiro were hitchhiking through clades Ib-Id (Supplementary Figure S3).

Clustering patterns in *S. schenckii* follow a geographic trend as observed for clades IIa (n = 16, Dice similarity = 58.07% ± 6.05%), IIb (n = 11, Dice similarity = 58.90% ± 4.13%), IIc (n = 5, Dice similarity = 40.46% ± 0.37%), and IId (n = 11, Dice similarity = 60.40% ± 4.31%). Most clades (IIa–IIc) are composed of isolates recovered in Latin America, while clade IId harbors global specimens represented by Brazil, USA, Italy, and Mexico. In *S. globosa*, four clades with limited diversity were observed being named IIIa (n = 5, Dice similarity = 67.86% ± 2.59%), IIIb (n = 12, Dice similarity = 73.09% ± 6.09%), IIIc (n = 10, Dice similarity = 76.32% ± 1.59%), and IIId (n = 4, Dice similarity = 76.32% ± 1.59%). We highlight clade IIIc with closely related samples from Brazil, Chile, Spain, Venezuela, and Japan (Supplementary Figure S3). Dice similarity coefficient values for the SSR markers indicated that diversity is high for the *Sporothrix* genotypes (Figure 6). The observed clustering patterns indicate that *S. brasiliensis* and *S. schenckii* are sister species, and *S. globosa* assumes a more basal position within the clinical clade.

**Figure 7:**
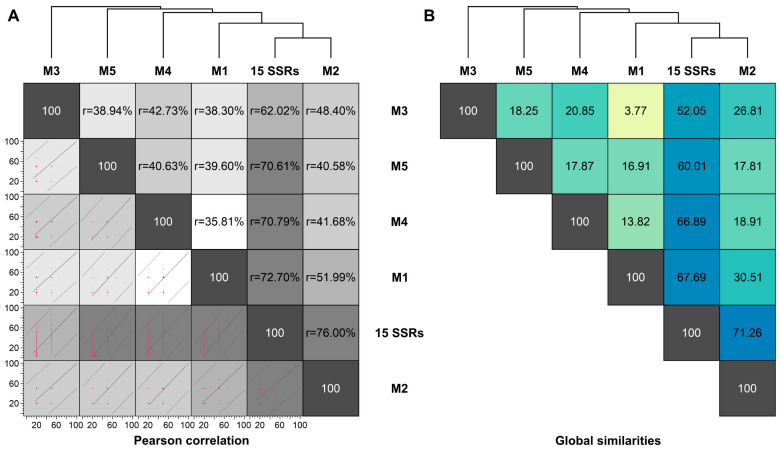
Pairwise congruence between experiments (multiplexes): (**A**) Pearson correlation between experiments evaluated for 180 *Sporothrix* isolates is displayed above the diagonal, and correlation plots are depicted below the diagonal. (**B**) A similarity index was calculated in BioNumerics v7.6, comparing M1–M5 and the complete panel.

We used Pearson correlation and the global similarity index to assess the topological congruence between the results obtained for the five multiplexes and the complete panel with the 15 SSR markers. Pairwise comparisons revealed a similar and consistent clustering pattern, as evidenced by the positive Pearson correlation (Figure 7). Pearson values and global similarity are concordant and are more significant for pairwise comparisons between the whole panel (15 SSRs) and multiplexes M2 (r = 76%), M1 (r = 72.70%), M4 (r = 70.79%), and M5 (r = 70.61%) (Figure 7). Therefore, the dendrograms generated from the SSR marker were more congruent than expected by chance, revealing consistent diversity patterns. Moreover, the observed 100% agreement between microsatellite data and molecular identification also supports using SSR markers to identify *Sporothrix* down to the species level.

### 3.3. Population Structure Based on Multivariate Cluster Analysis

**Figure 8:**
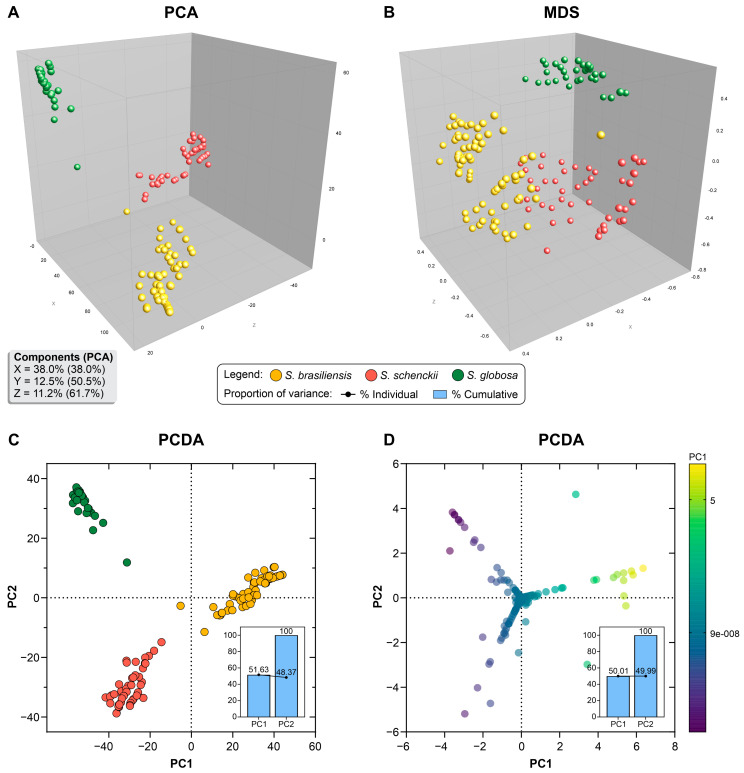
Dimensionality reduction using principal component analysis (PCA), multidimensional scaling (MDS), and principal component discriminant analysis (PCDA) of the SSR markers: (**A**) PCA of 180 *Sporothrix* isolates shows that the first three components explain 61.7%; (**B**) MDS analysis of 180 *Sporothrix* isolates; (**C**) PCDA analysis of 180 *Sporothrix* isolates splits *S. brasiliensis*, *S. schenckii,* and *S. globosa* into three different clusters; (**D**) PCDA analysis of SSR alleles reveals the best discriminating components for *Sporothrix* groups. Dimensionality reduction analyses were created in the software BioNumerics v7.6.

SSR markers were used to generate band-matching tables, which were then subjected to a multivariate cluster analysis using BioNumerics v7.6. In the PCA and MDS plots, the scattering of 180 *Sporothrix* isolates among the three coordinates demonstrated a species-specific tendency to clustering corresponding to different *Sporothrix* species. The set of SSR showed an excellent cumulative explained percentage, with 61.7% of the variation described by the first three components (X, Y, and Z coordinates, Figure 8A), signaling a solid genetic structure. The dimensionality reduction method showed a significant level of intraspecific dispersion (diversity) and a substantial genetic distance between any two individuals of different species (interspecific diversity) (Figure 8B). Discrimination (DA) on PCA supports splitting *S. brasiliensis*, *S. schenckii,* and *S. globosa* using SSR markers (Figure 8C). The first component (PC1 = 51.63%) segregates *S. brasiliensis* from the remaining species, while PC2 (48.37%) differentiates *S. schenckii* and *S. globosa* clusters. PCDA analysis using the characters entries (240 alleles) describes a scenario of species-specific alleles whose sample projections along the axes significantly contribute to interspecific discrimination (Figure 8D).

**Figure 9:**
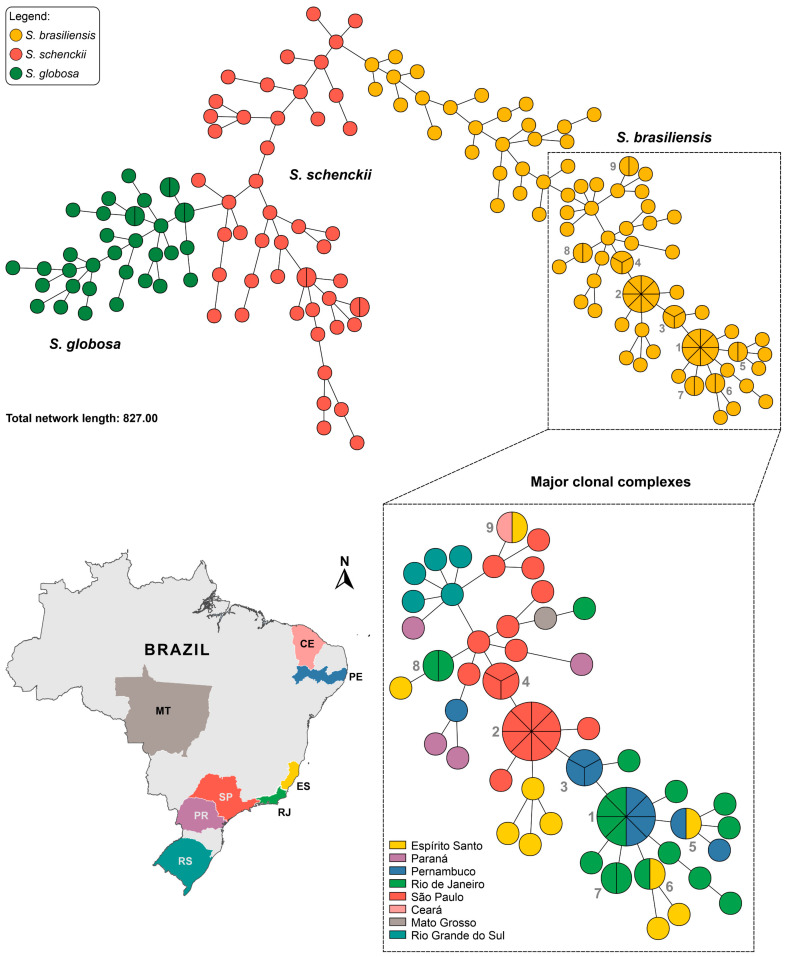
Minimum Spanning Trees (MSTs) showing the genetic relationship among 180 *Sporothrix* isolates using 15 SSRs markers. MST was created in the software BioNumerics v7.6. The annotated figure with all isolates is shown in Supplementary Figure S4.

The MSTs derived from the SSR markers in Figure 9 effectively confirm the genetic structure observed using dimensionality reduction methods, with most isolates assuming unique genotypes. Using SSR markers, we recognized the emergence of nine clonal complexes in *S. brasiliensis*, with isolates with identical genotypes and distinct geographic origins, thus indicating transmissibility, or in the cases of isolates with identical genotypes recovered from humans and animals, confirming zoonotic transmission. Therefore, MST can be used to understand cat-transmitted *S. brasiliensis* during geographical expansion. The *S. brasiliensis* clonal complex #1 is formed by eight isolates (four from Rio de Janeiro and four from Pernambuco). These data suggest the migration of *S. brasiliensis* from Rio de Janeiro to Pernambuco, most likely via an introduction of a diseased animal from endemic to non-endemic areas. Other complexes refer to isolates from São Paulo (see complexes 2 and 4, Figure 9). The main characteristic of clonal complexes 2 and 4 reflects a temporal variable since these isolates were recovered in the same area in a short period.

The presence of clonal complexes is inherent to the founder effect and depicts the dynamics of the expansion of *S. brasiliensis*, where initially, after the introduction of a genotype, it spreads rapidly through scratches and bites of infected or colonized cats to other animals and humans (Figure 9). Therefore, time and space influence the recognition of the emergence of clonal complexes during cat-transmitted sporotrichosis outbreaks. As it is a dynamic process, multiple introductions over time lead to greater genotypes diversification that strengthens the population structure (see genotypes referring to São Paulo in the insert of Figure 9).

**Figure 10:**
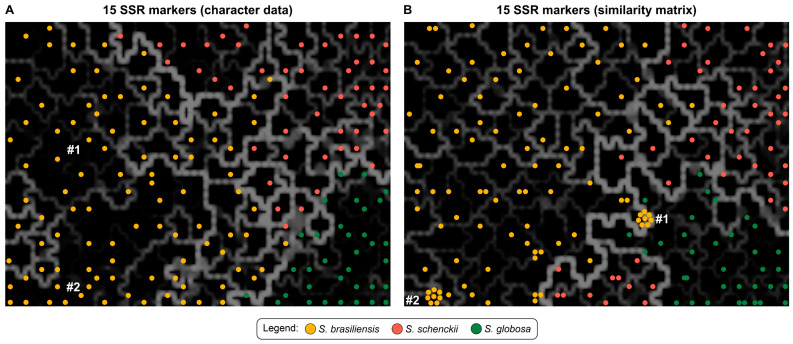
Self-organizing map representing the distribution of 180 *Sporothrix* isolates genotyped using SSR markers. Kohonen maps using (**A**) character data or (**B**) the similarity matrix. The regions occupied by clonal complexes #1 (Rio de Janeiro and Pernambuco: Ss66, Ss611, Ss612, Ss615, Ss616, Ss1034, Ss1036, and Ss1037) and #2 (São Paulo: Ss631, Ss653, Ss663, Ss669, Ss676, Ss681, Ss683, and Ss684) are indicated. The dimensioning analyses were performed using BioNumerics v7.6 to determine the consistency of the differentiation of the populations defined by the cluster analysis.

The allelic data were used to generate self-organizing maps employing an artificial neural network. Kohonen maps using the characters or the similarity matrix are shown in Figure 10 for 180 color-coded isolates enclosed in cells with a black background. Isolates positioned in adjacent cells are separated by lines with different thicknesses and colors, varying between black as the lowest intensity and white as the highest intensity. The more intense (white) and thicker the line separating isolates allocated to adjacent cells, the more significant the accumulated genetic differences between the isolates. On the other hand, the thinner and darker (black) the line separating the isolates, the less powerful the genetic differences that separate these isolates. Therefore, it is evident that interspecific differences are meaningful among *S. brasiliensis*, *S. schenckii,* and *S. globosa,* according to Kohonen’s maps (Figure 10).

The interpretation of self-organizing maps suggests that intraspecific diversity in *S. globosa* is relatively low given the negligible lines depicted, a situation contrary to *S. schenckii,* where many isolates accumulated differences that translate into thicker and more intense segregating lines. In *S. brasiliensis,* we find a hybrid scenario, underlining specimens with little differentiation, separated by tenuous and dark lines, and genetically divergent isolates, whose boundary lines present expressive thickness colored with a more vivid white. Interestingly, clonal complexes #1 (Rio de Janeiro and Pernambuco: Ss66, Ss611, Ss612, Ss615, Ss616, Ss1034, Ss1036, and Ss1037) and #2 (São Paulo: Ss631, Ss653, Ss663, Ss669, Ss676, Ss681, Ss683, and Ss684) of *S. brasiliensis* recognized in the dendrogram (Figure 6) and MSTs (Figure 9), were placed in the same cell in the Kohonen’s maps (Figure 10 B), confirming the limited diversity among these samples. The SOMs, therefore, allowed us to understand the dynamics of differentiation of *Sporothrix* populations during outbreaks and epidemics (Figure 10).

### 3.4. Population Genetics in Sporothrix Species

**Figure 11:**
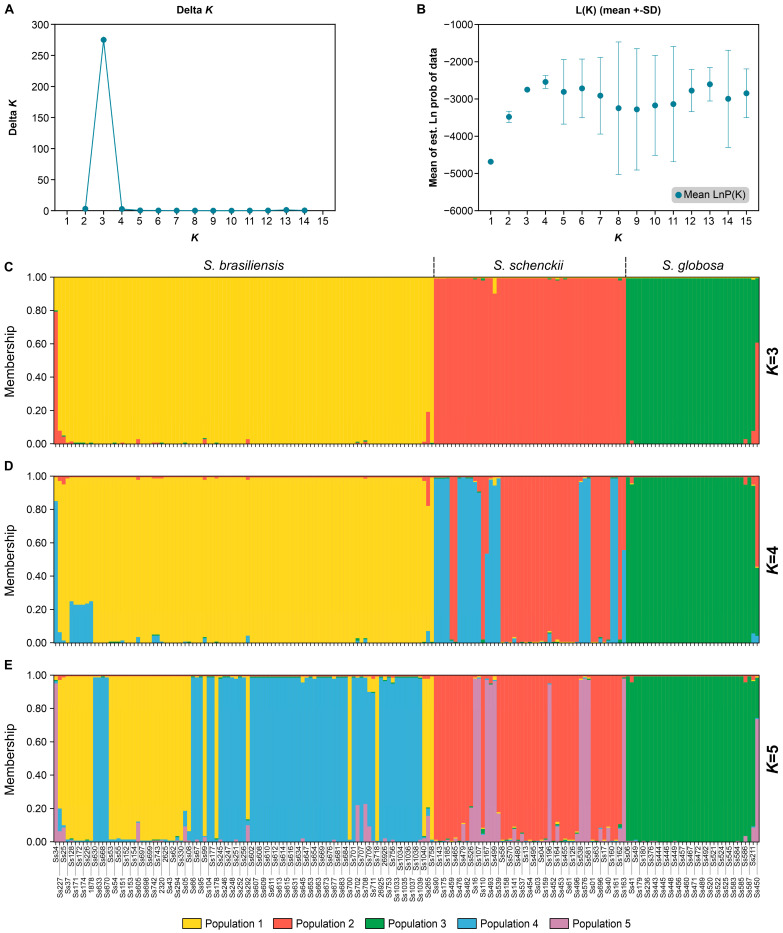
Population structure of medically relevant *Sporothrix* isolates: (**A**,**B**) StructureHARVESTER results show the most reasonable number of genetic clusters (*K*) within the complete data according to the approach described by Evanno et al. [68]. The population genetic structure of the estimated ΔK value (275.005058) defined the maximum value at *K* = 3 and LnP (Mean LnP(K) = −2749.95 ± 1.89). Bayesian cluster analyses with Structure for (**C**) *K* = 3, (**D**) *K* = 4, (**E**) *K* = 5 based on SSR markers. Each vertical bar exemplifies one individual and its probability of being allocated to clusters. Further information about inferred ancestry can be found in Supplementary Table S5.

Subsequently, we investigated the genetic structure of *Sporothrix* populations by evaluating allele frequencies between groups. Structure analysis of 180 isolates determined that three groups best described the data (Figure 11A,B). Thus, considering *K* = 3, the genotypes of *S. brasiliensis* (cluster 1), *S. schenckii* (cluster 2), and *S. globosa* (cluster 3) tend to have high proportions of species-specific association. A few genotypes of *S. brasiliensis* (cluster 1, e.g., Ss34 and Ss265) and *S. globosa* (cluster 3, e.g., Ss211 and Ss450) shared ancestry with genotypes of *S. schenckii* (cluster 2) and therefore are mixed in the three-clusters model (Figure 11C, Supplementary Table S5). These assumptions are consistent with the multivariate analysis of clusters that detected the presence of outliers in our panel of isolates. Considering *K* = 4 or 5, the *S. brasiliensis* isolates are partitioned into two major groups: the Rio de Janeiro clade and the Rio Grande do Sul clade. For *S. schenckii,* this subdivision considers isolates from South America and globally distributed. The cluster of *S. globosa* remained homogeneous at *K* = 4 or 5 (Figure 11D,E).

**Figure 12:**
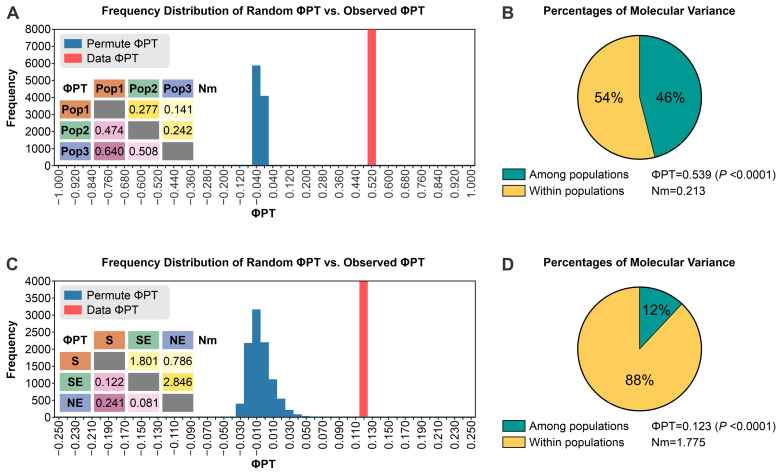
ΦPT genetic distances among medically relevant *Sporothrix* species based on 15 SSR markers. (**A**,**C**): Frequency Distribution of Random ΦPT versus Observed ΦPT. (**B**,**D**): Percentages of molecular variance. (**A**,**B**): Group *Sporothrix* (n = 180); Pop1 is 97 *S. brasiliensis* isolates; Pop2 is 49 *S. schenckii* isolates; Pop 3 is 34 *S. globosa isolates*. (**C**,**D**): Group *Sporothrix brasiliensis* (n = 96). The most significant ΦPT values were found between the South (S) and Northeast (NE) *S. brasiliensis* isolates (ΦPT = 0.241), along with the lowest Nm values (0.786). The lowest values were found in pairwise comparisons between the Southeast and Northeast, demonstrating the proximity of these isolates (ΦPT = 0.081) and suggesting that the migration occurred between members of the southeastern region and the northeastern region of Brazil. S: *S. brasiliensis* South isolates (n = 18); SE: *S. brasiliensis* Southeast isolates (n = 67); NE: *S. brasiliensis* Northeast isolates (n = 11). Isolate Ss104 from Central-West Brazil was removed from the *S. brasiliensis* dataset. Nm is gene flow.

The AMOVA for the 180 individuals allowed a partition of the global variation into two levels (Table 4). The genetic differences between the three populations were significant (ΦPT = 0.539; Nm = 0.213; *p* < 0.0001). The proportion of variation attributable to differences within (46%) and among populations (54%) was somewhat equivalent (Figure 12B).

Pairwise ΦPT and gene flow (Nm) values between populations 1 and 2 (ΦPT = 0.474, Nm = 0.277, *p* < 0.0001), 1 and 3 (ΦPT = 0.640, Nm = 0.141, *p* < 0.0001), and 2 and 3 (ΦPT = 0.508, Nm = 0.242, *p* < 0.0001) and their significance are shown in Figure 12A. Subsequently, *S. brasiliensis* isolates were segregated according to the geographic origin considering South (n = 18), Southeast (n = 67), and Northeast isolates (n = 11). The AMOVA of the distance matrix for the 96 individuals of *S. brasiliensis* allowed a partition of the global variation in two levels (Table 4). The proportion of variation attributable to differences within populations (88%) was high, while only 12% occurred among subpopulations (Figure 12D). Likewise, estimated ΦPT and Nm values between *S. brasiliensis* in the South and Southeast regions (ΦPT = 0.122, Nm = 1.801, *p* < 0.0001), South and Northeast (ΦPT = 0.241, Nm = 0.786, *p* < 0.0001), and Southeast and Northeast (ΦPT = 0.081, Nm = 2.846, *p* < 0.0001) indicate that the Southeast and Northeast populations are the most similar to each other (low differentiation and high gene flow). The South population differed most from the remaining areas (Figure 12C). Notably, gene flow between *S. brasiliensis* populations in the Southeast and Northeast regions was 3.62 times higher than that between the South and Northeast regions (*p* < 0.0001), supporting the introduction of isolates from the Rio de Janeiro clade to the Northeast region, which is also supported by the temporal variability of the evolution of the outbreaks (Figure 12).

**Figure 13:**
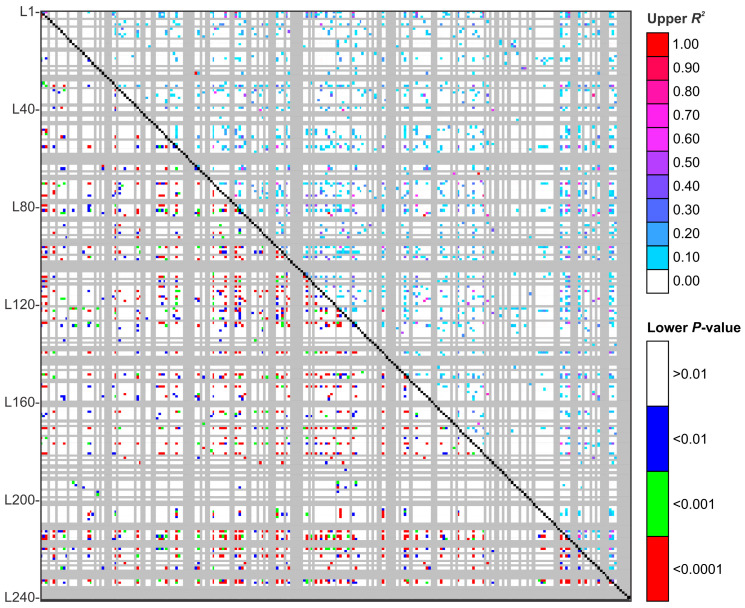
Marker-marker disequilibrium matrix for 240 polymorphic loci based on medically relevant *Sporothrix* species (*S. brasiliensis*, n = 97; *S. schenckii*, n = 49; *S. globosa*, n = 34). Pairwise calculations (28,560 pairs) of linkage disequilibrium (*LD*) (r^2^) are displayed above the diagonal, with the corresponding *p*-values for Fisher’s exact test shown below the diagonal. The locus position (L) is indicated on the left X-axis. The color legends indicate the level of significance (*p*-value) and the corresponding strength of *LD* (r^2^). Mean r^2^ = 0.02714. Mean *D′* = 0.8910.

In a randomly mating (or panmictic) population, allele frequencies are projected to be approximately similar between groups. Nevertheless, mating tends to be nonrandom to some extent, triggering the structure to emerge. Thus, we analyzed the linkage disequilibrium (*LD*) through the nonrandom association of alleles at two or more loci in the genome using Fisher’s exact test (Figure 13). As expected, only 1201 (~4.20%) possible SSR marker pairs were in *LD* at *p* < 0.001, demonstrating that the level of *LD* continued low in the *Sporothrix* isolates incorporated in our analysis (Table 5).

Splitting *Sporothrix* to species level has eliminated some of the observed *LD* degrees; although; the remaining equilibrium patterns continue to be similar. In *S. brasiliensis* (n = 97), a total of 161 of 28,560 (0.56%) genome marker pairs were in LD at *p* < 0.001, and the strongest *LD* (r^2^ = 1) was observed for only 2 pairs of markers (0.007%). In *S. schenckii* (n = 49), a total of 161 of 28,560 (0.56%) genome marker pairs were in *LD* at *p* < 0.001, and the strongest *LD* (r^2^ = 1) was observed for 8 pairs of markers (0.028%). In *S. globosa* (n = 34), a total of 17 of 28,560 (0.059%) genome marker pairs were in *LD* at *p* < 0.001, and the strongest *LD* (r^2^ = 1) was not detected (Table 5).

## 4. Discussion

Microsatellites or simple sequence repeats of DNA present significant polymorphism due to the high mutation rates at these loci. These elements are widely distributed in *Sporothrix* genomes and can play a vital role in the organization, recombination, gene regulation, quantitative genetic variation, and gene evolution, in addition to serving as relevant markers for dissecting both population structure and analysis of genetic diversity [77]. The availability of complete genomic sequences from various *Sporothrix* offers the unique opportunity to characterize and compare SSR distributions [78]. The distribution and frequency of SSRs underlined repeat unit lengths of 2–10 bp in the *Sporothrix* genome revealed a total of 118,140–143,912 SSR loci (82,841–98,369 unique markers), with a density of 3651.55–3804.65 SSR/Mb. The overall SSR density in *S. brasiliensis* (3803.12 SSR/Mb) was slightly higher than the one observed for *S. schenckii* (3677.32 SSR/Mb) and *S. globosa* (3653.11 SSR/Mb) but roughly similar to the mid-pathogenic *S. pallida* (3804.65 SSR/Mb), despite the smaller genome of *S. brasiliensis* (coefficient of variation = 1.719%).

It is often accepted that larger genomes harbor more SSRs than smaller ones; although, there is no clear association between SSR content and genome size [79,80,81]. This statement does not seem proper for fungi, as several fungal species have varying SSR frequencies and abundances in their genomes, despite having equivalent genome sizes [82]. According to Morgante et al. [83], in plants such as *Arabidopsis thaliana*, the overall frequency of microsatellites is inversely related to genome size and the proportion of repetitive DNA. The results found in *Sporothrix* contrast with this general tendency, as we reveal a positive correlation between the size of the genomes and the density of SSR, as well as the distribution of these elements by the length of the contigs of the analyzed isolates.

Microsatellite composition and frequency confirmed that di- and trinucleotides, the most extensively studied motifs, were the most abundant SSRs, accounting for ~95.41% of all SSRs identified in *S. brasiliensis*. This phenomenon seems to be supported by the negative selection pressure driven by polymerase slippage during DNA replication [84] or other enzymes involved in various aspects of DNA processing [85]. The SSR frequency decreased as the complexity and number of repeat units increased. For *Sporothrix* genomes, the nucleotide composition of an SSR motif was strongly biased towards C or G, especially at the first base. For example, the SSR motif with the highest frequency of repeats was GC/CG, making up 17.01% (coefficient of variation = 13.13%) of all dinucleotide loci in the genome. Furthermore, a similar trend was also observed for longer motifs, such as CAG/TGC/GGC, which were the most abundant trinucleotide motifs. These data were already expected, considering that *S. brasiliensis* and *S. schenckii* harbor GC-rich sequences of ~62% of their genome content, and in *S. globosa* and *S. pallida* this content is approximately 52–54% [37,38,40,41].

We successfully designed primers for 98.16–99.58% of the microsatellites documented. However, our selection criteria considered only (i) polymorphic markers covering the primary motifs characterized in silico and distributed over different contigs, (ii) that presented a high potential for cross-species transferability (i.e., allowed portability between species), and (iii) with excellent multiplexing capability. Therefore, our panel comprises 15 SSR markers suitable for genotyping *S. brasiliensis*, *S. schenckii*, and *S. globosa*. As a proof of concept, it has been successfully applied to genotype 180 isolates with a positive amplification rate of >90%, allowing the recognition of 240 alleles in a systematic population genetic study. The number of alleles per locus ranged from 7 to 36 with a mean of 16, with a moderate to high *PIC* (0.4221–0.9004; mean = 0.6802), which confirms the success of our panel of highly informative genetic markers [59]. The performance of our assay was superior to the results found by Gong et al. for *S. globosa,* who described a panel of 10 SSR markers with an average of 5.50 alleles (range 3–13) showing a moderate *PIC* (0.441) [36]. Therefore, our results indicate that the genetic variability in *Sporothrix* was estimated with the aid of highly polymorphic markers. 

In a scenario where the individual SSR markers showed excellent discriminatory power, when combined, they revealed unique genetic profiles of alleles for the analyzed *Sporothrix*. Therefore, we investigated the impact of different permutations of markers on estimates of genetic diversity. We aimed to optimize the genotyping process, accelerating the bench steps, reducing genotyping costs, and ensuring equivalent efficiencies [86]. Our results point to a minimal scenario that includes at least three multiplexes, M1, M2, and M4, followed by the addition of M3, and M5 in order of efficiency (an essential criterion in scenarios with limited budgets). The most promising strategy for estimating genetic diversity encompasses the five multiplexes (M1–M5).

UPGMA dendrograms produced grouping profiles compatible with the evolutionary history of *Sporothrix* species using DNA sequencing methods; i.e., *S. brasiliensis* and *S. schenckii* are sister species, closely related, and this group is close to *S. globosa* [22,43]. The dendrogram recognized small clonal complexes in all species, with isolates showing 100% similarity (Dice) between the generated profiles. Such a scenario repeatedly appears in the literature, confirmed by multilocus analysis of genes encoding proteins and barcoding markers, AFLP analyses, and phylogenomic analyses [5,6,7,23,25,29,32,37,43,87,88,89,90]. Congruent genotyping methods appear in *Sporothrix* and other fungi of medical relevance, such as *Histoplasma* [91], *Paracoccidioides* [58], *Fonsecaea pedrosoi* [92], *Fusarium* [93], or *Candida auris* [94]. Therefore, we emphasize that the extensive in silico mining of SSRs was vital to ensure the effectiveness of these markers, avoiding, for example, the choice of monomorphic SSRs or those with low transferability potential.

The genetic diversity in emerging *Sporothrix* species has been debated in recent years with the purpose of determining to what extent the population structure in *Sporothrix* is constituted of clonal or recombinant individuals. The population genetic variables depend on time, space, and genetic markers, and one must consider: (i) the species and number of isolates evaluated; (ii) the timescale covered by the data (i.e., sampling only at the beginning of an outbreak or multiple collections over time); (iii) sampling spatial diversity, considering specimens from endemic and non-endemic areas; and (iv) the discriminating power of the evaluated marker (e.g., DNA sequencing versus fingerprint markers such as AFLP and SSRs, or SNPs panels derived from whole-genome sequencing). That said, it is evident that the initial studies on the molecular epidemiology of sporotrichosis experienced the effect of genetic markers masking diversity, such as the sequencing of calmodulin, beta-tubulin, and ITS region [7,23,25,26,28,29,43,89,95,96]. With the introduction of polymorphic markers such as AFLPs [34,35], we revealed a highly diverse scenario in *S. brasiliensis*, which is also confirmed here using SSR markers. However, although AFLPs and microsatellite markers show excellent discrimination power, we also detected clonal complexes during the initial stage of outbreaks involving epizooties (cat–cat transmission) or zoonoses (cat–human transmission) [9,87]. Our sample group represented such complexes in Rio de Janeiro, Pernambuco, and São Paulo.

De Carvalho et al. [35,78] describe that introducing a sick animal in an area free of sporotrichosis leads to the development of the founder effect. This scenario was also confirmed using SSR markers, characterized by the reduction in genetic variability resulting from introducing a small group of individuals (founders) separated from the larger parental population. As there are no sanitary barriers to contain outbreaks, humans constantly move between endemic and non-endemic areas carrying symptomatic and asymptomatic animals [97,98,99,100]. Brazil has two epicenters of cat-transmitted sporotrichosis with particular genetic features. The oldest outbreak of feline sporotrichosis began in Rio de Janeiro in the mid-1990s [101,102] and is typified by individuals harboring the *MAT1-2* locus [74,103]. Molecular markers can track the dispersion of *S. brasiliensis* from the parental population in Rio de Janeiro across border states such as São Paulo [104,105,106], Minas Gerais [7,25], and Espírito Santo [107,108]. The second relevant genetic group of *S. brasiliensis* has its epicenter in Rio Grande do Sul and comprises *MAT1-1* idiomorphs [7,74,103,109,110]. Our data from SSRs confirm a more significant contribution of genotypes circulating in Rio de Janeiro to outbreaks occurring in other Brazilian states. This trend is even more evident by the low allelic donation of individuals circulating in Rio Grande do Sul (limited gene flow), and the absence of Rio Grande do Sul isolates hitchhiking through clades identified in this study (e.g., Ib-Id) [111].

Multiple genotypes of *S. brasiliensis* circulate in Brazil’s South/Southeast axis, as demonstrated by genetic diversity and population structure. The most parsimonious scenario to explain the dispersion dynamics for the observed genetic patterns suggests unidirectional migration from the Southeast to the Northeast of Brazil. This hypothesis is supported by (i) the temporal evolution of the outbreaks since there are rare reports of cat-transmitted sporotrichosis in the northeast region until the mid-2015s [12,25]; (ii) skewed distribution of *MAT* idiomorphs with an overwhelming prevalence of *MAT1-2* isolates in Pernambuco [35]; (iii) the high gene flow between isolates from the Rio de Janeiro clade and specimens from Pernambuco, and (iv) low differentiation values (ΦPT). The founder effect probably played a vital role in this dynamic, as the introduction was recent, and the diversity of isolates in the Northeast was low compared to the parental population in Rio de Janeiro. Some rare *S. brasiliensis* isolates were detected in the Northeast region in mid-1997 (e.g., Ss43) [4,25,87]. Although the Ss43 isolate was part of our sample group, it was unrelated to the recent cat-transmitted outbreaks taking place in Pernambuco. On the contrary, a major clonal complex (Rio de Janeiro/Pernambuco) was described in this study in agreement with previous AFLP data [35].

We used the clustering method described by Pritchard et al. [67] to infer population structure in *Sporothrix* and assign individuals to genetic populations. We assume a model in which there are *K* populations, each characterized by a set of allele frequencies at each SSR locus [112]. *Sporothrix* were then probabilistically assigned to three populations according to the method described by Evanno [68]. For some individuals, in the three species evaluated, we observed mutual attribution to two or more populations, indicating patterns of introgression (miscegenation) or incomplete lineage sorting, a scenario that had already been explored using AFLP markers [35] and gained new support using codominant markers. Putative hybrids are highlighted in *S. brasiliensis* (e.g., Ss34, Ss128, and Ss265) and *S. globosa* (e.g., Ss211, Ss376, and Ss450), always with the contribution of alleles from *S. schenckii* population.

Interestingly, when estimating *K* = 4 or 5, we observed the bipartition of the *S. brasiliensis* and *S. schenckii* isolates into two clusters each, but not among *S. globosa* isolates, which maintained a homogeneous population structure. Interestingly, the subdivision of the *S. brasiliensis* group reveals isolates belonging to the Rio de Janeiro clade or the Rio Grande do Sul clade. The partition of *S. schenckii* shows interesting geographical relationships, with isolates from Latin America and isolates representing a global population (e.g., Brazil, USA, Italy, and Mexico). Generally, this *S. schenckii* partition is related to the genotypes identified by mitochondrial DNA in the classic studies conducted by Ishizaki et al. [30,75,113,114,115,116,117,118,119,120,121]. Recently, *S. globosa* from China was separated into eight distinct groups using AFLP markers [122] and three genetic clusters using a panel of ten microsatellites [36]. However, such partition has no significant association with clinical manifestations. *Sporothrix globosa* displays low genetic diversity when investigated using AFLP markers in India [123]. Similar to the Chinese study, there is no correlation between genotypes and clinical presentation or geographic distribution [123]. The absence of a phylogeographic structure is commonly observed in outbreak scenarios where numerous variants can be discovered before becoming extinct [124]. However, we argue that such inference may also result from the markers’ resolution power and statistical models used. The scenario observed in *S. globosa* [6] may be similar to that observed in populations previously characterized as panmictic in *Aspergillus fumigatus*. In *Aspergillus*, earlier studies have shown little or no correlation between population structure with geography, as genetically identical (clonal) isolates are collected from different locations worldwide [125,126,127]. However, Lofgren et al. [128] used pan-genomic analyses to explore the panmixia model uncovering strong evidence for three primary populations of *A. fumigatus* (Clades 1–3), with recombination occurring only rarely between populations and often within them.

In population genetics, linkage disequilibrium (*LD*) in small genomic areas reflects the history of natural selection, genetic conversion, mutation, and other mechanisms that affect evolution. At the same time, genome-wide *LD* plays a more significant role in informing about past events of pathogen evolution, population, the reproductive system, and the pattern of geographic subdivision [66,129]. Therefore, *LD* is a powerful indicator of the population genetic forces that structure a genome, and here we employ the nonrandom association of alleles at different loci to investigate whether *Sporothrix* populations are mostly clonal or recombinant. In a population with random mating (panmictic) and without selection, the probability of a given allele participating in the reproductive process is inherent only to its population frequency, with all individuals being potential partners; that is, there are no mating restrictions [130]. In contrast, failure or lack of mating results in populations with significant *LD* [131,132,133,134,135]. Therefore, the low marker-marker *LD* levels found for *Sporothrix* suggest expressive recombination estimates (mean r^2^ = 0.02–0.05), supporting that alleles can freely recombine into new genotypes during the mating process [136], a scenario previously reported using AFLP markers [78] and mating-type distributions [74,103,137,138]. High genetic diversity scenarios with low *LD* values, suggestive of significant sexual recombination, have been observed in *Ophiostoma montium* [139], *Fusarium graminearum* [140], *Zymoseptoria tritici* [141], *Glomus claroideum* [142] and are associated with the ability to quickly respond to new environmental conditions or changes in the host. This scenario would be a force driving the emergence of *Sporothrix* species. 

## 5. Conclusions

Our capacity to gain insights into the sporotrichosis transmission chain impacts our ability for decision making to mitigate disease progression. Sporotrichosis is a neglected mycosis, lacking robust investments in basic and applied research. Here, we develop genetic markers with high discriminatory value to shed light on the molecular epidemiology of human and animal sporotrichosis, overcoming the two critical bottlenecks mentioned above: (i) our approach highlights microsatellite markers as a powerful and inexpensive tool tremendously relevant in low-budget scenarios and (ii) we optimized an SSR panel with high amplification success rates within and between *Sporothrix* species, allowing us to test epidemiological and evolutionary hypotheses comparatively. 

A weak signature of mixed ancestry was detected, which may clarify the abrupt appearance of *Sporothrix* with increased virulence characteristics or producing severe, atypical, and refractory cases of sporotrichosis. The center of origin of cat-transmitted sporotrichosis may be used to gain insight into the *Sporothrix* capacity for evolution, genetic diversity, method of reproduction, and gene flow. Rio de Janeiro is likely the probable center of origin based on several lines of evidence: (i) this parental population harbors the most diverse allele repertoire; (ii) the historical evolution of the epidemic; and (iii) the significant contribution of RJ genotypes hitchhiking through *S. brasiliensis* clades. These findings directly impact public health as cat-transmitted sporotrichosis expands its frontiers throughout America. 

## Figures and Tables

**Table 1:** Statistics for genome-wide retrieved SSRs in *Sporothrix* species.

Strains	Total SSR Loci	Frequency (SSRs/Mb) *	Total SSR Loci with Primer Pair Designed	Total SSR Loci Without Primer Pair Designed	Total Number of Unique Markers
5110	126,286	3803.12	124,525 (98.60%)	1761 (1.39%)	86,689
1099-18	119,583	3693.67	118,265 (98.89%)	1318 (1.10%)	83,104
ATCC58251	118,140	3670.30	117,648 (99.58%)	492 (0.41%)	82,841
SsEM7	120,585	3668.41	119,435 (99.04%)	1150 (0.95%)	83,825
SsMS1	120,118	3676.89	119,136 (99.18%)	982 (0.81%)	83,891
CBS120340	122,335	3654.67	121,189 (99.06%)	1146 (0.93%)	85,279
SS01	122,302	3651.55	121,251 (99.14%)	1051 (0.85%)	85,487
SPA8	143,912	3804.65	141,266 (98.16%)	2646 (1.83%)	98,369

* Microsatellite density (SSRs/Mb) including SSR minimum motif length = 2; maximum motif length = 10; and minimum repeat times = 3.

**Table 2:** Primer sequences, repeat motifs, primer annealing temperature (Tm) for each SSR loci.

SSR Loci	5′ Dye	Primer Sequence (5′-3′) F: Forward; R: Reverse	Motif	Tm (°C)	Expected Amplicon Size (bp) *		
					5110	1099-18	CBS 120340
SSR11	NED	F: TGGAAGCAAAACCATGGTGCCTTTC	CAGTCGCCCC	60.5	87	125	96
	-	R: GAGATCTGCCAAAACGACCGTC		58.2			
SSR50	VIC	F: TTCGACGCCACTAAAGAAGCCG	TGTAAGTGGA	59.7	144	143	195
	-	R: ATTCATTTCTTTTCCAGCCTTGGT		55.4			
SSR61	FAM	F: TTTTCTCGTGAGAAGGTCCCTCCC	TTCTTTTGCC	60.3	136	208	139
	-	R: CCAAAGACGGCGGGAACAAGCG		63.4			
SSR150	FAM	F: TGGAGCACGACACTCTACCTCACC	CAACGG	62.0	389	383	389
	-	R: GTCGAGGCGGCAATACGAGATG		60.1			
SSR181	NED	F: AGGACTTGGACGCATCGGTG	GAGCCA	59.8	167	167	209
	-	R: CAATGTGACCCAGACCACCTTTG		58.3			
SSR199	NED	F: CATGCTGTTGTAGATGGGCAGATG	CGCTGC	58.1	570	543	579
	-	R: CAGGTGCAGCAAGAGTATGCGG		60.6			
SSR235	FAM	F: TTGGTCCCGAGAAAGCCCAG	GATCCA	59.7	477	453	429
	-	R: CTGGTGGCGGTCGATGGTGTC		62.8			
SSR307	VIC	F: CTGTAAGGGTGTGTTGTACGTCGTTG	AGCAGA	59.4	355	309	309
	-	R: GATCGCGCAGGTTGCCTATT		58.2			
SSR343	FAM	F: GTGGCCACTGTAGTACGCGAC	TGC	60.1	268	235	230
	-	R: CATACTCGCGGAGTTTCGGATCTC		59.0			
SSR391	NED	F: ACTGTTCCATTCTCCCGACCGAG	CTACAC	60.6	272	302	-
	-	R: GTGCTCAAGTCGTGCAAGGGC		61.5			
SSR408	NED	F: TTGGAAGAAGCGGCAACAAGTGG	CTGGGA	60.4	273	292	265
	-	R: TAGGGCGGCAGGACGGGAAG		64.2			
SSR538	VIC	F: CTTGGAGGTGTTGATGAGTGTCTGC	ACCAGG	59.6	250	256	243
	-	R: CACATCACAGACGACGCTGGAC		60.1			
SSR637	VIC	F: TCGGTTTCCTCGACCAATTCC	ACCG	57.2	191	183	178
	-	R: CATGGTTGCGGTGGCTTATGTCG		60.8			
SSR646	VIC	F: TCTTGTGGCGGAGTGGCTGTC	CAC	62.1	362	362	374
	-	R: GCACATCCTGTGTCCAGCGAAC		60.8			
SSR661	FAM	F: TACCGTTGCGCTTTGCCCCATTTG	TGTTT	62.8	371	253	253
	-	R: CATCGGCAGCCATTCCATTTGTG		59.3			

* Amplicon size was determined based on sequences originated from the whole genome sequencing of *S. brasiliensis* 5110 (AWTV01), *S. schenckii* 1099-18 (AXCR01), and *S. globosa* CBS120340 (LVYW01).

**Table 3:** Polymorphic statistics for multiplexes M1–M5 of SSR markers in *Sporothrix* species.

Multiplex ID	Species (n)	Alleles (n)	*H*	*PIC*	*E*	*H_avp_*	*MI*	*D*
M1	*S. brasiliensis* (97)	26	0.7908	0.7644	1.0000	0.7908	0.7908	0.4045
	*S. schenckii* (49)	43	0.8980	0.8899	1.0000	0.8980	0.8980	0.8370
	*S. globosa* (34)	13	0.8089	0.7845	1.0000	0.8089	0.8089	0.4908
	Overall (180)	64	0.9010	0.8941	1.0000	0.9010	0.9010	0.7813
M2	*S. brasiliensis* (97)	13	0.7291	0.6796	1.0000	0.7291	0.7291	0.2531
	*S. schenckii* (49)	29	0.8740	0.8628	1.0000	0.8740	0.8740	0.7633
	*S. globosa* (34)	8	0.7141	0.6599	1.0000	0.7141	0.7141	0.1468
	Overall (180)	40	0.8759	0.8637	1.0000	0.8759	0.8759	0.7183
M3	*S. brasiliensis* (97)	18	0.8245	0.8029	1.0000	0.8245	0.8245	0.4825
	*S. schenckii* (49)	28	0.8032	0.7821	1.0000	0.8032	0.8032	0.5986
	*S. globosa* (34)	9	0.7803	0.7473	1.0000	0.7803	0.7803	0.3571
	Overall (180)	40	0.8650	0.8524	1.0000	0.8650	0.8650	0.7202
M4	*S. brasiliensis* (97)	39	0.7785	0.7522	1.0000	0.7785	0.7785	0.3736
	*S. schenckii* (49)	27	0.8523	0.8371	1.0000	0.8523	0.8523	0.6366
	*S. globosa* (34)	17	0.7797	0.7525	1.0000	0.7797	0.7797	0.4504
	Overall (180)	67	0.8636	0.8519	1.0000	0.8636	0.8636	0.7172
M5	*S. brasiliensis* (97)	20	0.6023	0.5381	1.0000	0.6023	0.6023	0.2673
	*S. schenckii* (49)	16	0.7420	0.7004	1.0000	0.7420	0.7420	0.2449
	*S. globosa* (34)	3	0.6699	0.5987	1.0000	0.6699	0.6699	0.0927
	Overall (180)	29	0.7683	0.7392	1.0000	0.7683	0.7683	0.6265
15 SSRs	*S. brasiliensis* (97)	116	0.9057	0.8987	1.0000	0.9057	0.9057	0.3562
	*S. schenckii* (49)	143	0.9077	0.9009	1.0000	0.9077	0.9077	0.6161
	*S. globosa* (34)	50	0.8993	0.8904	1.0000	0.8993	0.8993	0.3075
	Overall (180)	240	0.9159	0.9101	1.0000	0.9159	0.9159	0.7127

*D*: discriminating power; *E*: effective multiplex ratio; *H*: expected heterozygosity; *H_avp_*: mean heterozygosity; *MI*: marker index; *PIC*: polymorphism information content.

**Table 4:** Analysis of molecular variance (AMOVA) shows the partitioning of genetic variation within and between *Sporothrix* species populations.

Group	Source of Variation	df	SS	MS	Est. Var.	%	*p*-Value
*Sporothrix*	Among population	2	1609.687	804.843	14.677	54%	0.0001
(n = 180)	Within population	177	2217.469	12.528	12.528	46%	0.0001
	Total	179	3827.156		27.205	100%	
*S. brasiliensis*	Among population	2	83.248	41.624	1.416	12%	0.0001
(n = 96) *	Within population	93	934.752	10.051	10.051	88%	0.0001
	Total	95	1018.000		11.467	100%	

(df = degree of freedom, SS = sum of squares, MS = mean squares, Est. var. = estimate of variance, % = percentage of the total variation, *p*-value is based on 9999 permutations). Group *Sporothrix* (n = 180): Pop1 is 97 *S. brasiliensis* isolates; Pop2 is 49 *S. schenckii* isolates; Pop 3 is 34 *S. globosa* isolates. Group *S. brasiliensis* (n = 96): * Pop1 is 18 isolates (South); Pop2 is 67 isolates (Southeast); Pop3 is 11 isolates (Northeast). Isolate Ss104 (Central-West Brazil) was removed from the *S. brasiliensis* dataset.

**Table 5:** Linkage disequilibrium (*LD*) analysis in the complete *Sporothrix* isolate set (n = 180) and within members of the clinical clade.

LD Characteristic	Clinical Clade (n = 180)	*S. brasiliensis* (n = 97)	*S. schenckii* (n = 49)	*S. globosa* (n = 34)
Total number of markers	240	240	240	240
Number of markers pairs	28,560	28,560	28,560	28,560
Number of markers pairs at 0 ≤ *p* ≤ 0.001	871 (3.40%)	102 (0.35%)	87 (0.30%)	7 (0.024%)
Number of markers pairs at 0.001 ≤ *p* ≤ 0.01	330 (1.15%)	59 (0.20%)	74 (0.25%)	10 (0.035%)
Number of markers pairs at *r*^2^ = 1	4 (0.014%)	2 (0.007%)	8 (0.028%)	0
Mean *r*^2^	0.02714	0.04104	0.04957	0.05840
Mean *D′*	0.8910	0.7750	0.8405	0.6170

## Data Availability

The data presented in this study are available within the article and Supplementary Materials.

## Supplementary Materials

The following supporting information can be downloaded at https://www.mdpi.com/article/10.3390/jof9030354/s1, Supplementary Table S1. *Sporothrix* isolates used in this study. Supplementary Table S2. Genomes of *Sporothrix* species were retrieved from the NCBI Genome database (https://www.ncbi.nlm.nih.gov/genome, accessed on 15 March 2018) for in silico analysis. Supplementary Table S3. Polymorphic statistics calculated individually for SSR markers applied in medically relevant *Sporothrix* species. Supplementary Table S4. *Sporothrix* species genotypes (alleles) characterized using 15 microsatellite markers. Supplementary Table S5. Inferred ancestry of individuals using the software Structure for *S. brasiliensis* (n = 97), *S. schenckii* (n = 49), and *S. globosa* (n = 34). Supplementary Figure S1. Panel of SSR markers used for genotyping medically relevant *Sporothrix* species. The forward primer was labeled at the 5’ region. 6-Carboxyfluorescein (FAM; 520 nm), 2′-chloro-phenyl-1,4-dichloro-6-carboxyfluorescein (VIC; 555 nm) or benzofluorotrichloro-carboxy-fluorescein (NED; 576 nm). The multiplex PCR’s and cycling conditions are presented. We considered a good quality DNA extraction when the OD 260/280 ratio was between 1.8 and 2.0, and an amplicon was detected by PCR using the primers ITS1 and ITS4, indicating that the sample was free of PCR inhibitors. Supplementary Figure S2. Summary of performance of SSR markers for *S. brasiliensis* (n = 97, yellow line), *S. schenckii* (n = 47, red line), and *S. globosa* (n = 37, green line) using receiver operating characteristic curves. The area under the ROC curve was great (AUC above 0.956) for all primer pairs (except marker SSR391 for *S. globosa* isolates), indicating excellent performance. Supplementary Figure S3. The annotated UPGMA dendrogram (clades), based on SSR markers, generated with a total of 15 SSR markers for 180 *Sporothrix* isolates originated worldwide. The dendrogram shows cophenetic correlation values (circles are represented by color ranges between green-yellow-orange-red according to decreasing cophenetic correlation) for a given clade and its standard deviation (grey bar). For pairwise genetic distances calculation, the Dice similarity coefficient was used. Supplementary Figure S4. Annotated Minimum Spanning Trees (MSTs) showing the genetic relationship among 180 *Sporothrix* isolates using 15 SSRs markers. MST was created in the software BioNumerics v7.6.
