# Supplementary material for: High-Throughput Microsatellite Markers Development for Genetic Characterization of Emerging Sporothrix Species
Source: J Fungi (Basel). 2023 Mar 14;9(3):354. doi: 10.3390/jof9030354 (PMC10054832; doi:10.3390/jof9030354)
Supplement: Supplementary file 1 [file jof-09-00354-s001.zip › jof-2263357-supplementary.pdf]

Journal of Fungi

Supplementary files

High-throughput Microsatellite Markers Development for Genetic Characterization of Emerging *Sporothrix* species

Luiza Chaves de Miranda Leonhardt Losada [1,2,†], Ruan Campos Monteiro [1,†], Jamile Ambrósio de Carvalho [1,†], Ferry Hagen [3,4,5], Matthew C. Fisher [6], Bram Spruijtenburg [7,8], Jacques F. Meis [7,8,9], Theun de Groot [7,8], Sarah Santos Gonçalves [10], Ricardo Negroni [11], Rui Kano [12], Alexandro Bonifaz [13], Zoilo Pires de Camargo [1,2] and Anderson Messias Rodrigues [1,2,*]

1 Laboratory of Emerging Fungal Pathogens, Department of Microbiology, Immunology, and Parasitology, Discipline of Cellular Biology, Federal University of São Paulo (UNIFESP), São Paulo 04023062, Brazil; luiza.leonhardt@gmail.com (L.C.d.M.L.L.); ruanmonteirolj@gmail.com (R.C.M.); jamileambrosio@hotmail.com (J.A.d.C.); zpcamargo1@gmail.com (Z.P.d.C.)
2 Department of Medicine, Discipline of Infectious Diseases, Federal University of São Paulo (UNIFESP), São Paulo 04023062, Brazil
3 Department of Medical Mycology, Westerdijk Fungal Biodiversity Institute, Uppsalalaan 8, 3584 CT Utrecht, The Netherlands; f.hagen@gmail.com
4 Institute for Biodiversity and Ecosystem Dynamics, University of Amsterdam, Sciencepark 904, 1098 XH Amsterdam, The Netherlands
5 Department of Medical Microbiology, University Medical Center Utrecht, Heidelberglaan 100, 3584 CX Utrecht, The Netherlands
6 Medical Research Council Center for Global Infectious Disease Analysis, Department of Infectious Disease Epidemiology, School of Public Health, Imperial College London, London W2 1PG, UK; matthew.fisher@imperial.ac.uk
7 Department of Medical Microbiology and Infectious Diseases, Canisius-Wilhelmina Hospital, 6532 SZ Nijmegen, The Netherlands; b.spruijtenburg@cwz.nl (B.S.); jacques.meis@gmail.com (J.F.M.); t.degroot@cwz.nl (T.d.G.)
8 Center of Expertise in Mycology Radboud University Medical Center/Canisius-Wilhelmina Hospital, 6532 SZ Nijmegen, The Netherlands
9 Department I of Internal Medicine, Faculty of Medicine, University of Cologne, and Excellence Center for Medical Mycology, University Hospital Cologne, 50931 Cologne, Germany
10 Infectious Diseases Postgraduate Program, Center for Research in Medical Mycology, Federal University of Espírito Santo (UFES), Espírito Santo 29043900, Brazil; sarahunifesp@yahoo.com.br
11 Mycology Unit of the Infectious Diseases Hospital Francisco Javier Muñiz, Reference Center of Mycology of Buenos Aires City, Uspallata 2272, Buenos Aires, Argentina; ricnegroni@hotmail.com
12 Teikyo University Institute of Medical Mycology (TIMM), 359 Otsuka, Tokyo 192-0395, Japan; kanou.rui.ng@teikyo-u.ac.jp
13 Dermatology Service, Mycology Department, Hospital General de México, "Dr. Eduardo Liceaga", Balmis 148, Colonia Doctores, 03020, Mexico City, Mexico; a_bonifaz@yahoo.com.mx
* Correspondence: amrodrigues@unifesp.br or amrodrigues.amr@gmail.com; Tel.: +55-1155764551 (ext. 1540)
† These authors contributed equally to this work.

Supplementary Table S1. *Sporothrix* isolates used in this study.

Isolate	Other codes	Species	Source	Year of isolation	*MAT*	Origin	mtDNA PCR	Ref.
Ss05	CBS132985	*S. brasiliensis*	Cat	2004	1-1	MG, Brazil	1157 bp	[1-6]
Ss08	-	*S. brasiliensis*	Human	2004	1-1	MG, Brazil	1157 bp	[1-6]
Ss25	CBS132988	*S. brasiliensis*	Human	2004	1-2	PR, Brazil	1157 bp	[1-6]
Ss34	-	*S. brasiliensis*	Human	2004	1-1	PR, Brazil	1157 bp	[1-6]
Ss37	-	*S. brasiliensis*	Human	2004	1-2	PR, Brazil	1157 bp	[1-6]
Ss43	-	*S. brasiliensis*	Human	1997	1-1	CE, Brazil	1157 bp	[1-6]
Ss53	CBS132989	*S. brasiliensis*	Cat	2004	1-1	RS, Brazil	1157 bp	[1-6]
Ss54	CBS132990	*S. brasiliensis*	Cat	2004	1-1	RS, Brazil	1157 bp	[1-6]
Ss55	-	*S. brasiliensis*	Human	2004	1-1	RS, Brazil	1157 bp	[1-6]
Ss62	CBS132991	*S. brasiliensis*	Human	2004	1-1	ES, Brazil	1157 bp	[1-6]
Ss66	-	*S. brasiliensis*	Human	1999	1-2	RJ, Brazil	1157 bp	[1-6]
Ss67	-	*S. brasiliensis*	Human	2004	1-2	RJ, Brazil	1157 bp	[1-6]
Ss95	-	*S. brasiliensis*	Human	1999	1-2	RJ, Brazil	1157 bp	[1-6]
Ss99	-	*S. brasiliensis*	Human	1998	1-2	RJ, Brazil	1157 bp	[1-6]
Ss104	-	*S. brasiliensis*	Human	2004	1-2	MT, Brazil	1157 bp	[1-6]
Ss128	-	*S. brasiliensis*	Human	2004	1-1	SP, Brazil	1157 bp	[1-6]
Ss151	CBS132994	*S. brasiliensis*	Dog	2006	1-1	RS, Brazil	1157 bp	[1-6]
Ss152	CBS132995	*S. brasiliensis*	Cat	2006	1-1	RS, Brazil	1157 bp	[1-6]
Ss153	CBS132996	*S. brasiliensis*	Cat	2006	1-1	RS, Brazil	1157 bp	[1-6]
Ss154	-	*S. brasiliensis*	Cat	2006	1-1	RS, Brazil	1157 bp	[1-6]
Ss171	CBS132999	*S. brasiliensis*	Cat	2010	1-1	PR, Brazil	1157 bp	[1-6]
Ss172	CBS133000	*S. brasiliensis*	Cat	2010	1-1	PR, Brazil	1157 bp	[1-6]
Ss174	CBS133004	*S. brasiliensis*	Cat	2010	1-1	PR, Brazil	1157 bp	[1-6]
Ss177	FMR8309	*S. brasiliensis*	Human	2007	1-2	RJ, Brazil	1157 bp	[1-6]
Ss178	CBS120339	*S. brasiliensis*	Human	2007	1-1	RJ, Brazil	1157 bp	[1-6]
Ss226	CBS133003	*S. brasiliensis*	Cat	2010	1-2	SP, Brazil	1157 bp	[1-6]
Ss227	CBS133004	*S. brasiliensis*	Dog	2010	1-1	SP, Brazil	1157 bp	[1-6]
Ss245	CBS133005	*S. brasiliensis*	Cat	2010	1-2	RJ, Brazil	1157 bp	[1-6]
Ss246	CBS133002	*S. brasiliensis*	Cat	2010	1-2	RJ, Brazil	1157 bp	[1-6]
Ss247	CBS133006	*S. brasiliensis*	Cat	2010	1-2	RJ, Brazil	1157 bp	[1-6]
Ss248	CBS133007	*S. brasiliensis*	Cat	2010	1-2	RJ, Brazil	1157 bp	[1-6]
Ss251	CBS133010	*S. brasiliensis*	Cat	2010	1-2	RJ, Brazil	1157 bp	[1-6]
Ss252	CBS133011	*S. brasiliensis*	Cat	2010	1-2	RJ, Brazil	1157 bp	[1-6]
Ss256	CBS133015	*S. brasiliensis*	Cat	2010	1-2	RJ, Brazil	1157 bp	[1-6]
Ss265	CBS133020	*S. brasiliensis*	Human	2010	1-1	MG, Brazil	1157 bp	[1-6]
Ss292	-	*S. brasiliensis*	Cat	2011	1-2	SP, Brazil	1157 bp	[1-6]
Ss294	-	*S. brasiliensis*	Cat	2011	1-1	SP, Brazil	1157 bp	[1-6]
Ss330	-	*S. brasiliensis*	Cat	2012	1-1	SP, Brazil	1157 bp	[1-6]
Ss602	-	*S. brasiliensis*	Human	2016	1-2	ES, Brazil	1157 bp	[1-6]

**Isolate**	**Other codes**	**Species**	**Source**	**Year of isolation**	***MAT***	**Origin**	**mtDNA PCR**	**Ref.**
Ss605	-	*S. brasiliensis*	Human	2016	1-1	ES, Brazil	1157 bp	[1-6]
Ss607	-	*S. brasiliensis*	Human	2017	1-2	PE, Brazil	1157 bp	[1-6]
Ss608	-	*S. brasiliensis*	Human	2017	1-2	PE, Brazil	1157 bp	[1-6]
Ss609	-	*S. brasiliensis*	Human	2017	1-2	PE, Brazil	1157 bp	[1-6]
Ss610	-	*S. brasiliensis*	Human	2017	1-2	PE, Brazil	1157 bp	[1-6]
Ss611	-	*S. brasiliensis*	Human	2017	1-2	PE, Brazil	1157 bp	[1-6]
Ss612	-	*S. brasiliensis*	Human	2017	1-2	PE, Brazil	1157 bp	[1-6]
Ss613	-	*S. brasiliensis*	Human	2017	1-2	PE, Brazil	1157 bp	[1-6]
Ss614	-	*S. brasiliensis*	Human	2017	1-2	PE, Brazil	1157 bp	[1-6]
Ss615	-	*S. brasiliensis*	Human	2017	1-2	PE, Brazil	1157 bp	[1-6]
Ss616	-	*S. brasiliensis*	Human	2017	1-2	PE, Brazil	1157 bp	[1-6]
Ss630	-	*S. brasiliensis*	Cat	2018	1-2	SP, Brazil	1157 bp	[1-6]
Ss631	-	*S. brasiliensis*	Cat	2018	1-2	SP, Brazil	1157 bp	[1-6]
Ss633	-	*S. brasiliensis*	Cat	2018	1-2	SP, Brazil	1157 bp	[1-6]
Ss634	-	*S. brasiliensis*	Cat	2018	1-2	SP, Brazil	1157 bp	[1-6]
Ss645	-	*S. brasiliensis*	Cat	2018	1-2	SP, Brazil	1157 bp	[1-6]
Ss647	-	*S. brasiliensis*	Cat	2018	1-2	SP, Brazil	1157 bp	[1-6]
Ss653	-	*S. brasiliensis*	Cat	2018	1-2	SP, Brazil	1157 bp	[1-6]
Ss654	-	*S. brasiliensis*	Cat	2018	1-2	SP, Brazil	1157 bp	[1-6]
Ss663	-	*S. brasiliensis*	Cat	2018	1-2	SP, Brazil	1157 bp	[1-6]
Ss668	-	*S. brasiliensis*	Cat	2018	1-2	SP, Brazil	1157 bp	[1-6]
Ss669	-	*S. brasiliensis*	Cat	2018	1-2	SP, Brazil	1157 bp	[1-6]
Ss670	-	*S. brasiliensis*	Cat	2018	1-2	SP, Brazil	1157 bp	[1-6]
Ss673	-	*S. brasiliensis*	Cat	2018	1-2	SP, Brazil	1157 bp	[1-6]
Ss676	-	*S. brasiliensis*	Human	2018	1-2	SP, Brazil	1157 bp	[1-6]
Ss677	-	*S. brasiliensis*	Human	2018	1-2	SP, Brazil	1157 bp	[1-6]
Ss681	-	*S. brasiliensis*	Human	2018	1-2	SP, Brazil	1157 bp	[1-6]
Ss683	-	*S. brasiliensis*	Human	2018	1-2	SP, Brazil	1157 bp	[1-6]
Ss684	-	*S. brasiliensis*	Human	2018	1-2	SP, Brazil	1157 bp	[1-6]
Ss697	-	*S. brasiliensis*	Human	2019	1-1	ES, Brazil	1157 bp	[1-6]
Ss698	-	*S. brasiliensis*	Human	2019	1-1	ES, Brazil	1157 bp	[1-6]
Ss699	-	*S. brasiliensis*	Human	2019	1-2	ES, Brazil	1157 bp	[1-6]
Ss700	-	*S. brasiliensis*	Human	2019	1-1	ES, Brazil	1157 bp	[1-6]
Ss701	-	*S. brasiliensis*	Cat	2019	1-2	ES, Brazil	1157 bp	[1-6]
Ss702	-	*S. brasiliensis*	Cat	2019	1-2	ES, Brazil	1157 bp	[1-6]
Ss707	-	*S. brasiliensis*	Human	2019	1-2	ES, Brazil	1157 bp	[1-6]
Ss708	-	*S. brasiliensis*	Human	2019	1-2	ES, Brazil	1157 bp	[1-6]
Ss709	-	*S. brasiliensis*	Cat	2019	1-2	ES, Brazil	1157 bp	[1-6]
Ss711	-	*S. brasiliensis*	Human	2019	1-2	ES, Brazil	1157 bp	[1-6]
Ss718	-	*S. brasiliensis*	Cat	2019	1-2	ES, Brazil	1157 bp	[1-6]

Isolate	Other codes	Species	Source	Year of isolation	*MAT*	Origin	mtDNA PCR	Ref.
Ss742	-	*S. brasiliensis*	Cat	2019	1-1	MG, Brazil	1157 bp	[1-6]
Ss743	-	*S. brasiliensis*	Cat	2019	1-1	MG, Brazil	1157 bp	[1-6]
Ss753	-	*S. brasiliensis*	Cat	2019	1-2	ES, Brazil	1157 bp	[1-6]
Ss756	-	*S. brasiliensis*	Human	2019	1-2	ES, Brazil	1157 bp	[1-6]
Ss758	-	*S. brasiliensis*	Human	2019	1-2	ES, Brazil	1157 bp	[1-6]
Ss1033	-	*S. brasiliensis*	Human	2014	1-2	RJ, Brazil	1157 bp	[1-6]
Ss1034	-	*S. brasiliensis*	Human	2015	1-2	RJ, Brazil	1157 bp	[1-6]
Ss1035	-	*S. brasiliensis*	Human	2016	1-2	RJ, Brazil	1157 bp	[1-6]
Ss1036	-	*S. brasiliensis*	Human	2011	1-2	RJ, Brazil	1157 bp	[1-6]
Ss1037	-	*S. brasiliensis*	Human	2011	1-2	RJ, Brazil	1157 bp	[1-6]
Ss1038	-	*S. brasiliensis*	Human	2016	1-2	RJ, Brazil	1157 bp	[1-6]
Ss1039	-	*S. brasiliensis*	Human	2016	1-2	RJ, Brazil	1157 bp	[1-6]
Ss1040	-	*S. brasiliensis*	Human	2016	1-2	RJ, Brazil	1157 bp	[1-6]
26925	-	*S. brasiliensis*	Human	Unknown	1-2	PR, Brazil	1157 bp	[1-6]
26926	-	*S. brasiliensis*	Human	Unknown	1-2	PR, Brazil	1157 bp	[1-6]
1878	-	*S. brasiliensis*	Human	Unknown	1-1	PR, Brazil	1157 bp	[1-6]
2320	-	*S. brasiliensis*	Human	Unknown	1-1	RS, Brazil	1157 bp	[1-6]
2625	-	*S. brasiliensis*	Human	Unknown	1-1	RS, Brazil	1157 bp	[1-6]
Ss01	CBS132961	*S. schenckii*	Cat	2004	1-2	SP, Brazil	557 bp	[1-6]
Ss03	CBS132963	*S. schenckii*	Human	2004	1-2	RS, Brazil	1157 bp	[1-6]
Ss04	-	*S. schenckii*	Human	2004	1-2	RS, Brazil	1157 bp	[1-6]
Ss13	-	*S. schenckii*	Human	2004	1-2	MG, Brazil	557 bp	[1-6]
Ss16	-	*S. schenckii*	Human	2004	1-1	PI, Brazil	1157 bp	[1-6]
Ss17	-	*S. schenckii*	Human	2004	1-2	PR, Brazil	557 bp	[1-6]
Ss40	-	*S. schenckii*	Human	2003	1-2	CE, Brazil	557 bp	[1-6]
Ss58	-	*S. schenckii*	Human	2004	1-2	SP, Brazil	557 bp	[1-6]
Ss61	-	*S. schenckii*	Environmental	2004	1-1	SP, Brazil	557 bp	[1-6]
Ss63	CBS132968	*S. schenckii*	Human	2004	1-2	ES, Brazil	557 bp	[1-6]
Ss90	-	*S. schenckii*	Human	1999	1-1	RJ, Brazil	557 bp	[1-6]
Ss107	-	*S. schenckii*	Human	2004	1-2	MG, Brazil	1157 bp	[1-6]
Ss110	-	*S. schenckii*	Human	2004	1-2	MG, Brazil	1157 bp	[1-6]
Ss126	-	*S. schenckii*	Human	2003	1-2	SP, Brazil	557 bp	[1-6]
Ss141	CBS132975	*S. schenckii*	Human	2005	1-1	DF, Brazil	557 bp	[1-6]
Ss143	-	*S. schenckii*	Human	2005	1-1	PA, Brazil	557 bp	[1-6]
Ss158	-	*S. schenckii*	Human	2006	1-2	AM, Brazil	557 bp	[1-6]
Ss159	CBS132976	*S. schenckii*	Human	1934	1-2	Japan	1157 bp	[1-6]
Ss160	-	*S. schenckii*	Human	1990	1-1	Mexico	557 bp	[1-6]
Ss161	-	*S. schenckii*	Human	2007	1-2	Mexico	557 bp	[1-6]
Ss162	CBS132977	*S. schenckii*	Environmental	2007	1-2	Mexico	1157 bp	[1-6]
Ss163	-	*S. schenckii*	Human	2007	1-2	Peru	1157 bp	[1-6]

**Isolate**	**Other codes**	**Species**	**Source**	**Year of isolation**	***MAT***	**Origin**	**mtDNA PCR**	**Ref.**
Ss164	-	*S. schenckii*	Human	2007	1-2	Peru	557 bp	[1-6]
Ss167	CBS132978	*S. schenckii*	Environmental	2007	1-2	Peru	1157 bp	[1-6]
Ss175	SP01	*S. schenckii*	Human	Unknown	1-2	Italy	557 bp	[1-6]
Ss185	CBS359.36	*S. schenckii*	Human	Unknown	1-1	USA	557 bp	[1-6]
Ss196	-	*S. schenckii*	Human	1972	1-2	SP, Brazil	N/A	[1-6]
Ss452	-	*S. schenckii*	Human	2013	1-1	Venezuela	557 bp	[1-6]
Ss453	-	*S. schenckii*	Human	2013	1-1	Venezuela	557 bp	[1-6]
Ss454	-	*S. schenckii*	Human	2013	1-1	Venezuela	557 bp	[1-6]
Ss455	-	*S. schenckii*	Human	2013	1-1	Venezuela	557 bp	[1-6]
Ss459	-	*S. schenckii*	Human	2013	1-1	Venezuela	557 bp	[1-6]
Ss465	-	*S. schenckii*	Human	2013	1-1	Venezuela	557 bp	[1-6]
Ss476	-	*S. schenckii*	Human	Unknown	1-1	Mexico	557 bp	[1-6]
Ss479	-	*S. schenckii*	Human	Unknown	1-1	Mexico	557 bp	[1-6]
Ss480	-	*S. schenckii*	Human	Unknown	1-2	Mexico	557 bp	[1-6]
Ss482	-	*S. schenckii*	Human	Unknown	1-2	Mexico	557 bp	[1-6]
Ss493	-	*S. schenckii*	Human	Unknown	1-2	Argentina	1157 bp	[1-6]
Ss495	-	*S. schenckii*	Human	Unknown	1-2	Argentina	557 bp	[1-6]
Ss496	-	*S. schenckii*	Human	Unknown	1-2	Uruguay	557 bp	[1-6]
Ss499	-	*S. schenckii*	Human	Unknown	1-1	Argentina	1157 bp	[1-6]
Ss526	-	*S. schenckii*	Human	2013	1-2	Mexico	557 bp	[1-6]
Ss537	-	*S. schenckii*	Human	2013	1-2	Mexico	N/A	[1-6]
Ss538	-	*S. schenckii*	Human	2013	1-1	Mexico	N/A	[1-6]
Ss539	-	*S. schenckii*	Human	2013	1-1	Mexico	1157 bp	[1-6]
Ss570	-	*S. schenckii*	Human	2015	1-1	Mexico	N/A	[1-6]
Ss576	-	*S. schenckii*	Human	2015	1-1	Mexico	N/A	[1-6]
Ss581	-	*S. schenckii*	Human	2015	1-1	Mexico	N/A	[1-6]
Ss696	-	*S. schenckii*	Human	2019	1-2	ES, Brazil	557 bp	[1-6]
Ss06	CBS132922	*S. globosa*	Human	2002	1-1	MG, Brazil	557 bp	[1-6]
Ss41	CBS132923	*S. globosa*	Human	2002	1-2	CE, Brazil	557 bp	[1-6]
Ss49	CBS132924	*S. globosa*	Human	2004	1-2	GO, Brazil	557 bp	[1-6]
Ss179	CBS120340	*S. globosa*	Human	Unknown	1-2	Spain	557 bp	[1-6]
Ss180	CBS130104	*S. globosa*	Human	Unknown	1-1	Spain	557 bp	[1-6]
Ss211	-	*S. globosa*	Human	1960	1-1	SP, Brazil	557 bp	[1-6]
Ss236	CBS132925	*S. globosa*	Human	2010	1-2	MG, Brazil	557 bp	[1-6]
Ss376	-	*S. globosa*	Human	Unknown	1-1	ES, Brazil	557 bp	[1-6]
Ss443	-	*S. globosa*	Human	2013	1-1	Venezuela	557 bp	[1-6]
Ss444	-	*S. globosa*	Human	2013	1-1	Venezuela	557 bp	[1-6]
Ss445	-	*S. globosa*	Human	2013	1-1	Venezuela	557 bp	[1-6]
Ss446	-	*S. globosa*	Human	2013	1-1	Venezuela	557 bp	[1-6]
Ss448	-	*S. globosa*	Human	2013	1-1	Venezuela	557 bp	[1-6]

**Isolate**	**Other codes**	**Species**	**Source**	**Year of isolation**	***MAT***	**Origin**	**mtDNA PCR**	**Ref.**
Ss449	-	*S. globosa*	Human	2013	1-1	Venezuela	557 bp	[1-6]
Ss450	-	*S. globosa*	Human	2013	1-1	Venezuela	557 bp	[1-6]
Ss456	-	*S. globosa*	Human	2013	1-1	Venezuela	557 bp	[1-6]
Ss457	-	*S. globosa*	Human	2013	1-1	Venezuela	557 bp	[1-6]
Ss460	-	*S. globosa*	Human	2013	1-1	Venezuela	557 bp	[1-6]
Ss467	-	*S. globosa*	Human	2013	1-1	SP, Brazil	557 bp	[1-6]
Ss471	-	*S. globosa*	Human	2013	1-2	Chile	557 bp	[1-6]
Ss472	-	*S. globosa*	Environmental	2013	1-2	Chile	557 bp	[1-6]
Ss489	-	*S. globosa*	Human	Unknown	1-1	Mexico	557 bp	[1-6]
Ss492	-	*S. globosa*	Human	Unknown	1-1	Argentina	557 bp	[1-6]
Ss520	-	*S. globosa*	Human	2015	1-1	RJ, Brazil	557 bp	[1-6]
Ss521	-	*S. globosa*	Human	2016	1-1	RS, Brazil	557 bp	[1-6]
Ss522	-	*S. globosa*	Human	2016	1-1	RS, Brazil	557 bp	[1-6]
Ss524	-	*S. globosa*	Human	2016	1-1	RS, Brazil	557 bp	[1-6]
Ss525	-	*S. globosa*	Human	2016	1-1	RS, Brazil	557 bp	[1-6]
Ss545	-	*S. globosa*	Human	2013	1-1	Mexico	557 bp	[1-6]
Ss583	CBS140866	*S. globosa*	Human	2017	1-1	Japan	557 bp	[1-6]
Ss584	CBS140867	*S. globosa*	Cat	2017	1-1	Japan	557 bp	[1-6]
Ss585	CBS140868	*S. globosa*	Human	2017	1-1	Japan	557 bp	[1-6]
Ss586	CBS141044	*S. globosa*	Human	2017	1-2	Japan	557 bp	[1-6]
Ss587	KMU2920	*S. globosa*	Human	2017	1-1	Japan	557 bp	[1-6]

Supplementary Table S2. Genomes of *Sporothrix* species were retrieved from the NCBI Genome database (https://www.ncbi.nlm.nih.gov/genome) for *in silico* analysis.

**Strain**	**Species**	**Source**	**Origin**	**INSDC1 (WGS)**	**Total length**	**BioProject**	**Ref.**
5110	*S. brasiliensis*	Feline sporotrichosis	Brazil	AWTV01	33.2 Mb	PRJNA218075	[7]
ATCC58251	*S. schenckii*	Human sporotrichosis	USA	AWEQ01	32.5 Mb	PRJNA217088	[8]
1099-18	*S. schenckii*	Human sporotrichosis	USA	AXCR01	32.5 Mb	PRJNA218070	[7]
SsMS1	*S. schenckii*	Human sporotrichosis	Colombia	PGUU01	32.6 Mb	PRJNA401003	[9]
SsEM7	*S. schenckii*	Human sporotrichosis	Colombia	NTMI01	32.8 Mb	PRJNA401003	[9]
CBS120340	*S. globosa*	Human sporotrichosis	Spain	LVYW01	33.4 Mb	PRJNA315855	[10]
SS01	*S. globosa*	Human sporotrichosis	China	LVYX01	33.4 Mb	PRJNA315862	[10]
SPA8	*S. pallida*	Soil	Spain	JNEX02	37.8 Mb	PRJNA248334	[11]

Supplementary Table S3. Polymorphic statistics calculated individually for SSR markers applied in medically relevant *Sporothrix* species.

SSR loci	Species (n)	Alleles (n)	*H*	*PIC*	*E*	H_{avp}	*MI*	*D*	PCR success rate (%)
SSR235	*S. brasiliensis* (97)	13	0.8007	0.7781	1.00	0.8007	0.8007	0.8091	94.85
	S. schenckii (49)	13	0.7947	0.7733	1.00	0.7947	0.7947	0.8112	97.96
	S. globosa (34)	3	0.4550	0.3749	1.00	0.4550	0.4550	0.4688	100
	Overall (180)	27	0.9072	0.9004	1.00	0.9072	0.9072	0.9122	96.67
SSR408	*S. brasiliensis* (97)	5	0.1728	0.1660	1.00	0.1728	0.1728	0.1746	98.97
	S. schenckii (49)	13	0.8147	0.7962	1.00	0.8147	0.8147	0.8316	91.84
	S. globosa (34)	3	0.2128	0.1993	1.00	0.2128	0.2128	0.2193	100
	Overall (180)	16	0.7067	0.6841	1.00	0.7067	0.7067	0.7107	97.22
SSR637	*S. brasiliensis* (97)	8	0.2274	0.2203	1.00	0.2274	0.2274	0.2298	100
	S. schenckii (49)	17	0.8505	0.8398	1.00	0.8505	0.8505	0.8682	100
	S. globosa (34)	7	0.7612	0.7248	1.00	0.7612	0.7612	0.7843	100
	Overall (180)	21	0.7169	0.7023	1.00	0.7169	0.7169	0.7209	100
SSR199	*S. brasiliensis* (97)	4	0.3199	0.2975	1.00	0.3199	0.3199	0.3232	98.97
	S. schenckii (49)	11	0.8205	0.8000	1.00	0.8205	0.8205	0.8376	100
	S. globosa (34)	4	0.1644	0.1572	1.00	0.1644	0.1644	0.1693	100
	Overall (180)	14	0.7575	0.7368	1.00	0.7575	0.7575	0.7618	99.44
SSR538	*S. brasiliensis* (97)	5	0.3709	0.3161	1.00	0.3709	0.3709	0.3748	100
	S. schenckii (49)	12	0.6239	0.5685	1.00	0.6239	0.6239	0.6369	100
	S. globosa (34)	3	0.0000	0.0000	1.00	0.0000	0.0000	0.0000	100
	Overall (180)	16	0.7201	0.6762	1.00	0.7201	0.7201	0.7241	100
SSR61	*S. brasiliensis* (97)	4	0.0606	0.0600	1.00	0.0606	0.0606	0.0612	100
	S. schenckii (49)	6	0.7988	0.7708	1.00	0.7988	0.7988	0.8155	100
	S. globosa (34)	1	0.2630	0.2491	1.00	0.2630	0.2630	0.2709	100
	Overall (180)	10	0.6653	0.6302	1.00	0.6653	0.6653	0.6690	100
SSR181	*S. brasiliensis* (97)	2	0.2755	0.2375	1.00	0.2755	0.2755	0.2784	100
	S. schenckii (49)	7	0.2957	0.2899	1.00	0.2957	0.2957	0.3019	100
	S. globosa (34)	4	0.5277	0.4398	1.00	0.5277	0.5277	0.5437	100
	Overall (180)	10	0.5060	0.4804	1.00	0.5060	0.5060	0.5089	100
SSR307	*S. brasiliensis* (97)	5	0.5350	0.4571	1.00	0.5350	0.5350	0.5406	100
	S. schenckii (49)	6	0.6089	0.5652	1.00	0.6089	0.6089	0.6216	100
	S. globosa (34)	3	0.4550	0.3749	1.00	0.4550	0.4550	0.4688	100
	Overall (180)	9	0.8092	0.7855	1.00	0.8092	0.8092	0.8137	100
SSR343	*S. brasiliensis* (97)	11	0.6220	0.5909	1.00	0.6220	0.6220	0.6284	98.97
	S. schenckii (49)	15	0.8546	0.8401	1.00	0.8546	0.8546	0.8724	100
	S. globosa (34)	2	0.0571	0.0555	1.00	0.0571	0.0571	0.0588	100
	Overall (180)	21	0.8333	0.8166	1.00	0.8333	0.8333	0.8380	99.44
SSR11	*S. brasiliensis* (97)	5	0.1558	0.1519	1.00	0.1558	0.1558	0.1574	100
	S. schenckii (49)	12	0.8163	0.7947	1.00	0.8163	0.8163	0.8333	100
	S. globosa (34)	4	0.3097	0.2932	1.00	0.3097	0.3097	0.3191	100
	Overall (180)	16	0.6543	0.6306	1.00	0.6543	0.6543	0.6579	100
SSR646	*S. brasiliensis* (97)	4	0.0406	0.0402	1.00	0.0406	0.0406	0.0410	100
	S. schenckii (49)	8	0.6647	0.6122	1.00	0.6647	0.6647	0.6786	100
	S. globosa (34)	10	0.8374	0.8183	1.00	0.8374	0.8374	0.8627	97.06
	Overall (180)	15	0.5862	0.5603	1.00	0.5862	0.5862	0.5895	99.44

SSR loci	Species (n)	Alleles (n)	*H*	*PIC*	*E*	H_{avp}	*MI*	*D*	PCR success rate (%)
SSR661	*S. brasiliensis* (97)	30	0.9130	0.9073	1.00	0.9130	0.9130	0.9225	100
	S. schenckii (49)	7	0.3898	0.3793	1.00	0.3898	0.3898	0.3980	97.96
	S. globosa (34)	3	0.1644	0.1572	1.00	0.1644	0.1644	0.1693	100
	Overall (180)	36	0.8993	0.8921	1.00	0.8993	0.8993	0.9043	99.44
SSR150	*S. brasiliensis* (97)	6	0.2232	0.2095	1.00	0.2232	0.2232	0.2255	98.97
	S. schenckii (49)	4	0.6406	0.6054	1.00	0.6406	0.6406	0.6539	100
	S. globosa (34)	2	0.0000	0.0000	1.00	0.0000	0.0000	0.0000	91.18
	Overall (180)	7	0.7100	0.6776	1.00	0.7100	0.7100	0.7140	97.78
SSR391	*S. brasiliensis* (97)	6	0.4139	0.3750	1.00	0.4139	0.4139	0.4182	100
	S. schenckii (49)	9	0.0791	0.0776	1.00	0.0791	0.0791	0.0808	97.96
	S. globosa (34)	0	0.0571	0.0555	1.00	0.0571	0.0571	0.0588	0.00
	Overall (180)	13	0.6711	0.6081	1.00	0.6711	0.6711	0.6749	80.56
SSR50	*S. brasiliensis* (97)	8	0.1564	0.1536	1.00	0.1564	0.1564	0.1581	98.97
	S. schenckii (49)	3	0.0000	0.0000	1.00	0.0000	0.0000	0.0000	100
	S. globosa (34)	1	0.2128	0.1993	1.00	0.2128	0.2128	0.2193	97.06
	Overall (180)	9	0.4878	0.4221	1.00	0.4878	0.4878	0.4906	98.89

D: discriminating power; *E*: effective multiplex ratio; *H*: expected heterozygosity; H_{avp}: mean heterozygosity; *MI*: marker index; *PIC*: polymorphism information content.

Supplementary Table S4. *Sporothrix* species genotypes (alleles) characterized using 15 microsatellite markers.

Isolate	Species	SSR 235	SSR 408	SSR 637	SSR 199	SSR 538	SSR 61	SSR 181	SSR 307	SSR 343	SSR 11	SSR 646	SSR 661	SSR150	SSR 391	SSR 50
Ss34	*S. brasiliensis*	20	11	9	4	9	1	5	7	1	5	13	8	4	2	4
Ss227	*S. brasiliensis*	19	4	9	7	4	5	5	8	17	5	11	15	4	2	1
Ss25	*S. brasiliensis*	14	4	15	7	4	3	5	8	21	5	11	21	4	2	2
Ss37	*S. brasiliensis*	15	4	2	7	4	3	4	8	6	5	11	20	4	2	2
Ss128	*S. brasiliensis*	21	14	13	7	12	5	5	3	1	5	11	9	4	2	3
Ss171	*S. brasiliensis*	21	14	13	7	12	5	5	3	1	5	11	10	4	2	3
Ss172	*S. brasiliensis*	22	14	13	7	12	5	4	3	1	5	11	10	4	2	3
Ss174	*S. brasiliensis*	22	14	13	7	12	5	5	3	1	5	11	10	4	2	3
Ss226	*S. brasiliensis*	22	14	13	7	12	5	5	3	1	5	11	10	0	2	0
1878	*S. brasiliensis*	21	14	13	7	12	5	4	3	1	5	11	10	3	3	3
Ss630	*S. brasiliensis*	0	4	13	7	4	3	5	9	17	5	11	30	4	2	2
Ss633	*S. brasiliensis*	0	4	13	7	4	3	5	9	17	5	11	29	4	2	2
Ss668	*S. brasiliensis*	0	4	13	7	4	3	5	9	17	5	11	30	4	2	2
Ss670	*S. brasiliensis*	0	4	13	7	4	3	5	9	17	5	11	30	4	2	2
Ss53	*S. brasiliensis*	14	4	13	7	4	3	5	8	6	3	11	18	4	2	2
Ss54	*S. brasiliensis*	15	4	13	7	4	3	5	8	6	5	11	18	4	1	2
Ss55	*S. brasiliensis*	13	4	13	7	4	3	5	8	6	5	11	17	4	2	2
Ss151	*S. brasiliensis*	14	4	13	7	4	3	5	8	6	5	12	18	4	2	2
Ss152	*S. brasiliensis*	15	4	13	7	4	3	5	8	6	5	11	19	4	2	2
Ss153	*S. brasiliensis*	14	4	13	7	4	3	5	8	6	5	11	20	4	2	2
Ss154	*S. brasiliensis*	14	4	13	7	4	3	5	8	6	5	11	18	4	2	2
Ss605	*S. brasiliensis*	14	4	13	6	4	5	5	8	2	5	11	12	5	2	6
Ss697	*S. brasiliensis*	14	4	13	7	4	5	4	8	2	5	11	13	5	2	2
Ss698	*S. brasiliensis*	13	4	13	7	4	5	5	8	2	5	11	13	4	2	2
Ss699	*S. brasiliensis*	13	4	13	7	4	5	5	8	2	5	11	20	4	3	3
Ss742	*S. brasiliensis*	18	4	12	7	4	5	5	8	2	5	11	10	4	3	3
Ss743	*S. brasiliensis*	16	4	12	7	4	5	5	8	2	5	11	10	4	3	3
2320	*S. brasiliensis*	15	4	13	7	4	3	4	8	6	2	11	19	3	2	2
2625	*S. brasiliensis*	13	4	13	7	4	3	4	8	6	5	11	19	4	2	2
Ss43	*S. brasiliensis*	25	4	13	7	4	3	5	8	6	5	11	23	4	2	3
Ss62	*S. brasiliensis*	25	4	13	7	4	3	5	8	6	5	11	23	4	2	3
Ss294	*S. brasiliensis*	25	4	13	7	4	3	5	8	6	11	11	22	4	2	3
Ss330	*S. brasiliensis*	25	4	13	7	4	3	5	8	6	5	11	22	4	2	3
Ss05	*S. brasiliensis*	20	4	12	7	3	5	5	8	2	5	11	26	4	2	2

Isolate	Species	SSR 235	SSR 408	SSR 637	SSR 199	SSR 538	SSR 61	SSR 181	SSR 307	SSR 343	SSR 11	SSR 646	SSR 661	SSR150	SSR 391	SSR 50
Ss08	*S. brasiliensis*	23	4	13	7	4	5	5	8	2	5	11	15	4	2	3
Ss66	*S. brasiliensis*	21	4	13	7	4	3	5	9	17	5	11	31	4	2	2
Ss67	*S. brasiliensis*	22	4	13	7	3	3	5	9	17	5	11	27	4	2	2
Ss95	*S. brasiliensis*	21	4	13	7	4	3	5	9	17	5	11	33	4	2	2
Ss99	*S. brasiliensis*	19	4	12	7	4	5	5	8	14	5	11	14	4	2	2
Ss104	*S. brasiliensis*	22	4	13	7	3	3	5	9	17	5	11	29	4	2	2
Ss177	*S. brasiliensis*	22	4	13	7	4	3	5	9	17	11	11	31	4	2	2
Ss178	*S. brasiliensis*	21	4	13	7	4	5	5	8	2	5	11	16	4	2	3
Ss245	*S. brasiliensis*	21	4	13	7	3	3	5	9	19	5	11	31	4	2	2
Ss246	*S. brasiliensis*	21	4	13	7	3	3	4	9	17	5	11	33	4	2	2
Ss247	*S. brasiliensis*	21	4	13	7	3	3	4	9	17	5	11	33	4	2	2
Ss248	*S. brasiliensis*	21	4	13	7	4	3	5	9	17	5	11	28	4	2	2
Ss251	*S. brasiliensis*	21	4	13	7	4	3	5	9	17	5	11	35	4	2	2
Ss252	*S. brasiliensis*	21	4	13	7	3	3	5	9	17	5	11	34	4	2	2
Ss256	*S. brasiliensis*	21	4	13	7	4	3	4	9	17	5	11	29	4	2	2
Ss292	*S. brasiliensis*	24	4	13	7	4	3	5	8	20	5	11	25	4	2	3
Ss602	*S. brasiliensis*	21	4	13	7	4	3	4	9	17	5	11	34	4	2	2
Ss607	*S. brasiliensis*	22	4	13	7	4	3	5	9	17	5	11	31	4	2	2
Ss608	*S. brasiliensis*	21	4	13	7	3	3	5	9	17	5	11	31	4	2	2
Ss609	*S. brasiliensis*	23	4	13	7	4	3	5	9	17	5	11	31	4	2	2
Ss610	*S. brasiliensis*	22	4	13	7	4	3	5	9	17	5	11	31	4	2	2
Ss611	*S. brasiliensis*	21	4	13	7	4	3	5	9	17	5	11	31	4	2	2
Ss612	*S. brasiliensis*	21	4	13	7	4	3	5	9	17	5	11	31	4	2	2
Ss613	*S. brasiliensis*	21	4	13	7	3	3	5	9	17	11	11	31	4	2	2
Ss614	*S. brasiliensis*	22	4	13	7	4	3	5	9	17	5	11	31	4	2	2
Ss615	*S. brasiliensis*	21	4	13	7	4	3	5	9	17	5	11	31	4	2	2
Ss616	*S. brasiliensis*	21	4	13	7	4	3	5	9	17	5	11	31	4	2	2
Ss631	*S. brasiliensis*	22	4	13	7	4	3	5	9	17	5	11	30	4	2	2
Ss634	*S. brasiliensis*	23	4	13	7	4	3	5	9	17	5	11	29	4	2	2
Ss645	*S. brasiliensis*	20	4	13	0	4	3	5	9	17	5	11	29	4	2	2
Ss647	*S. brasiliensis*	22	4	13	7	4	3	5	9	17	5	11	29	4	2	2
Ss653	*S. brasiliensis*	22	4	13	7	4	3	5	9	17	5	11	30	4	2	2
Ss654	*S. brasiliensis*	22	4	13	7	4	3	4	9	0	5	11	29	4	3	2
Ss663	*S. brasiliensis*	22	4	13	7	4	3	5	9	17	5	11	30	4	2	2
Ss669	*S. brasiliensis*	22	4	13	7	4	3	5	9	17	5	11	30	4	2	2

Isolate	Species	SSR 235	SSR 408	SSR 637	SSR 199	SSR 538	SSR 61	SSR 181	SSR 307	SSR 343	SSR 11	SSR 646	SSR 661	SSR150	SSR 391	SSR 50
Ss673	*S. brasiliensis*	22	4	13	7	4	3	4	9	17	5	11	30	4	2	2
Ss676	*S. brasiliensis*	22	4	13	7	4	3	5	9	17	5	11	30	4	2	2
Ss677	*S. brasiliensis*	22	4	13	7	4	2	4	9	17	5	11	30	4	2	2
Ss681	*S. brasiliensis*	22	4	13	7	4	3	5	9	17	5	11	30	4	2	2
Ss683	*S. brasiliensis*	22	4	13	7	4	3	5	9	17	5	11	30	4	2	2
Ss684	*S. brasiliensis*	22	4	13	7	4	3	5	9	17	5	11	30	4	2	2
Ss700	*S. brasiliensis*	22	4	12	7	4	5	4	8	2	5	11	22	4	2	2
Ss701	*S. brasiliensis*	21	4	13	7	4	3	5	9	17	5	11	32	4	2	2
Ss702	*S. brasiliensis*	21	4	13	7	4	3	4	9	17	5	11	32	6	9	7
Ss707	*S. brasiliensis*	22	4	13	7	4	3	5	9	17	5	11	32	4	2	2
Ss708	*S. brasiliensis*	22	4	13	7	3	3	5	9	17	5	11	32	7	13	8
Ss709	*S. brasiliensis*	22	4	13	7	4	3	5	9	17	5	11	32	3	3	5
Ss711	*S. brasiliensis*	21	4	13	7	4	3	5	9	17	5	11	32	4	3	3
Ss718	*S. brasiliensis*	19	4	13	7	4	5	5	8	2	5	11	20	4	3	3
26925	*S. brasiliensis*	23	4	13	7	4	3	5	9	17	11	11	31	4	2	2
26926	*S. brasiliensis*	23	4	13	7	4	3	5	9	16	5	11	31	4	3	2
Ss753	*S. brasiliensis*	21	4	13	7	3	3	5	9	17	5	11	31	4	2	2
Ss756	*S. brasiliensis*	22	4	13	7	4	3	5	9	17	4	11	32	4	2	2
Ss1033	*S. brasiliensis*	21	4	13	7	4	3	5	9	17	5	11	32	4	2	2
Ss1034	*S. brasiliensis*	21	4	13	7	4	3	5	9	17	5	11	31	4	2	2
Ss1035	*S. brasiliensis*	21	4	13	7	4	3	4	9	17	5	11	29	4	2	2
Ss1036	*S. brasiliensis*	21	4	13	7	4	3	5	9	17	5	11	31	4	2	2
Ss1037	*S. brasiliensis*	21	4	13	7	4	3	5	9	17	5	11	31	4	2	2
Ss1038	*S. brasiliensis*	21	4	13	7	4	3	5	9	17	5	11	28	4	2	2
Ss1039	*S. brasiliensis*	21	4	13	7	4	3	5	9	17	5	11	36	4	2	2
Ss1040	*S. brasiliensis*	19	4	12	7	4	5	5	8	9	4	11	20	4	2	2
Ss265	*S. brasiliensis*	21	9	1	7	4	3	5	3	2	5	11	11	4	2	3
Ss758	*S. brasiliensis*	0	0	18	7	4	5	5	8	2	5	11	20	4	2	2
Ss06	*S. globosa*	1	3	4	11	1	6	6	2	4	9	6	5	0	0	9
Ss41	*S. globosa*	1	3	5	5	1	6	8	2	3	9	5	5	4	0	9
Ss49	*S. globosa*	1	3	5	11	2	6	8	5	4	9	8	5	4	0	9
Ss179	*S. globosa*	1	3	7	11	1	6	8	5	4	9	14	5	4	0	9
Ss180	*S. globosa*	2	3	7	11	1	6	8	2	4	9	10	5	0	0	9
Ss236	*S. globosa*	2	3	4	11	1	6	8	5	4	9	14	5	4	0	9
Ss376	*S. globosa*	2	3	6	5	1	6	8	2	4	9	0	4	0	0	0

Isolate	Species	SSR 235	SSR 408	SSR 637	SSR 199	SSR 538	SSR 61	SSR 181	SSR 307	SSR 343	SSR 11	SSR 646	SSR 661	SSR1 50	SSR 391	SSR 50
Ss443	*S. globosa*	1	2	8	11	1	6	6	2	4	9	4	5	4	0	9
Ss444	*S. globosa*	2	3	6	11	1	6	8	5	4	9	14	5	4	0	9
Ss445	*S. globosa*	2	3	7	11	1	6	6	2	4	9	3	5	4	0	9
Ss446	*S. globosa*	2	3	5	11	1	6	8	2	4	9	4	5	4	0	9
Ss448	*S. globosa*	1	2	4	11	2	6	6	2	4	9	5	5	4	0	9
Ss449	*S. globosa*	2	3	6	11	1	6	6	2	4	7	5	5	4	0	9
Ss456	*S. globosa*	2	3	5	11	1	6	6	2	4	8	2	5	4	0	9
Ss457	*S. globosa*	2	3	6	11	1	6	6	2	4	9	2	5	4	0	9
Ss460	*S. globosa*	2	3	7	11	1	6	6	2	4	9	3	5	4	0	9
Ss467	*S. globosa*	2	3	5	11	1	6	8	2	4	9	4	5	4	0	9
Ss471	*S. globosa*	2	3	7	11	1	6	8	5	4	9	14	5	4	0	9
Ss472	*S. globosa*	2	3	5	11	1	6	8	5	4	8	14	5	4	0	9
Ss489	*S. globosa*	2	3	7	11	1	6	8	2	4	11	3	5	4	0	9
Ss492	*S. globosa*	2	3	5	11	1	6	6	2	4	9	3	5	4	0	9
Ss520	*S. globosa*	2	3	6	11	1	6	8	2	4	9	3	5	4	0	9
Ss521	*S. globosa*	1	3	7	11	1	6	6	2	4	9	5	5	4	0	9
Ss522	*S. globosa*	2	3	5	11	1	6	6	2	4	9	4	5	4	0	9
Ss524	*S. globosa*	2	3	5	11	1	6	6	2	4	9	4	5	4	0	9
Ss525	*S. globosa*	1	2	6	11	1	6	8	2	4	9	3	5	4	0	9
Ss545	*S. globosa*	1	3	5	11	1	6	8	2	4	9	3	5	4	0	9
Ss583	*S. globosa*	2	3	5	11	1	6	8	5	4	11	14	5	4	0	9
Ss584	*S. globosa*	2	3	4	11	1	6	8	5	4	9	15	5	4	0	9
Ss585	*S. globosa*	2	3	6	11	1	6	8	5	4	9	4	5	4	0	9
Ss586	*S. globosa*	1	3	5	10	1	6	9	2	4	9	3	5	4	0	9
Ss587	*S. globosa*	2	3	6	11	1	6	8	5	4	9	3	5	4	0	9
Ss211	*S. globosa*	2	3	11	5	1	6	5	2	4	9	5	4	4	0	9
Ss450	*S. globosa*	7	1	13	9	14	6	8	1	4	8	1	24	1	0	9
Ss90	*S. schenckii*	10	13	9	1	5	9	5	4	6	14	11	2	2	10	3
Ss143	*S. schenckii*	9	13	9	1	5	8	5	4	6	14	11	2	2	10	3
Ss175	*S. schenckii*	10	13	9	1	5	7	5	4	6	15	11	2	2	10	3
Ss185	*S. schenckii*	10	13	9	1	5	10	4	4	6	14	11	2	2	10	3
Ss459	*S. schenckii*	9	13	9	11	15	5	5	1	3	6	4	2	2	5	3
Ss465	*S. schenckii*	11	13	9	12	15	5	1	1	3	5	4	2	2	5	3
Ss476	*S. schenckii*	9	13	9	1	5	8	5	4	6	14	11	2	2	10	3
Ss479	*S. schenckii*	9	13	9	1	5	8	5	4	6	16	12	3	2	10	3

**Isolate**	**Species**	**SSR 235**	**SSR 408**	**SSR 637**	**SSR 199**	**SSR 538**	**SSR 61**	**SSR 181**	**SSR 307**	**SSR 343**	**SSR 11**	**SSR 646**	**SSR 661**	**SSR150**	**SSR 391**	**SSR 50**
Ss482	*S. schenckii*	10	13	9	1	5	7	5	4	6	15	11	2	2	10	3
Ss526	*S. schenckii*	12	11	9	1	5	8	4	4	6	14	11	2	2	12	3
Ss16	*S. schenckii*	8	7	9	8	9	4	2	6	1	5	11	6	2	10	3
Ss107	*S. schenckii*	8	7	10	8	7	5	5	5	10	5	13	7	2	11	3
Ss110	*S. schenckii*	10	6	6	9	13	5	5	1	7	2	3	2	2	5	3
Ss167	*S. schenckii*	11	7	3	3	8	7	0	3	5	6	9	4	2	5	3
Ss493	*S. schenckii*	10	8	5	13	12	7	5	3	14	10	11	2	2	8	4
Ss499	*S. schenckii*	10	8	5	14	12	7	5	3	2	12	11	3	2	8	2
Ss539	*S. schenckii*	10	10	9	2	5	7	10	4	6	14	11	2	2	7	3
Ss58	*S. schenckii*	6	6	5	11	13	5	5	1	15	5	4	2	2	5	3
Ss158	*S. schenckii*	6	10	9	10	13	5	5	1	18	6	4	2	2	5	3
Ss570	*S. schenckii*	6	5	2	10	13	5	5	1	3	5	4	2	2	5	3
Ss141	*S. schenckii*	6	6	17	11	12	5	5	1	10	5	4	2	2	5	3
Ss480	*S. schenckii*	6	16	1	10	13	5	5	1	3	11	4	2	2	5	3
Ss537	*S. schenckii*	6	12	1	10	13	5	5	1	3	1	4	2	2	4	3
Ss13	*S. schenckii*	6	13	13	10	13	5	7	1	3	6	4	2	2	5	3
Ss454	*S. schenckii*	6	13	14	13	15	5	5	1	3	4	4	2	2	5	3
Ss495	*S. schenckii*	6	13	13	10	13	5	5	1	7	6	5	2	2	5	3
Ss03	*S. schenckii*	6	13	9	10	13	5	5	1	8	5	5	2	2	6	3
Ss04	*S. schenckii*	5	13	9	10	13	5	5	1	8	6	4	2	2	5	3
Ss159	*S. schenckii*	6	13	5	11	13	5	5	1	13	6	7	2	2	5	3
Ss196	*S. schenckii*	4	15	9	9	3	4	5	1	18	11	5	0	2	0	3
Ss452	*S. schenckii*	6	13	5	11	15	7	5	1	3	6	4	2	2	5	3
Ss164	*S. schenckii*	6	4	15	11	16	7	5	1	5	5	3	2	2	5	3
Ss453	*S. schenckii*	11	13	14	11	15	7	5	1	3	6	4	2	2	5	3
Ss455	*S. schenckii*	6	4	14	11	15	5	5	1	3	6	4	2	2	5	3
Ss61	*S. schenckii*	6	6	14	11	13	5	5	1	7	5	4	1	2	5	3
Ss126	*S. schenckii*	6	6	13	11	10	5	5	1	5	6	4	2	2	6	3
Ss496	*S. schenckii*	3	9	14	11	13	5	5	1	14	6	4	2	2	5	3
Ss538	*S. schenckii*	6	8	12	1	6	7	5	5	5	13	11	8	2	8	3
Ss576	*S. schenckii*	5	8	12	1	6	7	5	5	5	13	11	8	2	8	3
Ss581	*S. schenckii*	0	8	11	1	6	7	5	5	6	13	11	8	2	8	3
Ss01	*S. schenckii*	10	6	13	10	10	5	5	1	12	5	4	2	2	5	3
Ss63	*S. schenckii*	10	6	13	10	10	5	5	1	12	6	4	2	2	5	3
Ss696	*S. schenckii*	8	6	11	9	13	5	5	1	5	6	4	2	2	5	3

Isolate	Species	SSR 235	SSR 408	SSR 637	SSR 199	SSR 538	SSR 61	SSR 181	SSR 307	SSR 343	SSR 11	SSR 646	SSR 661	SSR1 50	SSR 391	SSR 50
Ss17	*S. schenckii*	10	6	15	10	10	5	5	1	11	5	4	2	2	5	3
Ss40	*S. schenckii*	6	8	16	10	10	5	5	1	9	4	4	2	2	5	3
Ss160	*S. schenckii*	27	0	21	1	5	8	5	4	6	14	11	2	2	10	3
Ss161	*S. schenckii*	27	0	21	1	5	7	5	4	6	14	11	2	2	10	3
Ss162	*S. schenckii*	28	0	19	10	13	5	5	2	3	11	4	2	2	5	3
Ss163	*S. schenckii*	26	0	20	3	11	7	3	3	5	6	9	4	2	5	3

Supplementary Table S5. Inferred ancestry of individuals using the software STRUCTURE for *S. brasiliensis* (n=97), *S. schenckii* (n=49), and *S. globosa* (n=34).

STRUCTURE		*K* = 3			*K* = 4				*K* = 5				
**Isolate**	**Cluster**	**Pop1**	**Pop2**	**Pop3**	**Pop1**	**Pop2**	**Pop3**	**Pop4**	**Pop1**	**Pop2**	**Pop3**	**Pop4**	**Pop5**
Ss34	1	0.199	0.004	0.80	0.141	0.004	0.005	0.851	0.024	0.004	0.003	0.013	0.956
Ss227	1	0.918	0.002	0.08	0.902	0.028	0.002	0.067	0.774	0.023	0.002	0.132	0.069
Ss25	1	0.948	0.003	0.05	0.932	0.048	0.003	0.018	0.884	0.009	0.002	0.017	0.088
Ss37	1	0.990	0.002	0.01	0.984	0.009	0.002	0.005	0.977	0.005	0.002	0.009	0.006
Ss128	1	0.983	0.002	0.02	0.74	0.007	0.002	0.25	0.973	0.004	0.002	0.008	0.013
Ss171	1	0.992	0.002	0.01	0.76	0.005	0.002	0.233	0.985	0.003	0.002	0.007	0.004
Ss172	1	0.992	0.002	0.01	0.762	0.004	0.002	0.231	0.985	0.003	0.002	0.007	0.004
Ss174	1	0.992	0.002	0.01	0.76	0.005	0.002	0.233	0.985	0.003	0.002	0.007	0.003
Ss226	1	0.993	0.002	0.01	0.756	0.004	0.002	0.237	0.982	0.003	0.002	0.009	0.004
1878	1	0.992	0.002	0.01	0.739	0.004	0.002	0.255	0.985	0.003	0.002	0.005	0.005
Ss630	1	0.996	0.002	0.00	0.993	0.002	0.002	0.002	0.006	0.002	0.002	0.987	0.002
Ss633	1	0.996	0.002	0.00	0.993	0.002	0.002	0.002	0.006	0.002	0.002	0.987	0.002
Ss668	1	0.996	0.002	0.00	0.993	0.002	0.002	0.002	0.005	0.002	0.002	0.988	0.002
Ss670	1	0.996	0.002	0.00	0.993	0.002	0.002	0.002	0.006	0.002	0.002	0.987	0.002
Ss53	1	0.994	0.002	0.00	0.989	0.003	0.002	0.006	0.977	0.003	0.002	0.012	0.006
Ss54	1	0.989	0.007	0.00	0.983	0.003	0.009	0.006	0.976	0.003	0.005	0.011	0.006
Ss55	1	0.994	0.002	0.00	0.989	0.003	0.002	0.005	0.971	0.003	0.002	0.018	0.006
Ss151	1	0.990	0.002	0.01	0.98	0.003	0.002	0.015	0.975	0.004	0.002	0.012	0.006
Ss152	1	0.996	0.002	0.00	0.993	0.002	0.002	0.003	0.978	0.002	0.002	0.015	0.002
Ss153	1	0.996	0.002	0.00	0.993	0.002	0.002	0.003	0.979	0.002	0.002	0.014	0.002
Ss154	1	0.996	0.002	0.00	0.993	0.002	0.002	0.003	0.978	0.002	0.002	0.015	0.002
Ss605	1	0.965	0.002	0.03	0.942	0.018	0.002	0.038	0.872	0.003	0.002	0.005	0.117
Ss697	1	0.995	0.002	0.00	0.992	0.003	0.002	0.003	0.985	0.002	0.002	0.007	0.004
Ss698	1	0.996	0.002	0.00	0.992	0.003	0.002	0.002	0.985	0.002	0.002	0.008	0.003
Ss699	1	0.994	0.002	0.00	0.989	0.005	0.002	0.004	0.987	0.003	0.002	0.005	0.003
Ss742	1	0.990	0.002	0.01	0.941	0.008	0.002	0.049	0.982	0.003	0.002	0.004	0.008
Ss743	1	0.988	0.002	0.01	0.941	0.008	0.002	0.049	0.981	0.003	0.002	0.004	0.009
2320	1	0.991	0.002	0.01	0.982	0.008	0.002	0.008	0.976	0.004	0.002	0.008	0.009
2625	1	0.996	0.002	0.00	0.993	0.002	0.002	0.003	0.978	0.002	0.002	0.015	0.002
Ss43	1	0.994	0.002	0.00	0.99	0.003	0.002	0.005	0.984	0.003	0.002	0.009	0.003
Ss62	1	0.995	0.002	0.00	0.99	0.003	0.002	0.005	0.983	0.003	0.002	0.009	0.003
Ss294	1	0.993	0.002	0.00	0.989	0.004	0.002	0.005	0.958	0.006	0.004	0.024	0.008
Ss330	1	0.995	0.002	0.00	0.99	0.003	0.002	0.005	0.984	0.003	0.002	0.009	0.003
Ss05	1	0.991	0.002	0.01	0.983	0.005	0.002	0.01	0.806	0.003	0.002	0.095	0.094
Ss08	1	0.994	0.002	0.00	0.987	0.005	0.002	0.005	0.929	0.003	0.002	0.058	0.008
Ss66	1	0.996	0.002	0.00	0.994	0.002	0.002	0.002	0.005	0.002	0.002	0.988	0.002
Ss67	1	0.995	0.002	0.00	0.991	0.003	0.002	0.004	0.008	0.004	0.002	0.965	0.021
Ss95	1	0.996	0.002	0.00	0.994	0.002	0.002	0.002	0.005	0.002	0.002	0.988	0.002

STRUCTURE		K = 3			K = 4				K = 5				
**Isolate**	**Cluster**	**Pop1**	**Pop2**	**Pop3**	**Pop1**	**Pop2**	**Pop3**	**Pop4**	**Pop1**	**Pop2**	**Pop3**	**Pop4**	**Pop5**
Ss99	1	0.963	0.002	0.04	0.944	0.02	0.002	0.033	0.955	0.005	0.002	0.007	0.03
Ss104	1	0.996	0.002	0.00	0.994	0.002	0.002	0.002	0.005	0.002	0.002	0.989	0.002
Ss177	1	0.995	0.002	0.00	0.993	0.003	0.002	0.002	0.005	0.002	0.002	0.988	0.003
Ss178	1	0.991	0.002	0.01	0.983	0.008	0.002	0.006	0.974	0.004	0.002	0.013	0.007
Ss245	1	0.995	0.002	0.00	0.991	0.003	0.002	0.004	0.009	0.004	0.002	0.965	0.021
Ss246	1	0.996	0.002	0.00	0.994	0.002	0.002	0.002	0.005	0.002	0.002	0.989	0.002
Ss247	1	0.996	0.002	0.00	0.994	0.002	0.002	0.002	0.005	0.002	0.002	0.989	0.002
Ss248	1	0.996	0.002	0.00	0.993	0.002	0.002	0.002	0.006	0.002	0.002	0.988	0.003
Ss251	1	0.994	0.002	0.00	0.991	0.003	0.002	0.004	0.012	0.004	0.002	0.965	0.017
Ss252	1	0.996	0.002	0.00	0.993	0.002	0.002	0.002	0.004	0.002	0.002	0.989	0.003
Ss256	1	0.996	0.002	0.00	0.994	0.002	0.002	0.002	0.006	0.002	0.002	0.988	0.002
Ss292	1	0.965	0.002	0.03	0.933	0.018	0.003	0.046	0.86	0.004	0.002	0.03	0.105
Ss602	1	0.996	0.002	0.00	0.994	0.002	0.002	0.002	0.005	0.002	0.002	0.988	0.003
Ss607	1	0.996	0.002	0.00	0.994	0.002	0.002	0.002	0.005	0.002	0.002	0.989	0.002
Ss608	1	0.996	0.002	0.00	0.994	0.002	0.002	0.002	0.004	0.002	0.002	0.989	0.002
Ss609	1	0.996	0.002	0.00	0.994	0.002	0.002	0.002	0.005	0.002	0.002	0.988	0.002
Ss610	1	0.996	0.002	0.00	0.994	0.002	0.002	0.002	0.005	0.002	0.002	0.988	0.002
Ss611	1	0.996	0.002	0.00	0.994	0.002	0.002	0.002	0.005	0.002	0.002	0.988	0.002
Ss612	1	0.996	0.002	0.00	0.994	0.002	0.002	0.002	0.005	0.002	0.002	0.989	0.002
Ss613	1	0.995	0.002	0.00	0.993	0.003	0.002	0.002	0.004	0.003	0.002	0.988	0.003
Ss614	1	0.996	0.002	0.00	0.994	0.002	0.002	0.002	0.005	0.002	0.002	0.988	0.002
Ss615	1	0.996	0.002	0.00	0.994	0.002	0.002	0.002	0.005	0.002	0.002	0.989	0.002
Ss616	1	0.996	0.002	0.00	0.994	0.002	0.002	0.002	0.005	0.002	0.002	0.988	0.002
Ss631	1	0.996	0.002	0.00	0.994	0.002	0.002	0.002	0.005	0.002	0.002	0.989	0.002
Ss634	1	0.996	0.002	0.00	0.994	0.002	0.002	0.002	0.005	0.002	0.002	0.988	0.002
Ss645	1	0.995	0.002	0.00	0.992	0.002	0.002	0.004	0.033	0.003	0.002	0.938	0.023
Ss647	1	0.996	0.002	0.00	0.994	0.002	0.002	0.002	0.006	0.002	0.002	0.988	0.002
Ss653	1	0.996	0.002	0.00	0.994	0.002	0.002	0.002	0.005	0.002	0.002	0.988	0.002
Ss654	1	0.996	0.002	0.00	0.993	0.002	0.002	0.002	0.016	0.002	0.003	0.976	0.003
Ss663	1	0.996	0.002	0.00	0.994	0.002	0.002	0.002	0.005	0.002	0.002	0.988	0.002
Ss669	1	0.996	0.002	0.00	0.994	0.002	0.002	0.002	0.005	0.002	0.002	0.989	0.002
Ss673	1	0.996	0.002	0.00	0.994	0.002	0.002	0.002	0.005	0.002	0.002	0.988	0.002
Ss676	1	0.996	0.002	0.00	0.994	0.002	0.002	0.002	0.005	0.002	0.002	0.988	0.002
Ss677	1	0.995	0.002	0.00	0.991	0.003	0.002	0.004	0.012	0.004	0.002	0.963	0.019
Ss681	1	0.996	0.002	0.00	0.994	0.002	0.002	0.002	0.005	0.002	0.002	0.989	0.002
Ss683	1	0.996	0.002	0.00	0.994	0.002	0.002	0.002	0.005	0.002	0.002	0.988	0.002
Ss684	1	0.996	0.002	0.00	0.994	0.002	0.002	0.002	0.005	0.002	0.002	0.988	0.002
Ss700	1	0.995	0.002	0.00	0.993	0.003	0.002	0.003	0.979	0.002	0.002	0.014	0.003
Ss701	1	0.996	0.002	0.00	0.994	0.002	0.002	0.002	0.005	0.002	0.002	0.989	0.002
Ss702	1	0.979	0.008	0.01	0.964	0.006	0.009	0.02	0.006	0.003	0.003	0.764	0.224

STRUCTURE		K = 3			K = 4				K = 5				
Isolate	Cluster	Pop1	Pop2	Pop3	Pop1	Pop2	Pop3	Pop4	Pop1	Pop2	Pop3	Pop4	Pop5
Ss707	1	0.996	0.002	0.00	0.993	0.002	0.002	0.003	0.005	0.002	0.002	0.989	0.002
Ss708	1	0.976	0.008	0.02	0.96	0.008	0.009	0.023	0.005	0.003	0.003	0.756	0.233
Ss709	1	0.994	0.002	0.00	0.99	0.003	0.002	0.005	0.094	0.003	0.002	0.808	0.093
Ss711	1	0.995	0.002	0.00	0.992	0.003	0.002	0.003	0.082	0.018	0.003	0.883	0.014
Ss718	1	0.994	0.002	0.00	0.99	0.005	0.002	0.004	0.987	0.003	0.002	0.005	0.003
26925	1	0.995	0.002	0.00	0.993	0.003	0.002	0.002	0.005	0.002	0.002	0.987	0.003
26926	1	0.995	0.002	0.00	0.991	0.003	0.002	0.004	0.022	0.004	0.003	0.945	0.027
Ss753	1	0.996	0.002	0.00	0.994	0.002	0.002	0.002	0.004	0.002	0.002	0.989	0.002
Ss756	1	0.995	0.002	0.00	0.991	0.004	0.002	0.002	0.028	0.013	0.002	0.942	0.015
Ss1033	1	0.996	0.002	0.00	0.994	0.002	0.002	0.002	0.005	0.002	0.002	0.989	0.002
Ss1034	1	0.996	0.002	0.00	0.994	0.002	0.002	0.002	0.005	0.002	0.002	0.989	0.002
Ss1035	1	0.996	0.002	0.00	0.994	0.002	0.002	0.002	0.005	0.002	0.002	0.988	0.002
Ss1036	1	0.996	0.002	0.00	0.994	0.002	0.002	0.002	0.005	0.002	0.002	0.989	0.002
Ss1037	1	0.996	0.002	0.00	0.994	0.002	0.002	0.002	0.005	0.002	0.002	0.988	0.002
Ss1038	1	0.996	0.002	0.00	0.994	0.002	0.002	0.002	0.005	0.002	0.002	0.988	0.003
Ss1039	1	0.994	0.002	0.00	0.991	0.003	0.002	0.004	0.01	0.003	0.002	0.965	0.019
Ss1040	1	0.979	0.002	0.02	0.964	0.028	0.002	0.006	0.949	0.015	0.002	0.009	0.024
Ss265	1	0.801	0.002	0.20	0.746	0.176	0.002	0.076	0.77	0.021	0.002	0.048	0.158
Ss758	1	0.991	0.002	0.01	0.986	0.006	0.003	0.005	0.978	0.004	0.003	0.008	0.008
Ss90	2	0.003	0.002	1.00	0.002	0.004	0.002	0.991	0.003	0.984	0.002	0.003	0.008
Ss143	2	0.003	0.002	1.00	0.002	0.004	0.002	0.992	0.003	0.988	0.002	0.003	0.004
Ss175	2	0.003	0.002	1.00	0.002	0.004	0.002	0.992	0.003	0.987	0.002	0.003	0.005
Ss185	2	0.004	0.002	0.99	0.003	0.004	0.002	0.992	0.006	0.972	0.002	0.004	0.015
Ss459	2	0.002	0.003	1.00	0.002	0.976	0.002	0.02	0.002	0.991	0.002	0.002	0.003
Ss465	2	0.003	0.002	1.00	0.002	0.989	0.002	0.007	0.003	0.97	0.002	0.003	0.023
Ss476	2	0.003	0.002	1.00	0.002	0.004	0.002	0.992	0.003	0.989	0.002	0.003	0.004
Ss479	2	0.003	0.002	1.00	0.002	0.003	0.002	0.992	0.004	0.883	0.002	0.002	0.109
Ss482	2	0.003	0.002	1.00	0.002	0.004	0.002	0.992	0.003	0.987	0.002	0.003	0.005
Ss526	2	0.004	0.003	0.99	0.003	0.003	0.002	0.992	0.006	0.779	0.003	0.003	0.209
Ss16	2	0.006	0.002	0.99	0.005	0.025	0.002	0.968	0.006	0.01	0.002	0.003	0.979
Ss107	2	0.003	0.004	0.99	0.005	0.086	0.003	0.905	0.005	0.007	0.002	0.003	0.983
Ss110	2	0.002	0.019	0.98	0.002	0.973	0.019	0.005	0.004	0.912	0.033	0.002	0.049
Ss167	2	0.002	0.002	1.00	0.002	0.459	0.002	0.537	0.002	0.016	0.002	0.002	0.977
Ss493	2	0.003	0.003	0.99	0.003	0.013	0.003	0.982	0.004	0.044	0.003	0.003	0.947
Ss499	2	0.096	0.003	0.90	0.047	0.004	0.003	0.945	0.018	0.007	0.003	0.005	0.967
Ss539	2	0.003	0.003	1.00	0.002	0.006	0.003	0.989	0.003	0.819	0.003	0.002	0.172
Ss58	2	0.003	0.004	0.99	0.003	0.99	0.003	0.004	0.003	0.983	0.004	0.003	0.007
Ss158	2	0.002	0.002	1.00	0.002	0.988	0.002	0.008	0.002	0.983	0.002	0.002	0.01
Ss570	2	0.004	0.002	0.99	0.003	0.991	0.002	0.004	0.006	0.976	0.002	0.003	0.013
Ss141	2	0.003	0.003	0.99	0.004	0.964	0.002	0.03	0.01	0.903	0.003	0.003	0.082

**STRUCTURE**		*K* = 3			*K* = 4				*K* = 5				
**Isolate**	**Cluster**	**Pop1**	**Pop2**	**Pop3**	**Pop1**	**Pop2**	**Pop3**	**Pop4**	**Pop1**	**Pop2**	**Pop3**	**Pop4**	**Pop5**
Ss480	2	0.002	0.002	1.00	0.002	0.993	0.002	0.003	0.003	0.981	0.002	0.003	0.012
Ss537	2	0.002	0.003	1.00	0.002	0.99	0.003	0.004	0.003	0.939	0.003	0.002	0.053
Ss13	2	0.003	0.002	1.00	0.003	0.992	0.002	0.003	0.003	0.986	0.002	0.003	0.005
Ss454	2	0.002	0.002	1.00	0.002	0.99	0.002	0.005	0.003	0.985	0.002	0.002	0.009
Ss495	2	0.004	0.002	0.99	0.003	0.992	0.002	0.003	0.004	0.987	0.003	0.003	0.004
Ss03	2	0.003	0.003	1.00	0.003	0.991	0.002	0.004	0.003	0.986	0.003	0.003	0.005
Ss04	2	0.002	0.002	1.00	0.002	0.985	0.002	0.011	0.002	0.985	0.002	0.002	0.009
Ss159	2	0.002	0.004	0.99	0.002	0.989	0.003	0.005	0.002	0.973	0.004	0.002	0.018
Ss196	2	0.007	0.003	0.99	0.008	0.927	0.003	0.062	0.003	0.044	0.003	0.004	0.946
Ss452	2	0.002	0.004	0.99	0.002	0.99	0.003	0.005	0.002	0.988	0.004	0.002	0.004
Ss164	2	0.012	0.006	0.98	0.01	0.978	0.005	0.008	0.012	0.877	0.007	0.01	0.094
Ss453	2	0.002	0.003	1.00	0.002	0.991	0.002	0.004	0.002	0.989	0.002	0.002	0.004
Ss455	2	0.007	0.003	0.99	0.007	0.988	0.002	0.003	0.008	0.977	0.002	0.008	0.004
Ss61	2	0.003	0.003	1.00	0.003	0.992	0.002	0.003	0.003	0.985	0.003	0.003	0.007
Ss126	2	0.003	0.003	0.99	0.003	0.992	0.003	0.003	0.004	0.985	0.003	0.003	0.005
Ss496	2	0.002	0.003	1.00	0.002	0.986	0.002	0.009	0.005	0.935	0.003	0.002	0.056
Ss538	2	0.003	0.002	0.99	0.003	0.026	0.002	0.969	0.004	0.019	0.002	0.003	0.973
Ss576	2	0.003	0.002	0.99	0.003	0.006	0.002	0.989	0.004	0.006	0.002	0.003	0.985
Ss581	2	0.003	0.003	0.99	0.003	0.004	0.003	0.991	0.006	0.013	0.003	0.003	0.975
Ss01	2	0.005	0.002	0.99	0.004	0.99	0.002	0.004	0.005	0.984	0.002	0.005	0.004
Ss63	2	0.004	0.002	0.99	0.003	0.992	0.002	0.003	0.004	0.988	0.002	0.003	0.003
Ss696	2	0.002	0.002	1.00	0.002	0.962	0.002	0.034	0.002	0.909	0.002	0.002	0.084
Ss17	2	0.003	0.002	1.00	0.003	0.991	0.002	0.004	0.004	0.982	0.002	0.003	0.009
Ss40	2	0.003	0.002	1.00	0.003	0.975	0.002	0.021	0.005	0.903	0.002	0.002	0.088
Ss160	2	0.003	0.002	1.00	0.003	0.003	0.002	0.992	0.004	0.986	0.002	0.003	0.005
Ss161	2	0.003	0.002	1.00	0.003	0.004	0.002	0.992	0.003	0.985	0.002	0.003	0.006
Ss162	2	0.002	0.015	0.98	0.002	0.975	0.017	0.005	0.003	0.887	0.024	0.002	0.083
Ss163	2	0.002	0.003	1.00	0.002	0.437	0.003	0.558	0.002	0.011	0.002	0.002	0.982
Ss06	3	0.002	0.994	0.00	0.002	0.003	0.991	0.004	0.002	0.003	0.983	0.002	0.01
Ss41	3	0.002	0.974	0.02	0.002	0.042	0.954	0.003	0.002	0.039	0.949	0.002	0.008
Ss49	3	0.002	0.995	0.00	0.002	0.003	0.991	0.004	0.002	0.003	0.98	0.002	0.013
Ss179	3	0.002	0.996	0.00	0.002	0.002	0.994	0.002	0.002	0.002	0.991	0.002	0.002
Ss180	3	0.002	0.994	0.00	0.002	0.003	0.991	0.004	0.003	0.003	0.981	0.002	0.011
Ss236	3	0.002	0.996	0.00	0.002	0.002	0.994	0.002	0.002	0.002	0.991	0.002	0.003
Ss376	3	0.003	0.993	0.00	0.002	0.005	0.986	0.007	0.003	0.003	0.983	0.003	0.009
Ss443	3	0.002	0.994	0.00	0.002	0.004	0.99	0.003	0.003	0.004	0.981	0.002	0.01
Ss444	3	0.002	0.996	0.00	0.002	0.002	0.994	0.002	0.002	0.002	0.991	0.002	0.002
Ss445	3	0.002	0.996	0.00	0.002	0.002	0.994	0.002	0.002	0.002	0.991	0.002	0.002
Ss446	3	0.002	0.995	0.00	0.002	0.003	0.993	0.002	0.002	0.003	0.991	0.002	0.002
Ss448	3	0.002	0.996	0.00	0.002	0.002	0.993	0.002	0.002	0.002	0.99	0.002	0.003

STRUCTURE		K = 3			K = 4				K = 5				
Isolate	Cluster	Pop1	Pop2	Pop3	Pop1	Pop2	Pop3	Pop4	Pop1	Pop2	Pop3	Pop4	Pop5
Ss449	3	0.002	0.994	0.00	0.002	0.003	0.992	0.003	0.003	0.003	0.982	0.002	0.011
Ss456	3	0.002	0.996	0.00	0.002	0.002	0.994	0.002	0.002	0.002	0.991	0.002	0.003
Ss457	3	0.002	0.996	0.00	0.002	0.002	0.994	0.002	0.002	0.002	0.991	0.002	0.002
Ss460	3	0.002	0.996	0.00	0.002	0.002	0.994	0.002	0.002	0.002	0.991	0.002	0.002
Ss467	3	0.002	0.995	0.00	0.002	0.003	0.993	0.002	0.002	0.003	0.991	0.002	0.002
Ss471	3	0.002	0.996	0.00	0.002	0.002	0.994	0.002	0.002	0.002	0.991	0.002	0.002
Ss472	3	0.002	0.996	0.00	0.002	0.002	0.994	0.002	0.002	0.002	0.991	0.002	0.003
Ss489	3	0.002	0.995	0.00	0.002	0.003	0.993	0.002	0.002	0.002	0.99	0.003	0.003
Ss492	3	0.002	0.996	0.00	0.002	0.002	0.994	0.002	0.002	0.002	0.991	0.002	0.002
Ss520	3	0.002	0.996	0.00	0.002	0.002	0.994	0.002	0.002	0.002	0.992	0.002	0.002
Ss521	3	0.002	0.996	0.00	0.002	0.002	0.994	0.002	0.002	0.002	0.991	0.002	0.002
Ss522	3	0.002	0.996	0.00	0.002	0.003	0.993	0.002	0.002	0.003	0.991	0.002	0.002
Ss524	3	0.002	0.996	0.00	0.002	0.003	0.993	0.002	0.002	0.003	0.991	0.002	0.002
Ss525	3	0.002	0.996	0.00	0.002	0.002	0.994	0.002	0.002	0.002	0.991	0.002	0.002
Ss545	3	0.002	0.996	0.00	0.002	0.002	0.994	0.002	0.002	0.002	0.991	0.002	0.002
Ss583	3	0.002	0.995	0.00	0.002	0.003	0.992	0.002	0.002	0.003	0.989	0.003	0.003
Ss584	3	0.002	0.995	0.00	0.002	0.003	0.992	0.003	0.003	0.003	0.982	0.002	0.01
Ss585	3	0.002	0.995	0.00	0.002	0.003	0.993	0.002	0.002	0.003	0.991	0.002	0.002
Ss586	3	0.003	0.965	0.03	0.002	0.044	0.948	0.005	0.003	0.036	0.923	0.002	0.037
Ss587	3	0.002	0.996	0.00	0.002	0.002	0.994	0.002	0.002	0.002	0.991	0.002	0.002
Ss211	3	0.010	0.908	0.08	0.008	0.046	0.884	0.063	0.009	0.026	0.871	0.007	0.087
Ss450	3	0.005	0.382	0.61	0.005	0.541	0.404	0.051	0.004	0.005	0.244	0.005	0.742

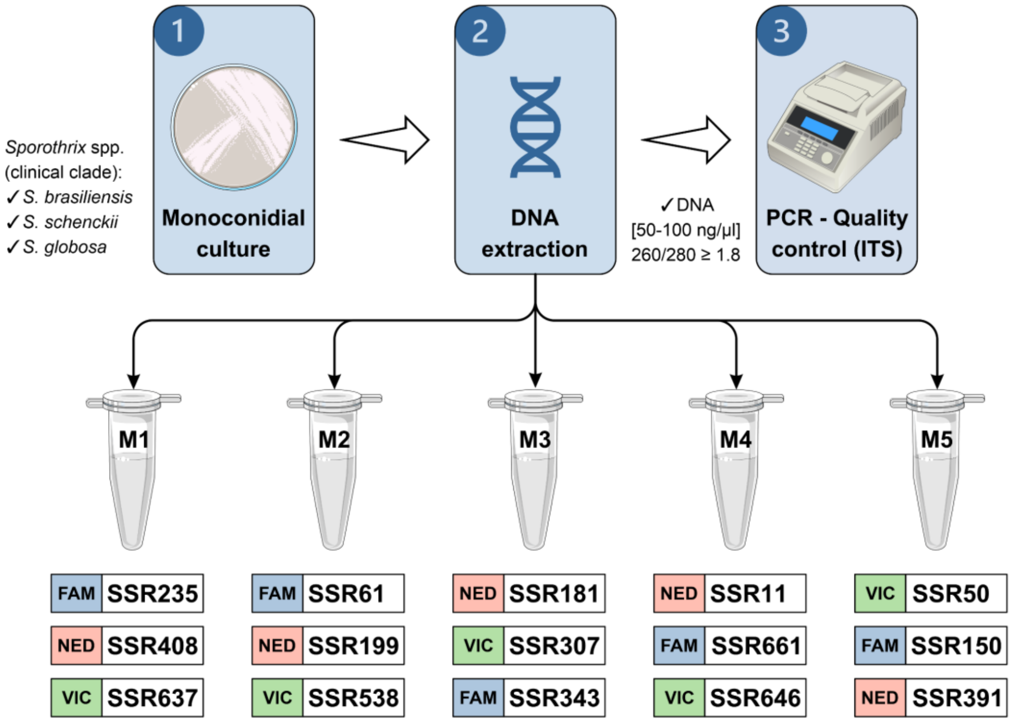

	M1	M2	M3	M4	M5
PCR Master Mix 2 ×	12.5 µl	12.5 µl	12.5 µl	12.5 µl	12.5 µl
Ultra pure water	7.00 µl	9.00 µl	7.50 µl	6.50 µl	7.00 µl
Primer 1 Fw (10 pmol/µl)	0.75 µl 235F	0.25 µl 61F	1.00 µl 181F	1.00 µl 11F	0.75 µl 50F
Primer 1 Rv (10 pmol/µl)	0.75 µl 235R	0.25 µl 61R	1.00 µl 181R	1.00 µl 11R	0.75 µl 50R
Primer 2 Fw (10 pmol/µl)	0.75 µl 408F	0.75 µl 199F	0.50 µl 307F	0.75 µl 661F	0.25 µl 150F
Primer 2 Rv (10 pmol/µl)	0.75 µl 408R	0.75 µl 199R	0.50 µl 307R	0.75 µl 661R	0.25 µl 150R
Primer 3 Fw (10 pmol/µl)	0.75 µl 637F	0.25 µl 538F	0.50 µl 343F	0.75 µl 646F	1.25 µl 391F
Primer 3 Rv (10 pmol/µl)	0.75 µl 637R	0.25 µl 538R	0.50 µl 343R	0.75 µl 646R	1.25 µl 391R
Sporothrix DNA (50 ng)	1.00 µl	1.00 µl	1.00 µl	1.00 µl	1.00 µl
**Total**	25 µl	25 µl	25 µl	25 µl	25 µl

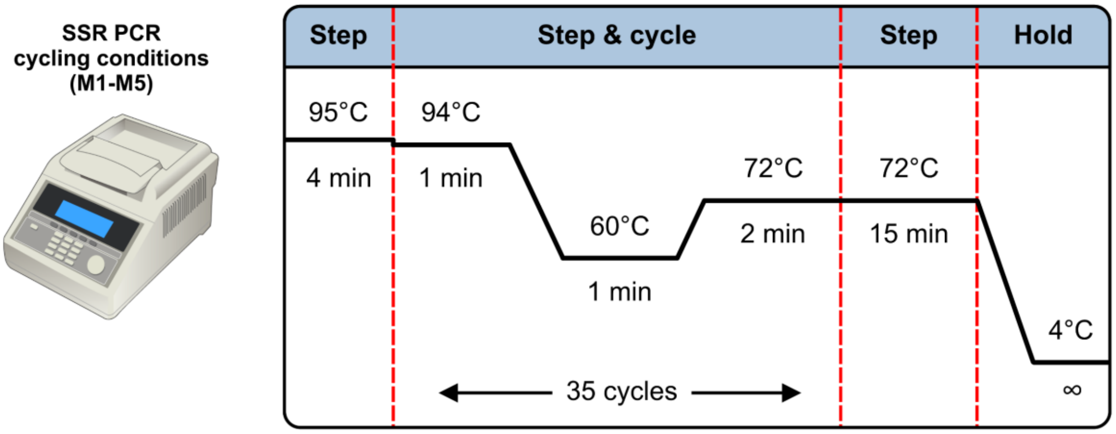

Supplementary Figure S1. Panel of SSR markers used for genotyping medically relevant *Sporothrix* species. The forward primers were labeled at the 5'-region with 6-Carboxyfluorescein (FAM; 520 nm), 2′-chloro-phenyl-1,4-dichloro-6-carboxyfluorescein (VIC; 555 nm) or benzofluorotrichloro-carboxy-fluorescein (NED; 576 nm). The multiplex PCR's and cycling conditions are presented. We considered a good quality DNA extraction when the OD 260/280 ratio was between 1.8–2.0, and an amplicon was detected by PCR using the primers ITS1 and ITS4, indicating that the sample was free of PCR inhibitors.

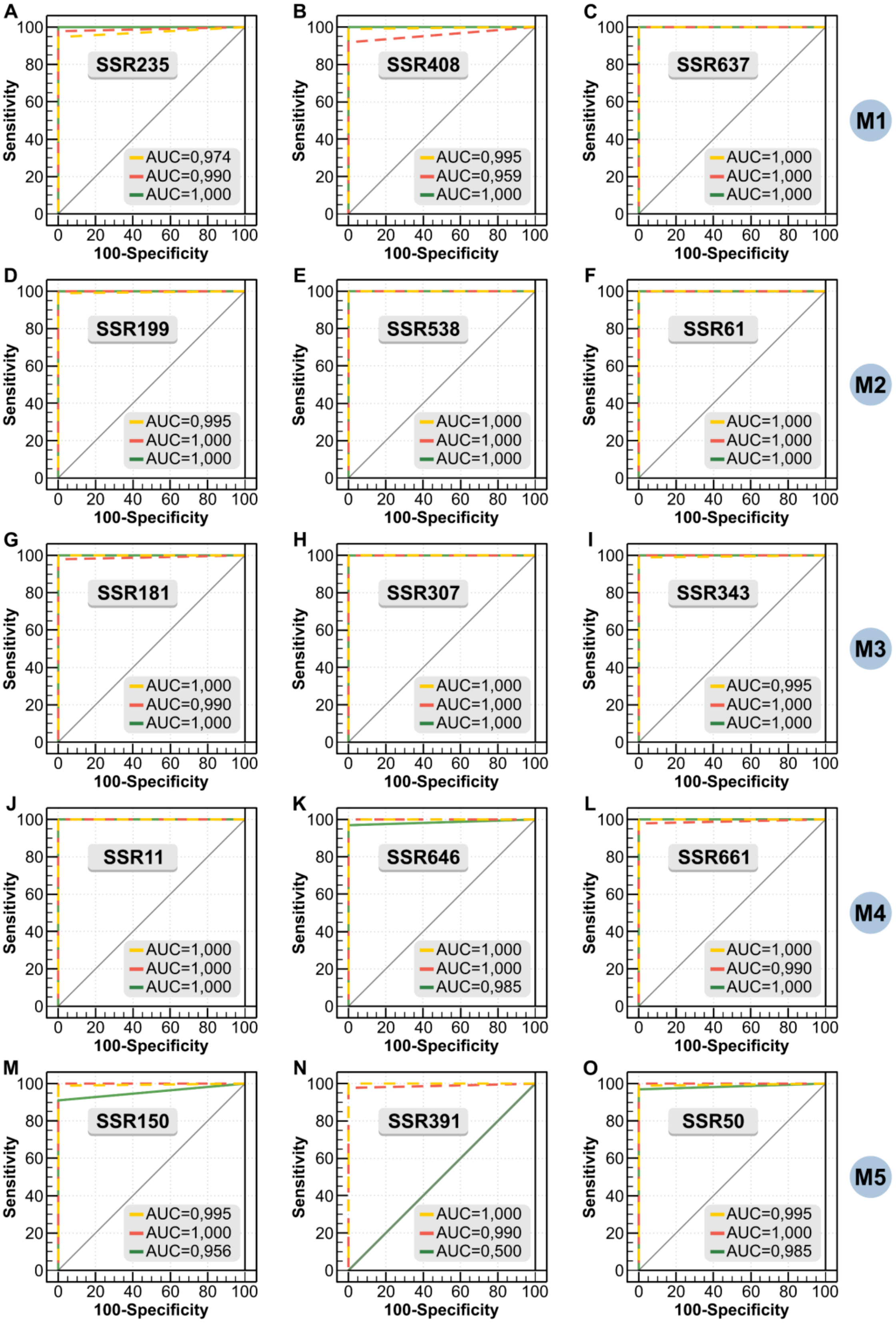

Supplementary Figure S2. Summary of performance of SSR markers for *S. brasiliensis* (n=97, yellow line), *S. schenckii* (n=47, red line), and *S. globosa* (n=37, green line) using receiver operating characteristic curves. The area under the ROC curve was great (AUC above 0.956) for all primer pairs (except marker SSR391 for *S. globosa* isolates), indicating excellent performance.

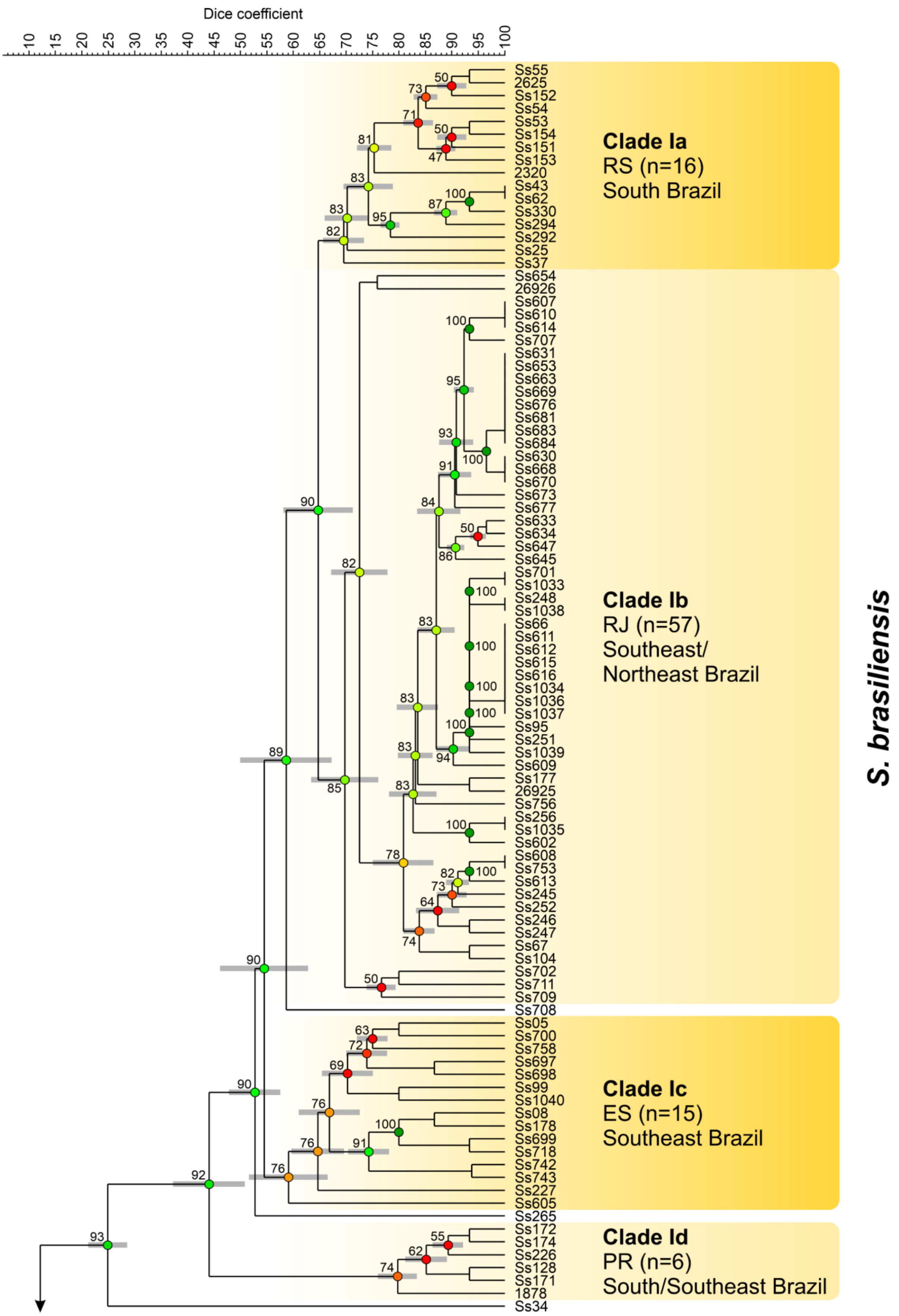
Dice coefficient
10 15 20 25 30 35 40 45 50 55 60 65 70 75 80 85 90 95 100
Ss55
2625
Ss152
Ss54
Ss53
Ss154
Ss151
Ss153
2320
Ss43
Ss62
Ss330
Ss294
Ss292
Ss25
Ss37
Clade Ia
RS (n=16)
South Brazil
Ss654
26926
Ss607
Ss610
Ss614
Ss707
Ss631
Ss653
Ss663
Ss669
Ss676
Ss681
Ss683
Ss684
Ss630
Ss668
Ss670
Ss673
Ss677
Ss633
Ss634
Ss647
Ss645
Ss701
Ss1033
Ss248
Ss1038
Ss66
Ss611
Ss612
Ss615
Ss616
Ss1034
Ss1036
Ss1037
Ss95
Ss251
Ss1039
Ss609
Ss177
26925
Ss756
Ss256
Ss1035
Ss602
Ss608
Ss753
Ss613
Ss245
Ss252
Ss246
Ss247
Ss67
Ss104
Ss702
Ss711
Ss709
Ss708
Clade Ib
RJ (n=57)
Southeast/
Northeast Brazil
Ss05
Ss700
Ss758
Ss697
Ss698
Ss99
Ss1040
Ss08
Ss178
Ss699
Ss718
Ss742
Ss743
Ss227
Ss605
Clade Ic
ES (n=15)
Southeast Brazil
Ss265
Ss172
Ss174
Ss226
Ss128
Ss171
1878
Clade Id
PR (n=6)
South/Southeast Brazil
Ss34
S. brasiliensis

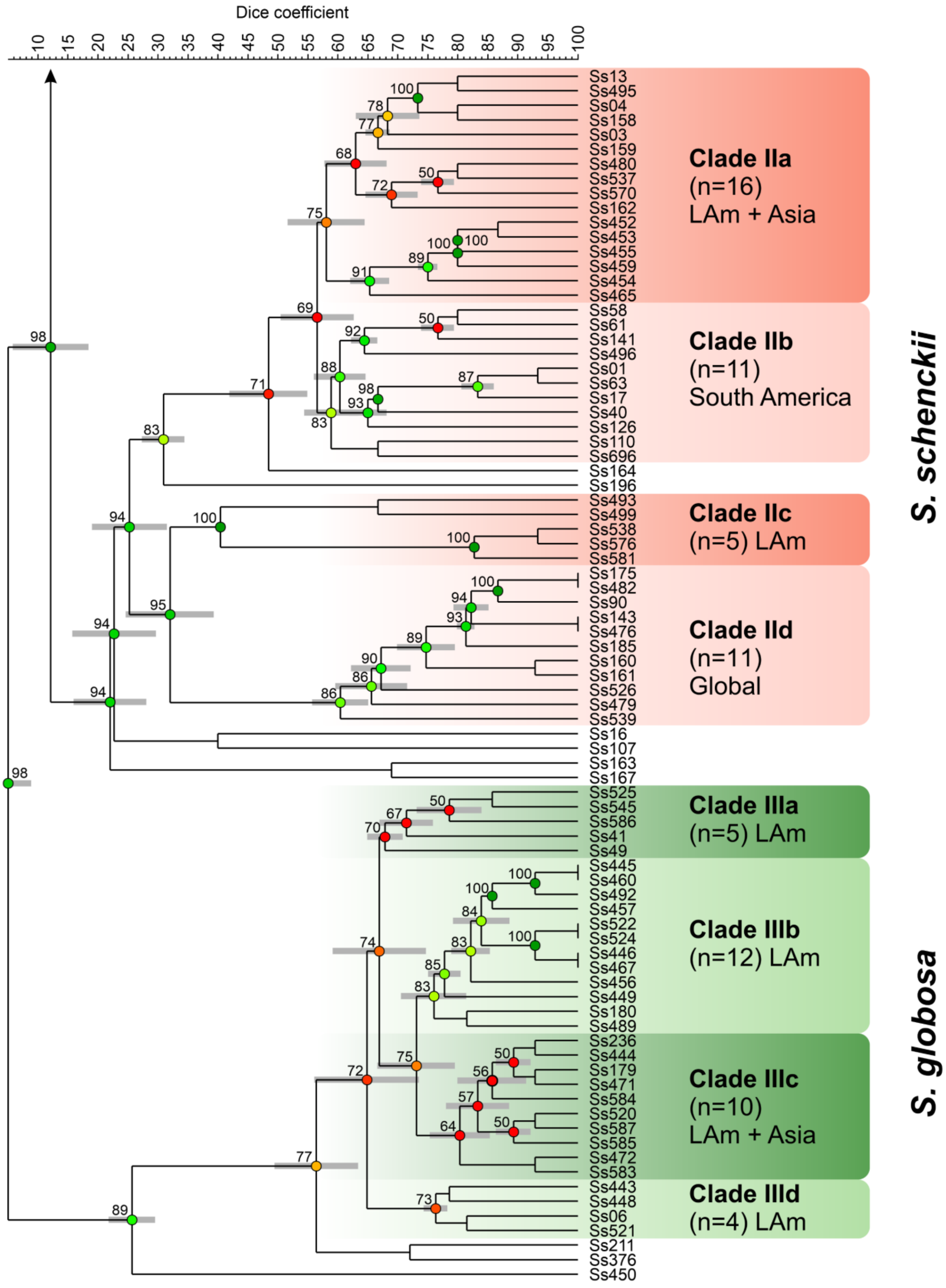

Supplementary Figure S3. The annotated UPGMA dendrogram (clades), based on SSR markers, generated with a total of 15 SSR markers for 180 *Sporothrix* isolates originated worldwide. The dendrogram shows cophenetic correlation values (circles are represented by color ranges between green-yellow-orange-red according to decreasing cophenetic correlation) for a given clade and its standard deviation (grey bar). For pairwise genetic distances calculation, the Dice similarity coefficient was used.

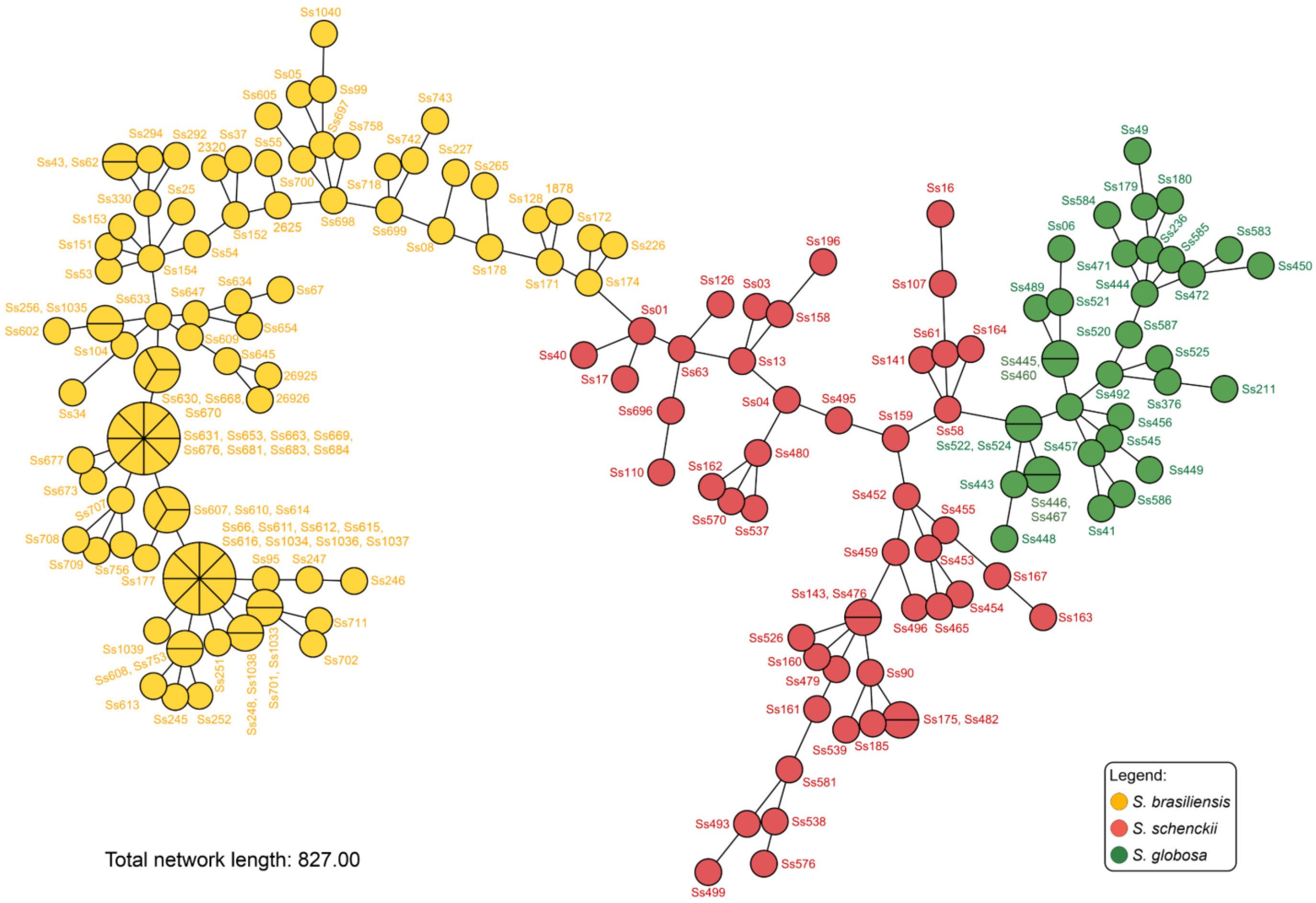

Supplementary Figure S4. Annotated Minimum Spanning Trees (MSTs) showing the genetic relationship among 180 *Sporothrix* isolates using 15 SSRs markers. MST was created in the software BioNumerics v7.6.

Supplementary References

1. de Carvalho, J.A.; Beale, M.A.; Hagen, F.; Fisher, M.C.; Kano, R.; Bonifaz, A.; Toriello, C.; Negroni, R.; Rego, R.S.M.; Gremiao, I.D.F.; et al. Trends in the molecular epidemiology and population genetics of emerging *Sporothrix* species. *Stud Mycol* **2021**, *100*, 100129, doi:10.1016/j.simyco.2021.100129.
2. Rodrigues, A.M.; de Hoog, G.S.; de Camargo, Z.P. Molecular diagnosis of pathogenic *Sporothrix* species. *PLoS Negl Trop Dis* **2015**, *9*, e0004190, doi:10.1371/journal.pntd.0004190.
3. Rodrigues, A.M.; de Hoog, G.S.; Zhang, Y.; Camargo, Z.P. Emerging sporotrichosis is driven by clonal and recombinant *Sporothrix* species. *Emerg Microbes Infect* **2014**, *3*, e32, doi:10.1038/emi.2014.33.
4. de Carvalho, J.A.; Pinheiro, B.G.; Hagen, F.; Goncalves, S.S.; Negroni, R.; Kano, R.; Bonifaz, A.; de Camargo, Z.P.; Rodrigues, A.M. A new duplex PCR assay for the rapid screening of mating-type idiomorphs of pathogenic *Sporothrix* species. *Fungal Biol* **2021**, *125*, 834-843, doi:10.1016/j.funbio.2021.05.005.
5. Rodrigues, A.M.; de Melo Teixeira, M.; de Hoog, G.S.; Schubach, T.M.P.; Pereira, S.A.; Fernandes, G.F.; Bezerra, L.M.L.; Felipe, M.S.; de Camargo, Z.P. Phylogenetic analysis reveals a high prevalence of *Sporothrix brasiliensis* in feline sporotrichosis outbreaks. *PLoS Negl Trop Dis* **2013**, *7*, e2281, doi:10.1371/journal.pntd.0002281.

6. Della Terra, P.P.; Gonsales, F.F.; de Carvalho, J.A.; Hagen, F.; Kano, R.; Bonifaz, A.; Camargo, Z.P.; Rodrigues, A.M. Development and evaluation of a multiplex qPCR assay for rapid diagnostics of emerging sporotrichosis. *Transbound Emerg Dis* **2022**, *69*, e704-e716, doi:10.1111/tbed.14350.
7. Teixeira, M.M.; de Almeida, L.G.; Kubitschek-Barreira, P.; Alves, F.L.; Kioshima, E.S.; Abadio, A.K.; Fernandes, L.; Derengowski, L.S.; Ferreira, K.S.; Souza, R.C.; et al. Comparative genomics of the major fungal agents of human and animal Sporotrichosis: *Sporothrix schenckii* and *Sporothrix brasiliensis*. *BMC Genom* **2014**, *15*, 943, doi:10.1186/1471-2164-15-943.
8. Cuomo, C.A.; Rodriguez-Del Valle, N.; Perez-Sanchez, L.; Abouelleil, A.; Goldberg, J.; Young, S.; Zeng, Q.; Birren, B.W. Genome sequence of the pathogenic fungus *Sporothrix schenckii* (ATCC 58251). *Genome Announc* **2014**, *2*, doi:10.1128/genomeA.00446-14.
9. Gomez, O.M.; Alvarez, L.C.; Muñoz, J.F.; Misas, E.; Gallo, J.E.; Jimenez, M.D.P.; Arango, M.; McEwen, J.G.; Hernandez, O.; Clay, O.K. Draft genome sequences of two *Sporothrix schenckii* clinical isolates associated with human sporotrichosis in Colombia. *Genome Announc* **2018**, *6*, e00495-00418, doi:10.1128/genomeA.00495-18.
10. Huang, L.; Gao, W.; Giosa, D.; Criseo, G.; Zhang, J.; He, T.; Huang, X.; Sun, J.; Sun, Y.; Huang, J.; et al. Whole-genome sequencing and in silico analysis of two strains of *Sporothrix globosa*. *Genome Biol Evol* **2016**, *8*, 3292-3296, doi:10.1093/gbe/evw230.
11. D'Alessandro, E.; Giosa, D.; Huang, L.; Zhang, J.; Gao, W.; Brankovics, B.; Oliveira, M.M.E.; Scordino, F.; Lo Passo, C.; Criseo, G.; et al. Draft genome sequence of the dimorphic fungus *Sporothrix pallida*, a nonpathogenic species belonging to *Sporothrix*, a genus containing agents of human and feline sporotrichosis. *Genome Announc* **2016**, *4*, e00184-00116, doi:10.1128/genomeA.00184-16.
